# “We have already heard that the treatment doesn't do anything, so why should we take it?”: A mixed method perspective on Chagas disease knowledge, attitudes, prevention, and treatment behaviour in the Bolivian Chaco

**DOI:** 10.1371/journal.pntd.0008752

**Published:** 2020-10-29

**Authors:** Sandra Parisi, Miriam Navarro, Jeremy Douglas Du Plessis, Jonathan Phillip Shock, Boris Apodaca Michel, Minerva Lucuy Espinoza, Carolina Terán, Nino Antonio Calizaya Tapia, Katharina Oltmanns, Abundio Baptista Mora, Claudia Saveedra Irala, Angel Alberto Rivera Rojas, Gonzalo Rubilar, Thomas Zoller, Michael Pritsch

**Affiliations:** 1 Institute of Tropical Medicine and International Health, Charité –Universitätsmedizin Berlin, Berlin, Germany; 2 DAHW Deutsche Lepra- und Tuberkulosehilfe e. V., Würzburg, Germany; 3 Department of General Practice, Universitätsklinikum Würzburg, Würzburg, Germany; 4 Department of Public Health, Science History and Gynecology, Universidad Miguel Hernández, Alicante, Spain; 5 Department of Maths and Applied Maths, University of Cape Town, Rondebosch, South Africa; 6 Hospital Dermatológico Monteagudo, Monteagudo, Bolivia; 7 Fundacion Intercultural NORSUD, Sucre, Bolivia; 8 DAHW Asociación Alemana de Asistencia al Enfermo con Lepra y Tuberculosis, Oficinas Suramérica, Bogotá, Colombia; 9 Universidad Mayor, Real y Pontificia de San Francisco Xavier de Chuquisaca, Faculty of Medicine, Sucre, Bolivia; 10 Charité –Universitätsmedizin Berlin, corporate member of Freie Universität Berlin, Humboldt-Universität zu Berlin, and Berlin Institute of Health, Berlin, Germany; 11 Department of Infectious Diseases and Respiratory Medicine, Charité –Universitätsmedizin Berlin, Berlin, Germany; 12 Division of Infectious Diseases and Tropical Medicine, LMU University Hospital Munich, Munich, Germany; Universidade Federal de Minas Gerais, BRAZIL

## Abstract

**Background:**

Chagas disease (CD) is highly endemic in the Bolivian Chaco. The municipality of Monteagudo has been targeted by national interventions as well as by Médecins Sans Frontières to reduce infection rates, and to decentralize early diagnosis and treatment. This study seeks to determine the knowledge and attitudes of a population with increased awareness and to identify remaining factors and barriers for sustained vector control, health care seeking behaviour, and access, in order to improve future interventions.

**Methodology/Principal findings:**

A cross-sectional survey was conducted among approximately 10% (n = 669) of the municipality of Monteagudo’s households that were randomly selected. Additionally, a total of 14 in-depth interviews and 2 focus group discussions were conducted with patients and key informants. Several attitudes and practices were identified that could undermine effective control against (re-)infection. Knowledge of clinical symptoms and secondary prevention was limited, and revealed specific misconceptions. Although 76% of the participants had been tested for CD, only 18% of those who tested positive concluded treatment with benznidazole (BNZ). Sustained positive serologies after treatment led to perceived ineffectiveness of BNZ. Moreover, access barriers such as direct as well as indirect costs, BNZ stock-outs and a fear of adverse reactions triggered by other community members made patients opt for alternative treatments against CD such as veterinary ivermectin, used by 28% of infected participants in our study. The lack of accessible care for chronic complications as well as socioeconomic consequences, such as the exclusion from both job opportunities and bank loans contributed to the ongoing burden of CD.

**Conclusions/Significance:**

Large scale interventions should be accompanied by operational research in order to identify misconceptions and unintended consequences early on, to generate accessible data for future interventions, and for rigorous evaluation. An integrated, community-based approach tackling social determinants and including both traditional and animal health sectors might help to overcome current barriers and advocate for patients’ rights.

## Author summary

Chagas disease (CD) is caused by infection with the parasite *Trypanosoma cruzi* and is on the list of the most neglected tropical diseases on Earth. It has become an international health issue affecting 6–7 million people worldwide, mainly in Latin America. We performed a qualitative and quantitative study in Monteagudo, Bolivia, in order to paint an overall picture of CD in one of the most afflicted regions in the world: the Bolivian Chaco. Community members and key informants, such as patients and healthcare staff helped us to understand their current situation. Even after a large health campaign, persistent knowledge gaps, misconceptions and structural barriers were identified, leading to normalization and acceptance of CD and its social consequences. Relevant findings included the broad use of ivermectin for CD treatment without scientific evidence. We also found that misinterpretations of serologic test results contribute to a critical attitude towards biomedical treatment that is stopping patients from seeking treatment. With this research we aim to update the data on current knowledge and to analyse remaining barriers for transmission control and treatment, in order to focus future interventions and advocate for patient’s rights.

## Introduction

Chagas disease (CD) is an intriguingly complex, polymorphic and highly successful parasitic disease caused by *Trypanosoma* (*T*.) *cruzi*. There are many factors which make treatment and prevention particularly difficult. These include the associated stigma of the disease, poverty, the 100+ species of mammals which act as hosts/reservoirs as well as possible vectors, a domestic and sylvatic life-cycle, the many transmission routes, the insufficient epidemiological data, the lack of concrete biomarkers for treatment evaluation and disease progression, the high genetic diversity of the parasite, the polymorphic clinical presentations, the absence of satisfactory treatment options, and the lack of awareness about the disease by both patients and healthcare professionals [1–3]. The current tools for control seem insufficient and CD has become an international health problem due to migration and globalization [4]. Belonging to the 20 so-called neglected tropical diseases (NTDs) designated by the World Health Organization (WHO), it is one of the “most neglected”, as—although no official numbers exist—less than 1% of the estimated 6 million people infected are thought to have access to adequate diagnosis and treatment [5]. Furthermore, it creates a greater burden of mortality and disability-adjusted life years (DALYs) than any other parasitic disease in the Americas [6]. The municipality of Monteagudo lies in the heart of the Bolivian Chaco. This region exhibits high prevalence rates and CD-related morbidity without having adequate access to care for the chronically ill [7]. Since 2006, CD has been declared a national priority by law in Bolivia and several activities have been implemented by the National Chagas Program (NCP) using benznidazole (BNZ) as the first-line treatment [8,9]. In the years 2014–2016, the non-governmental organization (NGO) Médecins Sans Frontières (MSF) carried out a program for integrated care of CD in Monteagudo to prove the feasibility of decentralizing early diagnosis and treatment [10]. Despite all this, according to local experience, treatment coverage appears to remain low, but has not systematically been assessed in Monteagudo since MSF left the area. Moreover, availability as well as access to BNZ continues to be a major problem [8] and vector infestation rates remain high in some communities of Monteagudo causing an ongoing risk for vectorial transmission [11,12].

There are several steps from initial awareness and theoretical knowledge about a disease to the adoption of individual preventive behaviors [13–15]. For the implementation of sustainable interventions, the setting needs to be thoroughly understood and the community actively involved. However, there is a lack of up-to-date data on knowledge, perceptions, practices and experiences concerning CD from the study area [16], as well as factors for adoption of preventative measures such as early diagnosis and treatment [17]. Through assessing the local situation in the municipality of Monteagudo two years after the interventions of MSF, additional factors relevant for sustained control as well as protective behaviours within communities should be identified, and valuable lessons learned for future projects.

## Methods

### Study site and study population

**Fig 1:**
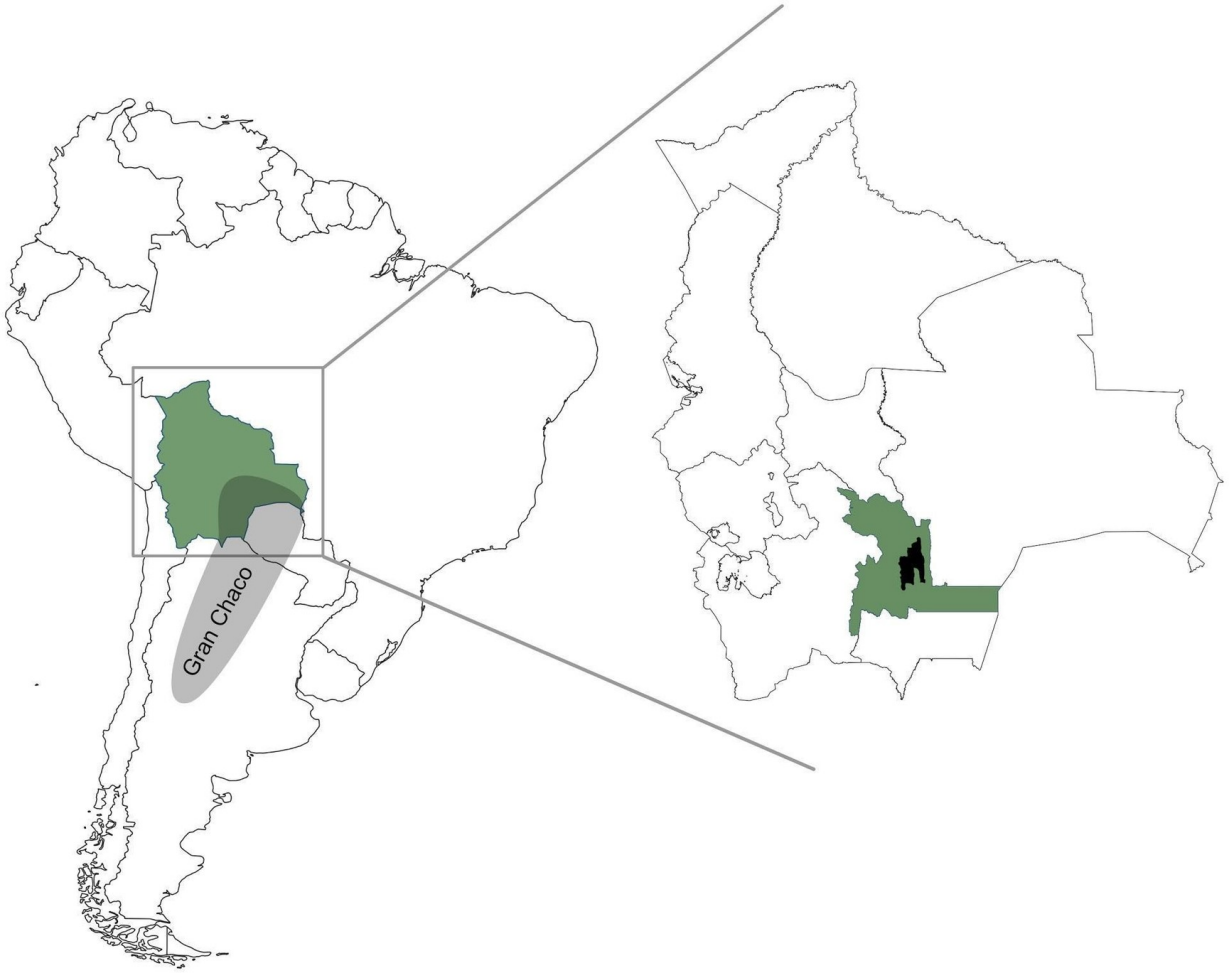
Geographical setting of the study. Bolivia (green) and the Gran Chaco region (grey) are depicted on the left. Departments of Bolivia with the department of Chuquisaca (green) and the municipality of Monteagudo (black) are highlighted on the right.

This study was located in the municipality of Monteagudo, belonging to the district Chuquisaca in the Bolivian Chaco region (Fig 1).

According to an official census performed in 2012, the municipality of Monteagudo consisted of 24,303 inhabitants [18], of which approximately 60% live in the town of Monteagudo and 40% in dispersed rural communities. Some communities are very remote, inaccessible by car and at times completely unreachable due to challenging weather conditions. A large proportion of the population works in the informal sector without access to health insurance. Monteagudo has been the site of an MSF project on CD in the years 2014–2016 that included information, testing and treatment campaigns, fostering of the community-wide network for vector control as well as the systematization of case notification and data collection which was conveyed to the national authorities [10,19]. According to data registered by MSF in cooperation with the Ministry of Health in 2016, a total of 2,261 inhabitants >15 years (17% of the total population) were tested for CD showing an overall prevalence rate of 51.8% [20]. Prevalence of CD among pregnant women was 36.8% (259/702) in 2016 and 44.2% (279/631) in 2017, with vertical transmission rates of 2.7% and 3.0%, respectively [21,22].

### Ethics

The study protocol was approved by the Institutional Review Board at the Ludwig-Maximilians-University in Munich, Germany (opinion dated September 25^th^ 2018, number 18–686) and the University Mayor, Real y Pontificia de San Francisco Xavier de Chuquisaca in Sucre, Bolivia (opinion dated August 23^rd^ 2018). All included participants were adults that signed informed consent.

### Quantitative interviews

All study participants were interviewed using a standardized questionnaire (see S1 Text) for the collection of socioeconomic, medical and other personal data. All households in the study area had been mapped by the NCP with a total of 6,802 households being listed, divided into 97 rural communities and urban districts that correspond to a total of 17 healthcare centers (HC) [12]. For the selection of a representative sample, we initially stratified all communities according to the 17 HC of the municipality and calculated the proportional number of households to be included according to population density within each HC. In a second step we randomly selected 1–3 communities until covering the required number of households. In order to account for potential logistic risks, one additional community in each catchment area was added as an alternative option. Households were then subsequently selected in a random manner as third step, using lists of all existing community households in these selected communities. The required sample size was calculated to be 364 households using EPIDAT 4.2 (Xunta de Galicia, OPS-OMS) considering the total number of 6,802 households and a presumed prevalence of relevant knowledge and relevant level of intention to engage in preventative activities of 50% as well as a CI of 95%. In order to include sufficient households in remote areas with low population density, we increased the sample size to approximately 10% of the municipality’s households, maintaining its proportional distribution. Inclusion criteria were age ≥18 years and permanent residency (minimum six months prior to the study). Exclusion criteria were absence during study visit or refusal to take part. Whenever a selected household was found empty, it was replaced by the nearest household to ensure representative geographic coverage within the communities.

### Qualitative research

In-depth interviews (IDIs) and focus group discussions (FGDs) were conducted up to the point of data saturation. An interview guide was used (S2 Text). Participants were adults who signed informed consent, chosen from 3 main groups: (i) patients with a confirmed CD diagnosis, (ii) family members of CD patients and (iii) key informants (including healthcare staff, government officials, traditional healers, community leaders, etc.). Other selection criteria for participants depended on themes arising during the data collection process. Each FGD consisted of 6–8 participants from a similar background. Observation field notes were employed as a complementary technique.

### Quantitative data analyses

Data generated from responses to questionnaires were recorded by double data entry into Microsoft Excel 2013 tables (Microsoft Corporation, Redmond, WA, USA). Data stored in these tables were imported into, and subsequently analysed, using Python 3.6 in the Jupyter Notebook environment. The Pandas 0.23.0, Numpy 1.14.6, Scipy 1.2.1, Imblearn 0.0 and Sklearn 0.0 software libraries were used for analyses, and Matplotlib 3.0.3 and Seaborn 0.9.0 software libraries were used for data visualisation. In order to determine whether or not there were significant differences in responses to various qualitative questions (with categorical responses) across socio-economic categories, p-values were computed using a chi-squared test.

### Protection motivation theory (PMT) analyses

Questions were grouped into five categories according to the PMT framework [15,23] pertaining to respondents’ (i) perceived severity of CD, (ii) perceived vulnerability with respect to CD, (iii) perceived response efficacy (whether or not preventive measures are considered effective against the health threat), (iv) perceived self-efficacy (whether or not the respondent feels able to adapt and maintain preventive behaviour), and (v) perceived response cost (necessary effort/negative consequences of adapting preventive behaviour).

### Qualitative data analyses

All FGDs and IDIs were recorded, transcribed and underwent quality control. Methodological (within-method) and investigator triangulation was performed by three researchers of different professional and cultural backgrounds. A deductive-inductive approach was used for analyses with predefined codes aligned to the survey, dimensions of access [24] and the PMT model including integration of quantitative data. Emerging themes, codes, and integration of results were then discussed among the analysing researchers allowing for reflection on different interpretations, attitudes and hypotheses. Additionally, primary data was triangulated with the insights gained during many observations and non-structured interviews over the period of a three-month stay among local communities by one of the researchers.

## Results

### Quantitative interviews

**Fig 2:**
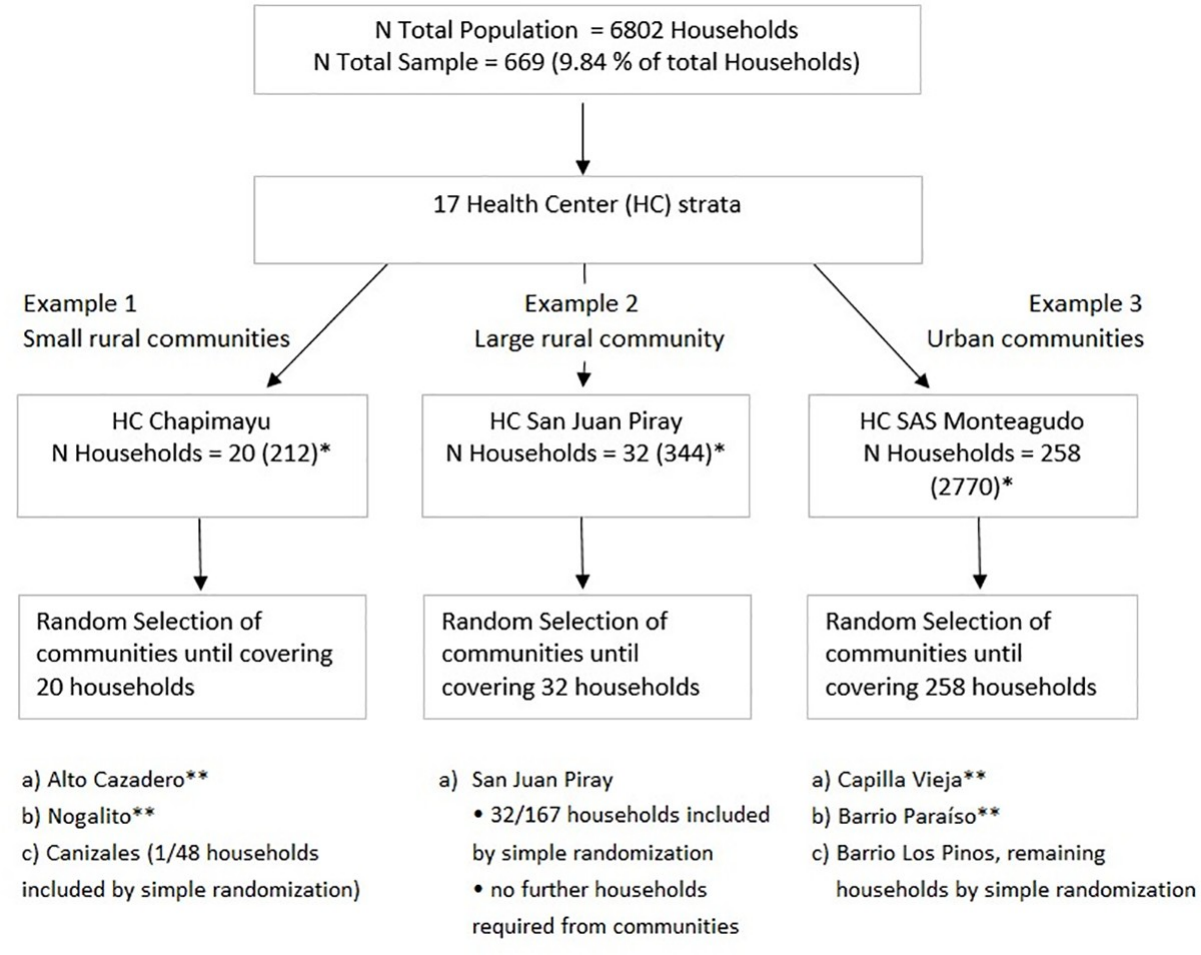
Flow chart of multistage stratified random sampling with examples for different settings encountered. *The existing number of households, especially in small communities, was often lower than provided by the official census NCP data, as these also contain abandoned households. Small variations in sampling were partly due to logistic reasons. **All encountered households were included.

**Fig 3:**
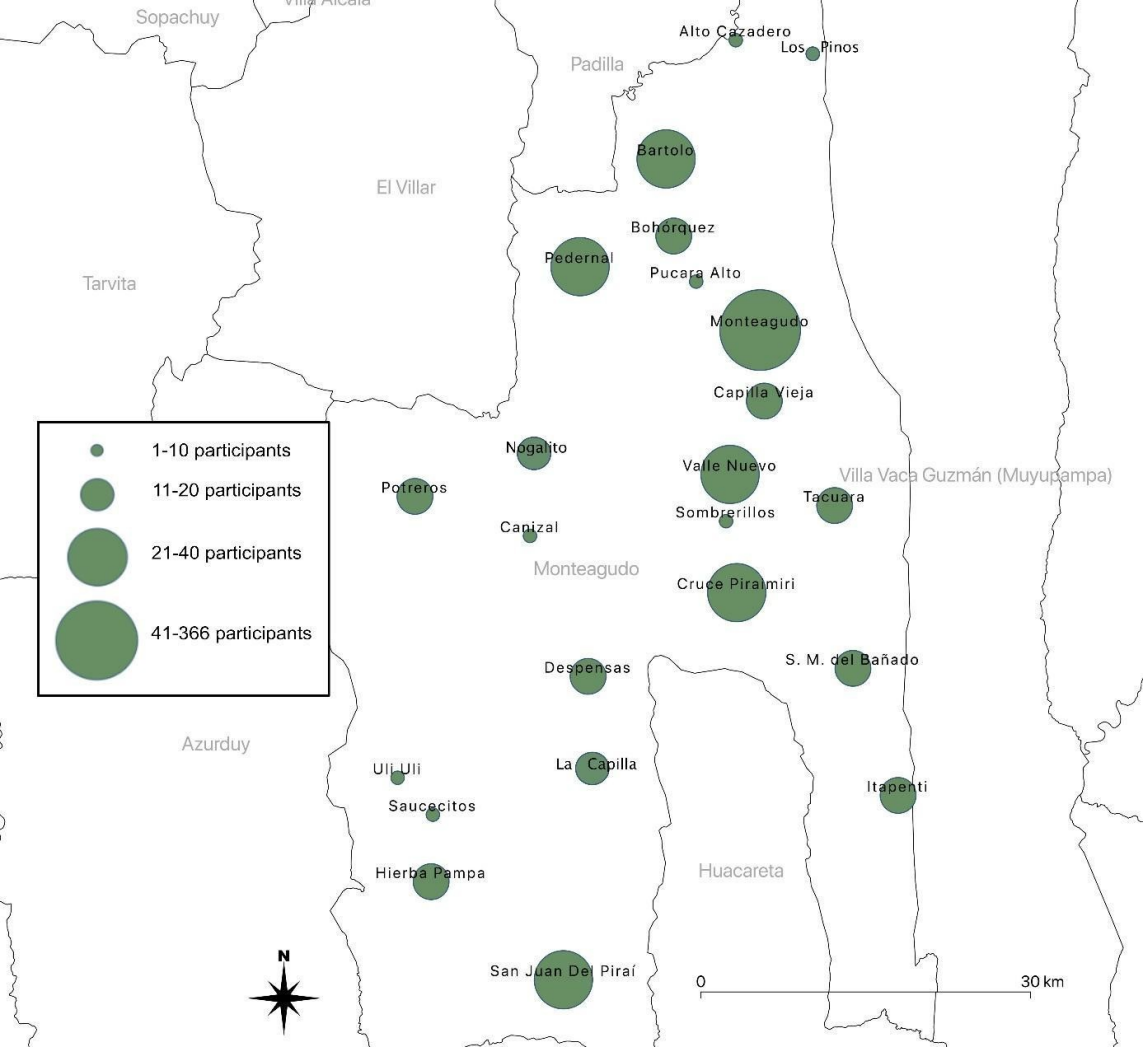
Geographic distribution of included communities. A total of 26 communities were included in this study. Sizes of dots represent the respective number of households in each community.

A total of 26 communities were randomly selected (Figs 2 and 3; S1 Table). One community had to be replaced by an alternative option due to rain which made the roads inaccessible. In addition, we purposefully included one community with ongoing high risk of vectorial transmission despite repeated interventions of the NCP. Included communities ranged from 1 to 142 households and covered the whole geographic area of the municipality of Monteagudo (Figs 2 and 3; S1 Table). The domiciliary infestation index recorded by the NCP in 2017 ranged from 0.0% to 31.8% for the included communities [11]. The detailed description of included communities can be found in S1 Table.

In 2018, from October 12th to November 29th quantitative interviews were conducted in 669 households, thus comprising 9.8% of total households in Monteagudo (Table 1). By increasing the sample size, we assured the inclusion of a representative amount of very remote and inaccessible communities and households, facing different social determinants than more urban communities while maintaining its proportional distribution [25]. The study was very well received by communities; no household refused participation. Questionnaires were filled out by trained interviewers with a medical background (final year medical students) while interviewing the participants. Prior to data collection, the questionnaire was pre-tested at San Miguel de las Pampas, Monteagudo.

**Table 1:** Included participants. Selected socio-economic data on included household representatives.

Sex*	Number	% (N = 669)	Health insurance*	Number	% (N = 669)
Female	412	61.8	Yes	232	34.7
Male	255	38.2	No	436	65.3
Age*	Number	% (N = 669)	Language mostly spoken	Number	% (N = 669)
18–25	95	14.2	Spanish	631	94.3
26–40	176	26.3	Quechua	4	0.6
41–60	248	37.1	Spanish and Quechua	31	4.6
61–93	147	22.0	Spanish and Guaraní	3	0.4
Highest educational level*	Number	% (N = 669)	Profession*	Number	% (N = 669)
None	68	10.2	Household	275	41.4
Incomplete primary school	244	36.7	Farming	151	22.7
Complete primary school	62	9.3	Student	47	7.1
Incomplete secondary school	46	6.9	Teacher	38	5.7
Complete secondary school	120	18.0	Job within health care**	22	3.3
Technical or superior studies	62	9.3	Other	131	19.7
Academic	63	9.5			

* Non-respondents: sex = 2 (0.3%), age = 3 (0.5%), education = 4 (0.6%), health insurance = 1 (0.2%), profession = 5 (0.7%).

**”Job within health care” included health care staff and people that were sought for professional advice with regard to CD (e.g. traditional healers, veterinarians, pharmacists, jobs involved in CD control).

### Qualitative interviews

A total of 14 IDIs and 2 FGDs were held with an average duration of 30 minutes from September 27th to December 1st 2018. These included patients with diverse clinical and treatment backgrounds, and key informants from both remote and urban settings. Deliberate sampling of specific types of participants such as a veterinary doctor and young adults, as well as the integration of new topics into the survey allowed for a deeper understanding of arising themes (S2 Text and S2 Table).

### Information sources and level of knowledge on CD

Most participants had good general awareness on CD and cited several information sources (V1-3/S2 Table, S3 Table). Patients often sought a wide range of suggestions in order to find a cure which sometimes caused severe uncertainties as to what to do. The experience of someone affected by CD was often more influential than the information obtained at healthcare services (V4-6/S2 Table). Vectorial transmission was widely-known (90.4%, 605/669), whereas vertical (36.9% 247/669), blood/organ donation (26.0%, 174/669) and oral transmission (6.1%, 41/669) was less frequently cited (S3 Table). Knowledge of clinical symptoms was often limited to the awareness of heart or intestinal involvement, and that it can lead to death. Knowledge of acute symptoms was almost absent and only 25.9% (173/669) knew that chronic symptoms could manifest over a decade after infection (V7-8/S2 Table, S3 Table). The majority of respondents could cite primary preventive measures against CD, such as cleaning and improving housing conditions (61.6%), as well as using insecticides (49.5%) (V10-11/S2 Table, S3 Table), which, however, also included self-administered local products with dubious efficacy (V19, V26/S2 Table). Secondary preventive measure such as early diagnosis (15.2%), pregnancy screening (8.2%) and treatment (19.6%) were less frequently cited and included alternative medicine without scientific evidence for effectiveness, such as veterinary ivermectin (16.3%) and *cumanda* beans (17.5%) (V12, V25, V32-34/S2 Table, S3 Table).

### Perceptions, intentions and attitudes

#### PMT- threat appraisal

**Fig 4:**
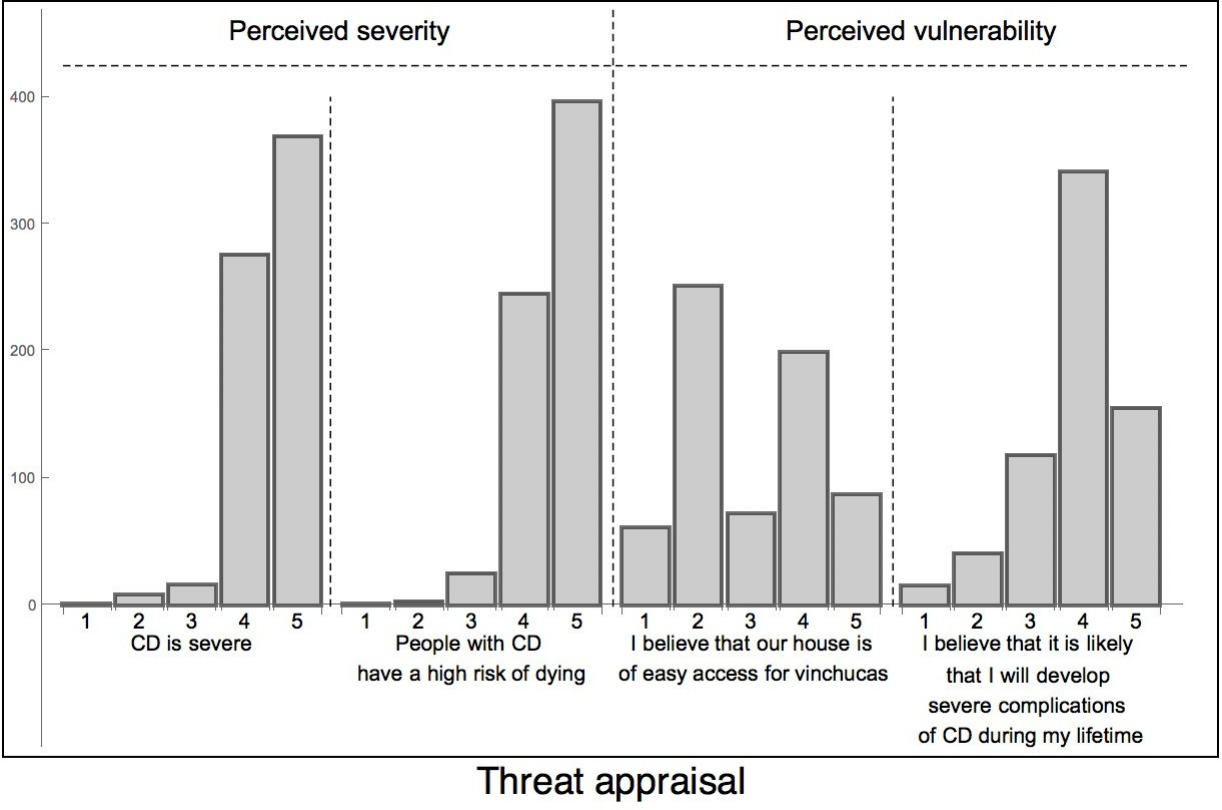
Attitudes of household representatives towards CD related to perceived severity and vulnerability. Responses aligned to the PMT threat appraisal pathway. Survey responses on a 1–5 point adapted Likert scale (1 = Strongly disagree, 2 = Disagree, 3 = Don´t know, 4 = Agree, 5 = Strongly agree).

Most participants perceived CD to be severe/very severe (96.3%, 644/669; Fig 4) and associated CD with a sudden, unexpected death (V14/S2 Table, Fig 4). The slow onset of the disease and its high prevalence (making it “normal”) were reasons for not taking it seriously (V15-16/S2 Table). The belief that the risk of getting infected nowadays has significantly decreased due to a reduced number of vectors, appeared to lead to a false sense of security (V17-18/S2 Table).

#### PMT- coping appraisal

**Fig 5:**
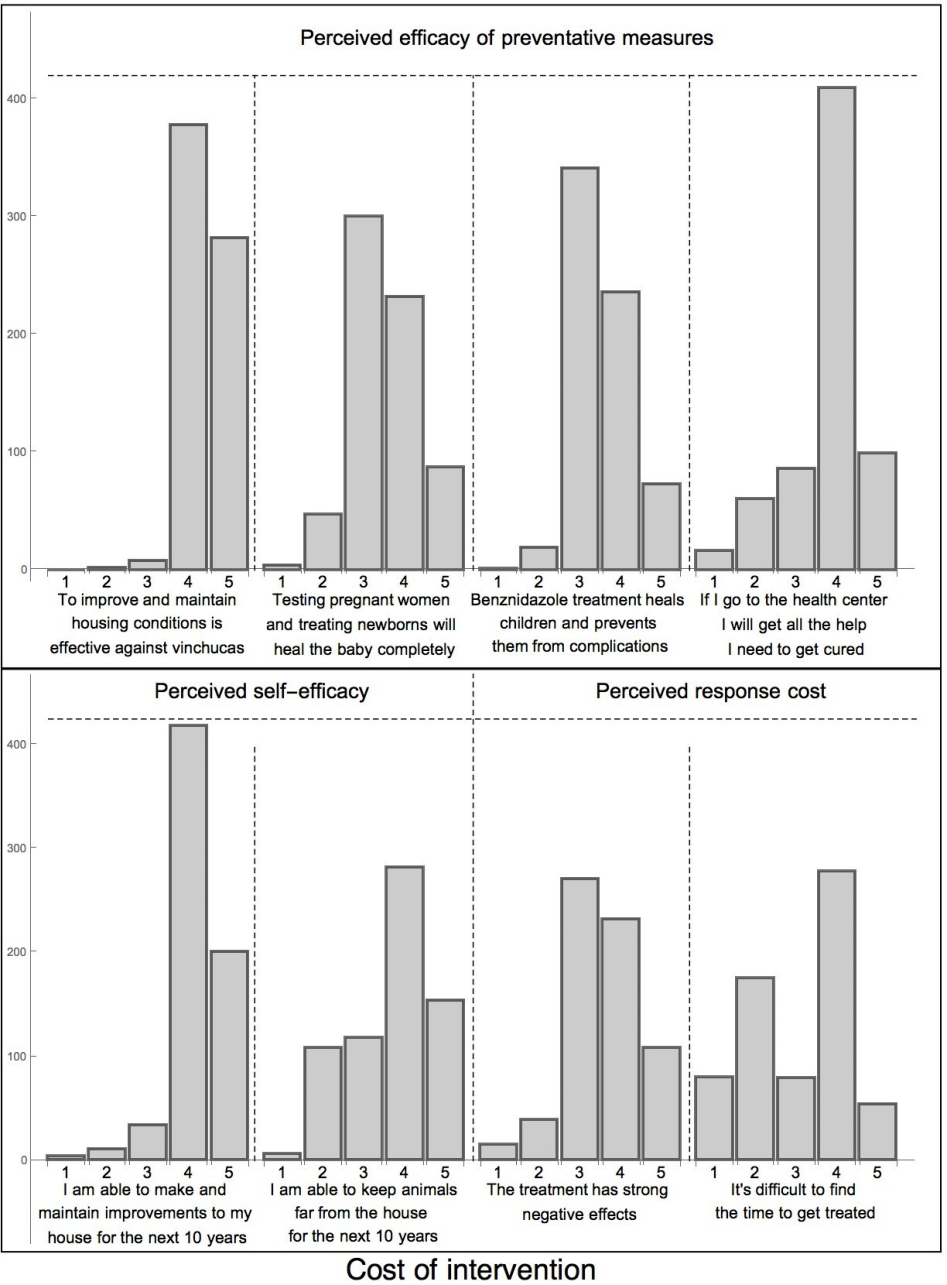
Attitudes of household representatives towards CD related to perceived effectiveness, self-efficacy and response cost. Responses aligned to the PMT coping appraisal pathway. Survey responses on a 1–5 point adapted Likert scale (1 = Strongly disagree, 2 = Disagree, 3 = Don´t know, 4 = Agree, 5 = Strongly agree).

The most challenging preventative measure reported was in keeping animals away from the house. Perceived effectiveness varied largely between primary and secondary preventive measures (S4 Table, Fig 5). Strong trust in primary preventive measures could, for instance, lead to self-organized community campaigns if the response of authorities was not considered fast enough (V19, V26/S2 Table). However, doubts related to the effectiveness of anti-parasitic treatment were frequent and only about half of the respondents believed in treatment efficacy in newborns and children. A positive post-treatment test based on serology was often misunderstood as treatment failure and antibody levels were frequently used as indicators for state/progression of the disease and after different treatments (V20-21, V23/S2 Table). Treatment efficacy was also evaluated on the immediate improvements of symptoms and, therefore, the self-administered ivermectin was often considered more effective (V25, V34/S2 Table), possibly due to its broad-spectrum effect against other parasites. Another emerging theme was that BNZ was only considered one of many equally valid treatment options, associated with higher direct and indirect costs than alternative medicines which are easily available and have a much shorter treatment duration. In addition to hurdles such as long distances, economic and time constraints associated with pre-treatment diagnostics and long treatment duration, the fear of severe side effects was frequently cited (V22/S2 Table). The locally recommended strict diet and the prevention of sun exposure during treatment were other perceived costs which are not compatible with work or social life (V24/S2 Table). In the end this negative cost-benefit balance often caused participants to lean towards other treatment options (V21/S2 Table; Fig 5).

#### Practices and preventive action

Most of the participants (59.3%; 397/669) indicated that they used at least one primary preventive measure against vector presence (S4 Table). More than a quarter of the respondents (25.9%, 173/669) had already reported vector presence to the respective vector control office and details on responses can be found in Table 2. Some practices that could undermine primary prevention were identified, such as using common insecticides of dubious effectiveness, living in close proximity with animals, and maintaining old, infested houses, despite the construction of improved ones (V26-27/S2 Table; S4 Table; Table 2).

**Table 2:** Self-reported vector presence and responses of the National Chagas Programme.

Self-reported vector presence	Number of participants	Percentage of participants (N = 669)
Current vector presence at the house	72	10.8
Unsure of current vector presence	10	1.5
Vector presence at the house during last year	186	27.8
Previous report of vector presence to authorities	173	25.9
Response of NCP	Number of participants	Percentage of participants (N = 173)
NCP came to spray	107	61.9
No response by NCP	48	27.8
Inconclusive response	18	10.4

#### Secondary prevention—early diagnosis and treatment for CD

**Fig 6:**
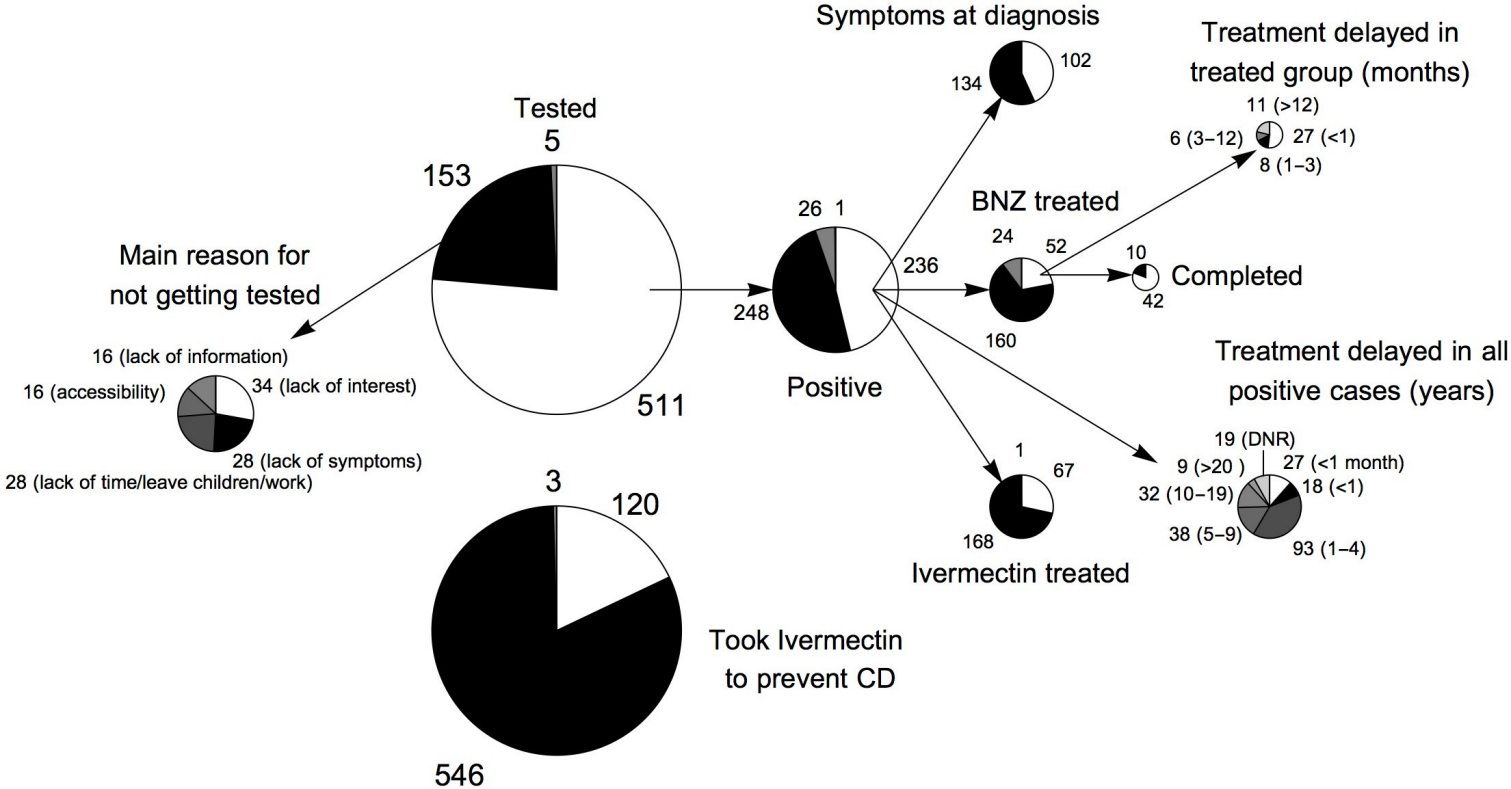
Secondary prevention of CD: Diagnosis and treatment. For those where no key is given, white corresponds to “yes”, black to “no”, grey corresponds to “don’t know”, and anything else is “no answer”.

**Fig 7:**
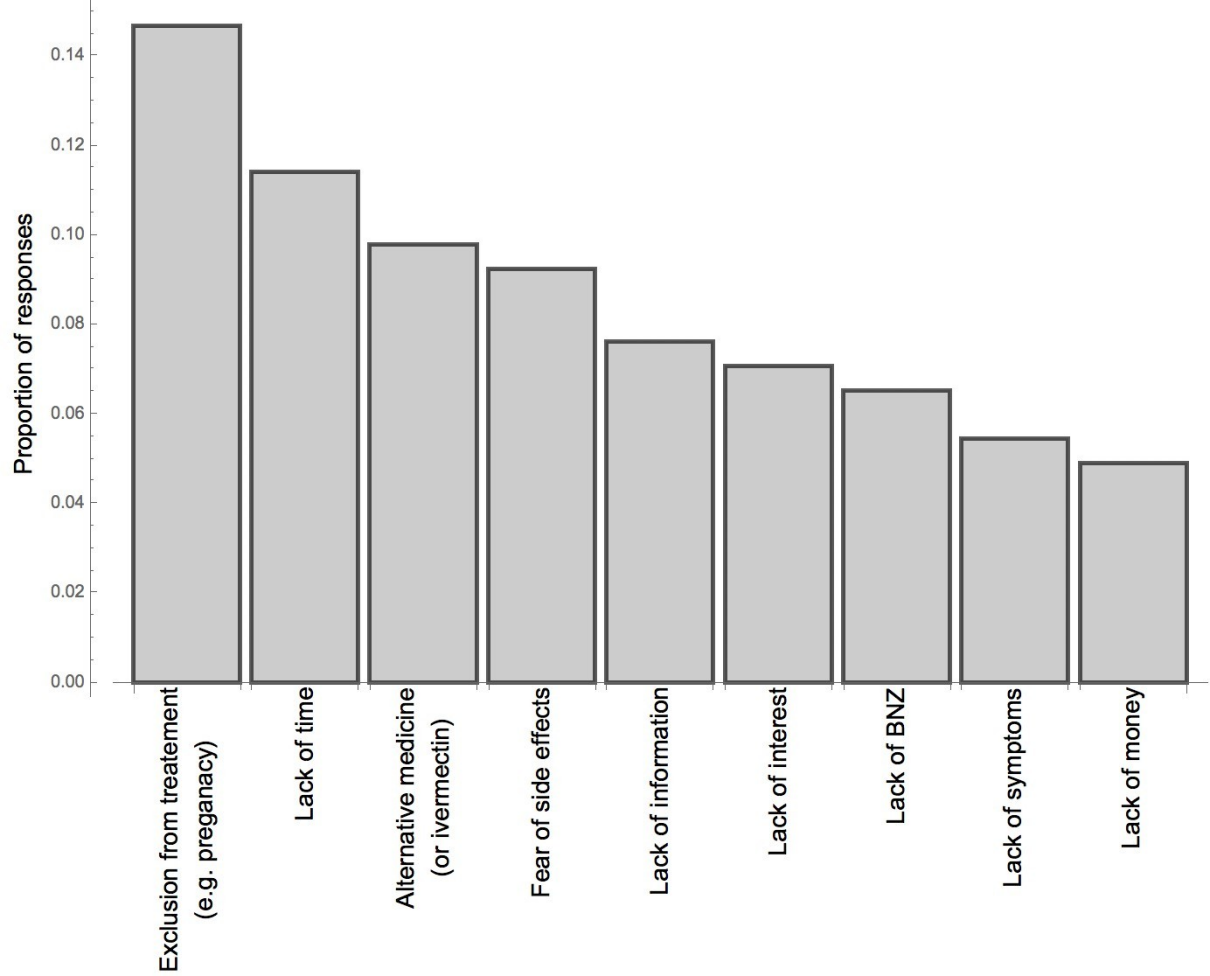
Reasons for not taking BNZ treatment. In this figure, the responses of participants, who had not been treated with BNZ, to the question “What was your reason for not taking treatment?” are given.

Most participants (76.4%, 511/669) stated that they had been tested for CD and 46.2% (236/511) of these reported a positive test (Fig 6). CD testing seemed to be offered with a low barrier to access at the HCs, during campaigns and was often recommended by healthcare professionals during patient care (V28-30/S2 Table). Many participants performed a diagnostic test “just to know” (V30/S2 Table). A total of 23.6% (158/669) never took a test for CD although 29.7% (47/158) of these perceived that the possibility of them being infected was high/very high. Although 43.2% (102/236) stated that they had been treated for CD, only 22.0% (52/236) referred to BNZ and only 80.8% (42/52)—therefore 17.8% (42/236) of all positively tested- completed the standard treatment course of BNZ (Fig 6). Alternative treatment options are offered by a wide range of stakeholders, including government and private healthcare professionals, sometimes with very high prices (V32-34/S2 Table). Ivermectin plays an important role in its veterinary injectable formulation, which is consumed orally and even prescribed by some healthcare professionals (V12, V33/S2 Table; Fig 6). Reasons for not getting treated with BNZ were diverse and are given in Fig 7. Most patients started BNZ treatment within a year of diagnosis (Fig 6). Convincing successfully treated patients to share their experience within communities was highlighted as a solution to increasing treatment compliance due to the high value attributed to other people as information sources (V35/S2 Table).

#### Tertiary prevention—care of chronic CD morbidity

A total of 64.0% (151/236) of those with positive test results, 20.2% (50/248) of those with negative test results and 30.7% (47/153) without CD tests stated at least one chronic symptom compatible with cardiac and/or gastrointestinal manifestations of CD. The management of chronic complications was raised as a major issue, ranging from simple symptomatic treatment to the inaccessibility of pacemakers, as well as high direct and indirect costs to be paid out of pocket for those without formal health insurance (65.3%, 436/669; V36-38/S2 Table). On the other hand, stigma and distrust of pacemakers could lead to people opting against its implementation despite free campaigns (V39-40/S2 Table).

#### Associations of socioeconomic status (SES) on responses

**Fig 8:**
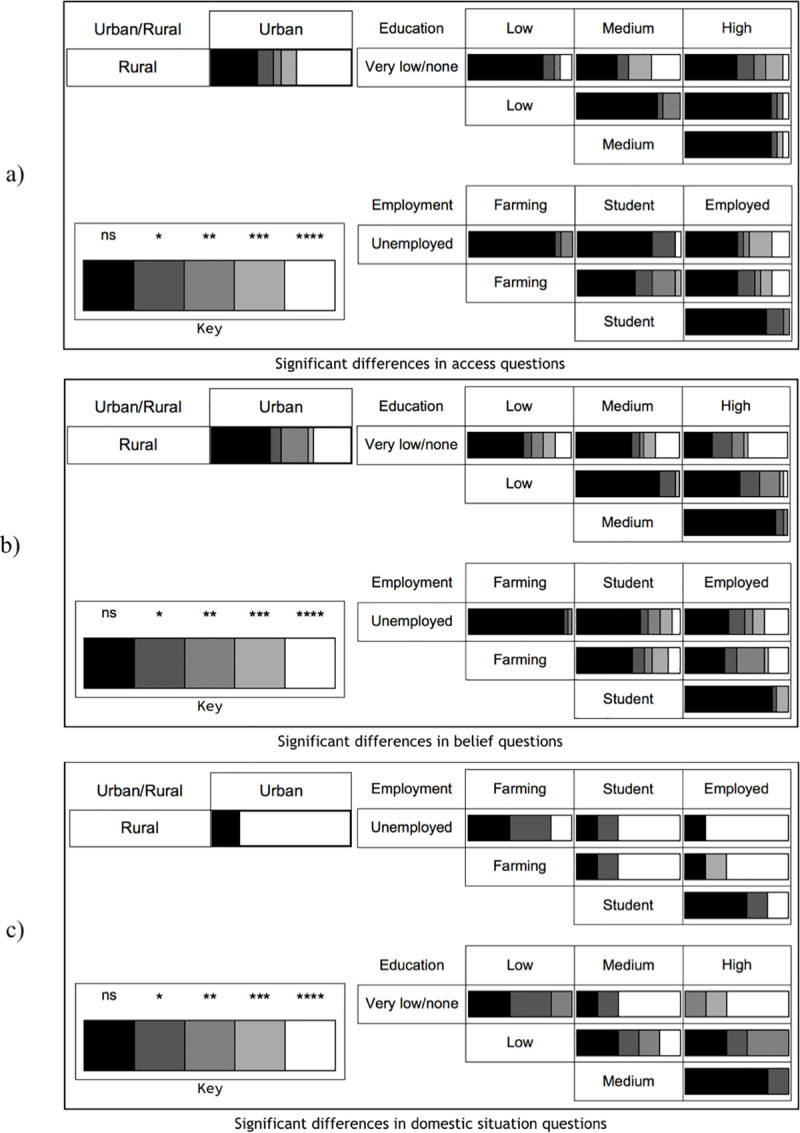
The number of questions for which there were different levels of significance between the SES classes. Horizontal length of shaded block corresponds to the number of questions of that significance level between the particular classes: a) Questions related to access (n = 19), b) to beliefs (n = 25), and c) to the domestic situation (n = 5). For more detailed analyses as well as questions please see S1 Fig.

Three socioeconomic factors were tested for their influences on responses to the quantitative interview questions: Employment, education and living in urban/rural areas. For several responses, significant associations with SES could be observed (S1 Fig; Fig 8A–8C).

#### Healthcare system barriers and social determinants of CD

Several barriers were identified and traditional medicine was often considered to be more affordable, accessible and acceptable within the cultural context (V42/S2 Table). Extreme poverty was associated with CD and a lack of mobility of the rural population, hindering access to information and healthcare. Poverty was connected with the prioritization of the most urgent matters (work and food) and with becoming an easy target for irresponsible private practitioners advertising wonder drugs (V43-44/S2 Table). Political determinants included the need to prioritize CD as a root problem of society, to assure financial sustainability (V45/S2 Table) and the perceived inequitable distribution of benefits (V46-47/S2 Table). Interestingly, stigma and discrimination often only came up when specifically asking about it. CD was mostly considered normal within communities (V48/S2 Table) and the common practice of using negative CD test results as requirements for jobs and bank loans was considered discriminative by some, but was understandable by other participants (V49-51/S2 Table).

#### Lessons learned and community priorities for future projects

A total of 32.9% (220/669) of participants had heard about and 14.3% (96/669) had participated in MSF activities. Positive aspects mentioned were raised awareness, improved and self-sustained primary prevention within some communities as well as increased access to care and capacities among health staff and community volunteers. Critiques included mass diagnosis without ensuring sustained access to treatment and long term support, a situation worsened by BNZ stock-outs during the last two years. No data from MSF were available for comparison. MSF officials stated that the data could not be located and no evaluation had been performed. Participants’ priorities included primary preventive measures, integrated interventions, the inclusion of CD into plans and policies of other sectors (CD mainstreaming) (V57/S2 Table). Priorities also included more effective treatment options, improved access to specialized cares, as well as patients’ desires to take an active role in the control of CD and its social consequences (V56/S2 Table).

## Discussion

This study paints an overall picture of CD in the municipality of Monteagudo through the use of a convergent mixed method approach, representative geographic coverage, and by including a wide range of stakeholders. It was conducted approximately two years after a large healthcare campaign with the aim of decentralizing integrated care including early diagnosis and treatment [10,19].

A high level of general awareness of CD as well as theoretical knowledge on vector transmission and prevention were found. Compared with former studies in Bolivia and neighbouring countries [16,26–28], this indicates a significant change since the NCP was launched in 1999 [16,29].

The self-reported prevalence of vectors in households by 10.8% of all participants at the time of this study (and 27.8% of participants during the last year) is in contrast to official numbers provided by governmental sources (S4 Table, [11]). The discrepancy needs to be discussed and this study indicates that vectors are still routinely found in houses in the study area. Additionally, the wild *Triatoma infestans* population could lead to re-infestation from sylvatic cycles at any time (V23/S2 Table) [30,31]. There is a lack of evidence as well as knowledge regarding the role of oral and animal transmission cycles [32] which is needed to thoroughly understand the local eco-epidemiology, especially in transmission hot spots (self-reported vector infestation rates of up to 30%). Considering the important role of veterinarians as sources of information and treatment, as well as the ongoing practice of living in close proximity to animals, a One Health approach [33] with an integration of CD topics in the veterinary curriculum and joint control strategies could help to reduce infestation rates, in order to create treatment preconditions for all.

PMT elements indicate that despite the high perceived disease severity, a decreasing perceived vulnerability for (re-) infection could lead to a reduction in threat appraisal and a false sense of security. Therefore, sustained vector control and participative surveillance with an active community and adequate resources might be more strategically important than seeking elimination, and information campaigns should highlight the ongoing risk for reinfection and reinfestation [2,34]. The asymptomatic course of CD (especially during its acute phase, being rarely diagnosed at this stage) and the fact that only up to 30% of infected individuals develop organ damage [1], could also cause individuals who see others not affected to think that they are invulnerable.

Diagnostic coverage, with 76.4% of study participants stating that they had been tested for CD, was surprisingly high compared to estimated screening coverage in other endemic settings [1,35]. In contrast to the often reported resistance to diagnosis (“I didn't want to know” [1,17,36]), many participants were tested “just to know” if they were infected (V30/S2 Table). Diagnosis did however not directly lead to treatment. Although BNZ coverage seemed to have increased (from 10% in 2015 [19]), it still remained low, with only 17.8% of participants with positive CD test results stating that they had completed therapy. Although parasiticidal treatment for adults in the chronic phase of CD remains controversial [1,2,37], it has proven effective in interrupting vertical transmission when administered to women of childbearing age [38]. This should be highlighted, as congenital transmission is acquiring a higher relevance in endemic countries, due to the efficacy of the vector control programs [39] and the preventable character of vertical CD. Within the coping appraisal pathway, insecurities about the effectiveness of treatment linked to an unclear understanding of serologic findings and a high perception of costs were identified as main inhibitors for secondary preventive behaviour. This cost-benefit analysis made people at times prefer alternative medicines, considered as equivalent, and reflecting Bolivia’s system of medical pluralism [40]. Interestingly, a vibrant market for veterinary ivermectin against CD was identified, with several animal and human healthcare professionals involved in its distribution, despite the lack of scientific evidence. Of all participants with a positive CD test result, 28.4% stated being treated with veterinary ivermectin, a dimension that to our knowledge has not been described before. Additionally, there appears to be a statistically significant relationship between reporting treatment with veterinary ivermectin and education level. There are other common practices that lack sufficient scientific evidence, such as locally recommended diet schemes during treatment [41].

A lack of knowledge of clinical manifestations and a long indeterminate phase before possible progression might also hamper early treatment seeking behaviour [17,42,43] and has been described as a factor for underdiagnosis when infected individuals move to non-endemic cities or countries [17,44–46]. Of great concern is the sparse awareness of treatment efficacy within newborn and children as well as in women of fertile age [38]. Treatment in young age groups is associated with high cure rates and fewer adverse events and nearly 100% effective in newborns [47]. These aspects should routinely be highlighted during community-based campaigns and understandable models should be created to explain serologic test results (that are not linked to disease progression or organ involvement [1]). Healthcare staff should be trained to homogeneously communicate current knowledge on CD progression [48]. The creation of explanatory models should include all relevant informants and misconceptions.

Our study highlights the importance attributed by the community to tackling social determinants of CD. Although CD could be found among all types of participants, the resulting burden was clearly related to extreme poverty and lack of education, resulting in barriers to healthcare access, delayed treatment seeking behaviour and the acceptance of discriminative practices such as the exclusion from formal jobs or bank loan access. The low health insurance coverage (34.7%) contributes to a vicious cycle of CD and poverty that needs to be tackled at a national level, e.g. by the accessible provision of early diagnosis (including ECGs) and symptomatic care for chronic morbidity within universal healthcare coverage packages [49]. A new Bolivian Universal Health Coverage strategy was implemented in 2019 [50], after our study was concluded. Its impact on integrated care and CD related burdens should be assessed soon. Additionally, we strongly believe that legislation should be reviewed to avoid structural exclusion of affected populations in order to tackle CD and other NTDs [51].

Sustained availability of adequate antitrypanosomal drugs, increased resources for vector surveillance and control, accurate epidemiological data of different sources and accessibility to these for all stakeholders involved in CD control are likely to increase accountability, local ownership, and community participation. Epidemiological data should be collected in a way which avoids the vicious data cycle [52]. Interestingly, although CD has a great impact on the lives of the Bolivian population, it seems to be of very low visibility within national health statistics [53]. Therefore, better indicators of chronic morbidity and the influence of CD on other development markers such as maternal mortality and regional socio-economic development (V45/S2 Table) could help to monitor the real impact of CD interventions and to gain international support [54,55]. NTDs have been described as an important indicator with which to measure successful implementation of the Sustainable Development Goals, since they track whether there is no one left behind [56]. Overall, CD shows complex interactions and dependencies: It is not possible to treat with infestation rates >3% and the lack of perceived effective antiparasitic and symptomatic treatment options undermines the motivation for primary and secondary prevention. Holistic approaches against NTDs [57] and community/patient empowerment [58,59] could help to overcome those structural barriers.

This study had limitations: Due to dangers after nightfall and the lack of a communication network in case of emergencies, the sampling procedure was constrained. Females were overrepresented in quantitative interviews as more women were encountered when approaching households during the daytime. Furthermore, local informants indicating a longer than one-hour walking distance from the last accessible path led to the exclusion of the respective households. Another limitation was the inaccessibility of detailed data on MSF interventions and statistics.

Summing up, great differences were found amongst preventive actions: Knowledge on primary prevention and early diagnosis often led to preventive actions and some communities even sought self-organized solutions to assure sustainability. Knowledge gaps, insecurities about early treatment efficacy and the high perceived costs made patients often opt for alternative treatments. More long-term studies on disease progression, coinfections and BNZ efficacy in asymptomatic and early chronic phases, as well as the development of biomarkers [60] for cure/disease progression could help to generate evidence for healthcare personnel, whose insecurities on treatment effectiveness and on how to explain sustained positive serologic results also reflected in patients’ decisions. More effective use of better data including data sharing, adapted capacity building for healthcare staff, key stakeholders and communities could help to convey these messages in a locally understandable way. Large health interventions should be accompanied by operational, community-based research and evaluation. They should generate accessible as well as transparent data in order to increase local ownership and enable future projects to build on lessons learned, such as the importance of monitoring misunderstanding when introducing new therapeutic schemes and diagnostic tools. Integrated interventions tackling all phases of prevention up to accessible, specialized care for chronic complications, social determinants and consequences of CD, as well as narratives from successfully treated patients were proposed as solutions. Community engagement and the integration of all stakeholders in the pluralistic medical system of Bolivia are important factors which must be taken into account to ensure sustainability and advocacy for CD patient’s rights.

## Supporting information

S1 Text Survey argument and questionnaire. (DOCX)

S2 Text Initial guidelines for qualitative interviews. (DOCX)

S1 Table Description of included communities. (DOCX)

S2 Table Verbatims of study participants. (DOCX)

S3 Table Information sources and level of knowledge on CD. (DOCX)

S4 Table Primary preventive practices of study participants against CD (re-)infection. (DOCX)

S1 Fig Effect of socioeconomic factors on the answers to questions by education level, employment and rural/urban dwelling. (DOCX)
