# Supplementary material for: “We have already heard that the treatment doesn't do anything, so why should we take it?”: A mixed method perspective on Chagas disease knowledge, attitudes, prevention, and treatment behaviour in the Bolivian Chaco
Source: PLoS Negl Trop Dis. 2020 Oct 29;14(10):e0008752. doi: 10.1371/journal.pntd.0008752 (PMC7595318; doi:10.1371/journal.pntd.0008752)
Supplement: S1 Text — (DOCX) [file pntd.0008752.s001.docx]

**S1 Text. Survey argument and questionnaire.** English translation as well as original Spanish language version of the survey questionnaire used in this study.

**A. Knowledge**

- Knowledge on Transmission
- Clinical Knowledge
- Knowledge on Prevention (Primary and Secondary/Tertiary Prevention)

**B. Attitudes (aligned to Protection Motivation Theory Framework)**

Assessed on a Likert Scale (5 points: strongly agree, agree, uncertain, disagree, strongly disagree)

**Threat appraisal**

**Perceived Severity (multidimensional)**

- Risk of Death
- Chronic Heart/ Gastrointestinal Manifestation
- Rate of onset (sudden/slow), imminent vs delayed consequences.
- Psychosocial Dimension (interference with social role)
  - Problems finding a job
  - Stigma
  - Problems to care for family
- Socioeconomic consequences

**Perceived Vulnerability**

- Of currently being infected
- Of getting infected due to current housing/ animal conditions
- Of developing complications (+/- dying)

**Fear arousal**

The idea of being infected makes me feel (not anxious – very anxious)

**Coping appraisal**

**Response effectiveness**

Perceived effectiveness of vector control measures

- Remodeling houses
- Hygiene practices/ keeping animals out of house
- Informing PIV/ National Program
- *Vinchucas*

Perceived effectiveness of early diagnosis and treatment

- Mother to Child Transmission
- Children
- Effectiveness of seeking early diagnosis (incl. willingness/knowledge of health staff, Health Access Barriers)
- Perceived effectiveness of treatment with Benznidazol/ Nifurtimox

**Self- efficacy**

- Able to prevent (re-) infection
- Able to undertake necessary steps get PIVs/ National Program to spray my house in case of reinfestation
- Able to obtain early diagnosis
- Able to obtain treatment
- Able to complete treatment

**Response Cost**

- Costs for diagnosis
- Costs for treatment
- Adverse drug events
- Indirect costs (transport, additional tests, missed days of work)
- Costs for remodeling house
- Having to live with the knowledge of CD infection
- Job discrimination

**C. Current behavior/ Practices**

- Primary Prevention: Current vectors in house, applied methods of transmission prevention
- Secondary/ Tertiary Prevention: Previous diagnosis, previous treatment, stage of disease, early diagnosis, early treatment (or reasons for delay), Time from onset of symptoms to diagnosis, Time from diagnosis to starting of treatment.
- Influence of former campaigns on current practices

**SOCIODEMOGRAPHIC DATA**

| Number of Interview |  | Code |  | |
| --- | --- | --- | --- | --- |
| Date (dd/mm/yy) |  | Interviewer |  | |
| Community |  | Health Center |  | |
| GPS Community |  | GPS Health Center |  | |
| Age (years) |  | Gender | Male | Female |
| Identification/ Ethnicity | Chaqueño  Campesino  Guaraní  Quechua  Otros: | Do you have health insurance?  Which one? | Yes  CNS  Ley 475  Other: | No |
| Language mostly spoken |  | Do you have children? | YES | NO |
| Marital Status | Married/ stable relationship | Single | Divorced | Widowed |
| Educational level | None | Primary Incomplete | Primary completed |  |
|  | Secondary school incomplete | Secondary school completed | University/ Technical studies |  |
| Profession |  | | | |
| How many people are living in your household? | | | | |

| **Knowledge** | | | | | |
| --- | --- | --- | --- | --- | --- |
| 1 | Have you ever heard of Chagas disease? | Yes | No | Don´t know |  |
| 2 | As how high would you describe your level of knowledge on Chagas? | None | Low | Medium | High |
| 3 | Where did you get information on Chagas?  *Read options aloud. Multiple choices possible* | Family | At school | Social Media | Radio/ TV |
|  |  | Experience of someone close | Health Center | Health campaigns | Hospital |
| 4 | How is Chagas transmitted?  *Read options aloud. Multiple choices possible* | Vinchucas | Sexually | Blood / organ | Food contaminated by triatome feces |
|  |  | Witchcraft | Mother to child | Contact with an infected person | Other:  Don´t know |
| 5 | How can you prevent getting sick of Chagas Disease?  (Disease and complications)  *Read options aloud. Multiple choices possible* | Improvement of housing conditions | Cleaning/ Hygiene of house | Honey / Propolis | Pregnancy screening |
|  |  | Cumanda beans | Ivermectin | Chemical vector control | Animals/ chicken corrals far from household |
|  |  | Early diagnosis | Early treatment | Other: | Don´t know |
| ***Open answer questions: don´t read options*** | | | | | |
| 6 | What symptoms are produced by acute Chagas?  (at the beginning/ after infection) | Prolonged Fever | Chagoma | Romaña sign | Others |
| 7 | What is the maximum time between infection and developing chronic complications? (How long can the disease be sleeping?) | Days/Weeks | Months | Years  How many? | Don´t know |
| 8 | What symptoms are produced by chronic Chagas disease?  (Chagas of many years) | Obstipation | Syncope | Breathlessness | Swelling of legs |
|  |  | Irregular heart beat | Tiredness | Brady-/Tachycardia | Angina pectoris |
|  |  | Dysphagia | Don´t know | Other: |  |

**KNOWLEDGE**

**INTENTION OF PROTECTIVE BEHAVIOR**

*(Highlight that it is important to give an honest answer)*

| **Intention of protective behavior** | **Strongly disagree** | **Disagree** | **Uncertain** | **Agree** | **Strongly agree** |
| --- | --- | --- | --- | --- | --- |
| I intend to get tested for CD within the next 2 months | 1 | 2 | 3 | 4 | 5 |
| If the test is positive, I will start treatment within maximum 1 month | 1 | 2 | 3 | 4 | 5 |
| I intend to improve my housing conditions to prevent re-infection within the next 2 months | 1 | 2 | 3 | 4 | 5 |

**ATTITUDES**

| **Perception of Severity** | | **Strongly disagree** | **Disagree** | **Uncertain** | **Agree** | **Strongly agree** |
| --- | --- | --- | --- | --- | --- | --- |
| 9 | Chagas is a severe disease | 1 | 2 | 3 | 4 | 5 |
| 10 | People infected with CD have a high risk of developing heart complications | 1 | 2 | 3 | 4 | 5 |
| 11 | People infected have a high risk of dying | 1 | 2 | 3 | 4 | 5 |
| 12 | CD leads to sudden, unprecedented severe complications | 1 | 2 | 3 | 4 | 5 |
| 13 | A person with CD will have problems to care for his/her children | 1 | 2 | 3 | 4 | 5 |
| 14 | A person with CD will have problems to find/ keep a job | 1 | 2 | 3 | 4 | 5 |
| **Perception of Vulnerability** | | **Strongly disagree** | **Disagree** | **Uncertain** | **Agree** | **Strongly agree** |
| 15 | I believe that I have a high risk of currently being infected with CD | 1 | 2 | 3 | 4 | 5 |
| 16 | I believe that our house is likely to be (re)infested with vinchucas | 1 | 2 | 3 | 4 | 5 |
| 17 | I believe that it is likely that I will develop severe complications of CD during my lifetime | 1 | 2 | 3 | 4 | 5 |
| **Fear Arousal** | | | | | | |
| 18 | The idea of being infected makes me feel | Identified (*Chaqueño* culture) | Relaxed | Calm | Preoccupied | Anxious |
| **Response effectiveness- perceived effectiveness of preventive measures** | | **Strongly disagree** | **Disagree** | **Uncertain** | **Agree** | **Strongly agree** |
| 19 | To improve and maintain housing conditions is effective against vinchucas | 1 | 2 | 3 | 4 | 5 |
| 20 | Informing the PIV/ National program about the presence of vinchucas leads to a fast removal | 1 | 2 | 3 | 4 | 5 |
| 21 | Testing pregnant women and treating newborns will heal the baby completely | 1 | 2 | 3 | 4 | 5 |
| 22 | Benznidazol treatment heals children and prevents them from complications | 1 | 2 | 3 | 4 | 5 |
| 23 | Diagnosing Chagas before the onset of symptoms effectively increases the chances to get healed | 1 | 2 | 3 | 4 | 5 |
| 24 | I would advise a friend to get tested, even if he does not have any symptoms | 1 | 2 | 3 | 4 | 5 |
| 25 | If I go to the health center, I will get all the help I need to get cured | 1 | 2 | 3 | 4 | 5 |
| 26 | What could be reasons why a person would not get all the help he/she needs? | High cost | Distance | Can´t miss days of work | No health insurance | No trust in treatment |
|  |  | They don’t treat well | They don’t have knowledge | Stock out of tests | They don´t have medicine | Other: |
| **Perceived Self-Efficacy in Implementing Preventive Measures** | | **Strongly disagree** | **Disagree** | **Uncertain** | **Agree** | **Strongly agree** |
| 27 | I am able to make and maintain improvements to my house for the next 10 years | 1 | 2 | 3 | 4 | 5 |
| 28 | I am able to prevent vector re-infestation during the next 10 years | 1 | 2 | 3 | 4 | 5 |
| 29 | I am able to keep animals far from the house for the next 10 years | 1 | 2 | 3 | 4 | 5 |
| 30 | I am able to prevent my family to get infected by CD in the next 10 years | 1 | 2 | 3 | 4 | 5 |
| 31 | I am able to obtain early diagnosis and treatment if my family gets infected | 1 | 2 | 3 | 4 | 5 |
| **Perceived Response Cost of Implementing Preventive Measures** | | **Strongly disagree** | **Disagree** | **Uncertain** | **Agree** | **Strongly agree** |
| 32 | The costs for remodeling and maintaining good housing conditions would bring financial hardship to my family | 1 | 2 | 3 | 4 | 5 |
| 33 | It is hard to find the time to restructure and maintain the house | 1 | 2 | 3 | 4 | 5 |
| 34 | It´s risky to keep the animals far from the house | 1 | 2 | 3 | 4 | 5 |
| 35 | If I get tested with CD I might lose my job/ will have problems finding a job | 1 | 2 | 3 | 4 | 5 |
| 36 | The treatment has strong negative effects | 1 | 2 | 3 | 4 | 5 |
| 37 | It’s difficult to find the time to get treated | 1 | 2 | 3 | 4 | 5 |
| **Practices** | | | | | | |
| 38 | Do you currently have vinchucas at your house? | Yes | No | Don´t know |  | |
| 39 | Did you find a vinchuca at your house during the last year? | Yes | No | Don´t know |  |  |
| 40 | Do you have animals in / near your house *(< 50 m)*? | Yes  Which one? | No | Don´t know |  |  |
| 42 | What would you do if you find a vinchuca? |  | | | | |
| 41 | Did you ever inform the National Program / Health Center because of the presence of vinchucas? | Yes | No | Don´t know | What happened after reporting the vinchuca? | |
| 42 | Do you perform preventive measures against vinchucas? | Yes | No | Don´t know | Which ones? | |
| 43 | Did a child of yours ever experience any of the following symptoms? | Prolonged fever | Chagoma | Romaña sign | When? | Was he/she treated?  With what? |
| 44 | How is your physical health? | Very good | Good | Fair | Bad | Very bad |
| 45 | How is your psychological and emotional health? | Very good | Good | Fair | Bad | Very bad |
| 46 | In the past 12 months, have you faced problems in accessing healthcare or from healthcare services? | Did not try to access healthcare services | No difficulties | There were difficulties (please describe) | | |
| 47 | Do you **regularly** have any of the following symptoms? | Arrhythmias | Syncope | Breathlessness | Swelling of legs | Brady/ Tachycardia |
|  |  | Obstipation | Dysphagia | Stomach ache | Since when? | |
| 48 | Did you ever get tested for CD? | yes | no | Don´t know | When? | Where? |
| ***Only to answer, if the participant got tested for CD*** | | | | | | |
| Test result | | Positive | Negative | Don´t know | Don´t want to answer | |
| How many tests for CD did you do? | | When was the first test?  **When was the first positive test?**  When was the last test? | | | | |
| Why did you get tested for CD? | | Pregnancy | Symptoms | Death/ Disease of someone | Recommend. by physician | Other: |
|  |  | Because of a project/ campaign | Which one? | | | |
| ***Only to answer, if the test was positive*** | | | | | | |
| When you got tested, did you have any of the symptoms mentioned above? | | Yes | No | Don´t know | Which one?  For how long? (in months) | |
| Did you get treated? | | If yes, with what?  *Multiple answers possible* |  | | | |
|  |  | If no, why not? |  | | | |
| Did you finish the treatment course? | | Yes | No | Why not? | | |
| How much time did pass between the diagnosis and starting treatment? (in months) | |  | Reasons for treatment delay (>1 month): | | | |
| ***Only to answer, if never got tested for CD*** | | | | | | |
| Why did you never get tested for CD? | |  | | | | |
| How do you rate the probability of being infected | | Impossible/ Cero | Low | Medium/ Don´t know | High | Very high |
| ***Everyone has to answer*** | | | | | | |
| 49 | Did you consider getting tested (again)? | Yes | No | Don´t know |  | |
| 50 | Do you believe that the serology (Chagas test) shows how advanced / severe your CD is? | Yes | No | Don´t know |  | |
| 51 | Did you ever take *Ivomec* against Chagas disease? | Yes | No | Which one: | Human form | Veterinary Form |
| 52 | Have you heard about the CD project from MSF? | Yes | No | Don´t know | When? | Where? |
| ***Only to answer, if heard about MSF*** | | | | | | |
| Did you participate in any activity organized by MSF? Which one? | | Educational meetings | Community gathering | Diagnostic activities | Other: | |
| Did anything change after the MSF project? What? | |  | | | | |
| Has your behavior been influenced by the project of MSF? How? | |  | | | | |

| Número de encuesta |  | Código (*p. ej. LP5.2)* |  | |
| --- | --- | --- | --- | --- |
| Fecha (dd/mm/aa) |  | Entrevistador |  | |
| Comunidad |  | Centro de Salud |  | |
| GPS Comunidad |  | GPS Centro de Salud |  | |
| Edad (años) |  | Sexo | Masculino | Femenino |
| ¿Con que grupo étnico se identifica? | Chaqueño  Campesino  Guaraní  Quechua  Otros: | ¿Tiene seguro  de salud?  ¿Cuál? | Sí  Cuál:  CNS  Ley 475  Otras: | No |
| Idioma más hablado |  | ¿Tiene hijos? | Sí | No |
| Estado civil | Casado / unión estable | Soltero | Divorciado | Viudo |
| Nivel de escolaridad | Ninguno | Primaria incompleta | Primaria completa |  |
|  | Secundaria incompleta | Secundaria completa | Técnico/superior | Académico |
| Profesión |  | | | |
| ¿Cuántas personas conforman su hogar? | | | | |

**DATOS SOCIODEMOGRÁFICOS**

**CONOCIMIENTOS**

| **Conocimientos** | | | | | |
| --- | --- | --- | --- | --- | --- |
| 1 | ¿Ha oído hablar sobre la enfermedad de Chagas? | Sí | No | No sé |  |
| 2 | ¿Cuánto sabe de Chagas? | Nada | Poco | Mediano | Mucho |
| 3 | ¿Dónde lo informaron sobre el Chagas?  *Leer opciones, respuesta múltiple* | En familia | En la escuela | En redes sociales | En radio / TV |
|  |  | Experiencia de alguien cercano | Posta / Centro de salud | Campañas de salud | Hospital |
| 4 | ¿Cómo se transmite el Chagas?  *Leer opciones, respuesta múltiple* | Vinchucas | Sexualmente | Sangre / órganos | Alimentos con heces de vinchuca |
|  |  | Hechizo | Madre-hijo | Contacto con una persona infectada | Otros  No sé |
| 5 | ¿Cómo se previene la enfermedad de Chagas?  *Leer opciones, respuesta múltiple* | Mejoramiento de vivienda | Ordenamiento/ limpieza de la vivienda | Miel/ Propóleo | Test durante el embarazo |
|  |  | Cumanda | Ivomec | Control químico de vinchucas | Corrales/ gallineros alejados |
|  |  | Prueba de Chagas oportuna | Tratamiento  oportuno | Otros: | No sé |
| ***Pregunta abierta, no leer respuestas*** | | | | | |
| 6 | ¿Qué molestias produce el Chagas al principio? | Fiebre prolongada | Hinchazón de ganglios cerca de picadura | Hinchazón persistente del ojo | Gastritis |
|  |  | Dolor de espalda | Problemas al orinar | Otros | No sé |
| 7 | ¿Después de cuánto tiempo comienzan las molestias? | Días / Semanas | Meses | Años,  ¿hasta cuántos? | No sé |
| 8 | ¿Qué molestias produce el Chagas avanzado?  (Chagas de muchos años) | Estreñimiento | Desmayos | Falta de aire | Hinchazón de piernas |
|  |  | Corazón late irregular | Cansancio | Corazón muy rápido / lento | Dolor en el pecho |
|  |  | Problemas al tragar | No sé | Otros: |  |

**INTENCIÓN DE COMPORTAMIENTO PREVENTIVO**

*(Resaltar que es importante responder honestamente)*

| **Intención de Comportamiento Preventivo** | **Muy en desacuerdo** | **En desacuerdo** | **Neutral / no sé** | **De acuerdo** | **Muy de acuerdo** |
| --- | --- | --- | --- | --- | --- |
| Tengo la intención de hacerme la prueba en los próximos 2 meses | 1 | 2 | 3 | 4 | 5 |
| Si sale positivo voy a empezar el tratamiento en un plazo máximo de 1 mes | 1 | 2 | 3 | 4 | 5 |
| Tengo la intención de mejorar mi vivienda para prevenir las vinchucas en los próximos 2 meses | 1 | 2 | 3 | 4 | 5 |

**ACTITUDES**

| **Percepción de Severidad** | | **Muy en desacuerdo** | **En desacuerdo** | **Neutral / no sé** | **De acuerdo** | **Muy de acuerdo** |
| --- | --- | --- | --- | --- | --- | --- |
| 9 | Chagas es una enfermedad grave | 1 | 2 | 3 | 4 | 5 |
| 10 | Personas infectadas por Chagas tienen un alto riesgo de problemas de corazón (peligro) | 1 | 2 | 3 | 4 | 5 |
| 11 | Personas infectadas por Chagas tienen un alto riesgo de morir | 1 | 2 | 3 | 4 | 5 |
| 12 | Personas con Chagas pueden morir en cualquier momento | 1 | 2 | 3 | 4 | 5 |
| 13 | Una persona infectada por Chagas tendrá dificultades para cuidar de sus hijos | 1 | 2 | 3 | 4 | 5 |
| 14 | Una persona infectada por Chagas tendrá problemas para encontrar / mantener trabajo | 1 | 2 | 3 | 4 | 5 |
| **Percepción de Vulnerabilidad** | | **Muy en desacuerdo** | **En desacuerdo** | **Neutral / no sé** | **De acuerdo** | **Muy de acuerdo** |
| 15 | Cree que la posibilidad de que usted se infecte (nuevamente) con Chagas es alta | 1 | 2 | 3 | 4 | 5 |
| 16 | Su casa es de fácil acceso para vinchucas (ingreso) | 1 | 2 | 3 | 4 | 5 |
| 17 | Cree que va a tener graves molestias por Chagas durante su vida | 1 | 2 | 3 | 4 | 5 |
| **Temor a la Enfermedad** | | | | | | |
| 18 | La idea de estar infectado/a con Chagas le hace / haría sentir | Identificado | Relajado | Tranquilo | Preocupado | Angustiado |
| **Percepción de Efectividad de las Medidas Preventivas** | | **Muy en desacuerdo** | **En desacuerdo** | **Neutral / no sé** | **De acuerdo** | **Muy de acuerdo** |
| 19 | Tener la casa arreglada y limpia ayuda contra las vinchucas | 1 | 2 | 3 | 4 | 5 |
| 20 | Si denuncia la presencia de vinchucas en su casa, vendrán pronto a rociar | 1 | 2 | 3 | 4 | 5 |
| 21 | Si las embarazadas se hacen la prueba de Chagas y se trata al bebé, el bebé se sanará completamente | 1 | 2 | 3 | 4 | 5 |
| 22 | El tratamiento con benznidazol sana a los niños y previene las complicaciones | 1 | 2 | 3 | 4 | 5 |
| 23 | El diagnóstico y tratamiento temprano puede curarle | 1 | 2 | 3 | 4 | 5 |
| 24 | Recomendaría a sus amigos hacerse la prueba, aunque no tengan síntomas | 1 | 2 | 3 | 4 | 5 |
| 25 | Si va a la posta/centro de salud, fácilmente recibirá toda la ayuda para curarse | 1 | 2 | 3 | 4 | 5 |
| 26 | ¿Qué puede impedir a una persona recibir diagnóstico y/o tratamiento?  *Leer opciones, respuesta múltiple* | Alto coste | Larga distancia | No tienen prueba | No tienen conocimiento | No tienen medicamento |
|  |  | No tratan muy bien a la gente | No puede faltar en trabajo | Por no tener seguro | Por desconfianza al medicamento | Otros: |
| **Percepción de Auto-efectividad de Implementar Actividades Preventivas** | | **Muy en desacuerdo** | **En desacuerdo** | **Neutral / no sé** | **De acuerdo** | **Muy de acuerdo** |
| 27 | Puede mantener su casa en buen estado durante los próximos 10 años | 1 | 2 | 3 | 4 | 5 |
| 28 | Puede evitar la presencia de vinchucas en su casa durante los próximos 10 años | 1 | 2 | 3 | 4 | 5 |
| 29 | Logra mantener gallineros y corrales alejados de su vivienda y en buen estado durante los próximos 10 años | 1 | 2 | 3 | 4 | 5 |
| 30 | Es capaz de prevenir que su familia se infecte con el Chagas | 1 | 2 | 3 | 4 | 5 |
| 31 | Es capaz de hacer lo necesario para que su familia se cure si se enferma de Chagas | 1 | 2 | 3 | 4 | 5 |
| **Percepción del Coste de Implementar Comportamiento Preventivo** | | **Muy en desacuerdo** | **En desacuerdo** | **Neutral / no sé** | **De acuerdo** | **Muy de acuerdo** |
| 32 | Los gastos para arreglar su casa traerían problemas financieros para su familia | 1 | 2 | 3 | 4 | 5 |
| 33 | Es difícil encontrar tiempo para mejorar y mantener su vivienda | 1 | 2 | 3 | 4 | 5 |
| 34 | Es arriesgado dejar los animales alejados de la casa | 1 | 2 | 3 | 4 | 5 |
| 35 | Si le diagnostican Chagas puede perder / no encontrar trabajo | 1 | 2 | 3 | 4 | 5 |
| 36 | Los medicamentos tienen complicaciones graves | 1 | 2 | 3 | 4 | 5 |
| 37 | Es difícil encontrar el tiempo para hacer el tratamiento | 1 | 2 | 3 | 4 | 5 |
| **Prácticas** | | | | | | |
| 38 | ¿Tiene vinchucas en la casa actualmente? | Sí | No | No sé |  | |
| 39 | ¿Ha tenido vinchucas en la casa en el último año? | Sí | No | No sé |  |  |
| 40 | ¿Tiene animales dentro/ cerca de la casa?  *Definición de cerca es < 50 metros* | Sí, ¿cuáles? | No | No sé |  | |
| 42 | ¿Qué haría si encuentra una vinchuca? |  | | | | |
| 41 | ¿Alguna vez ha denunciado la presencia de vinchucas a los PIV / al servicio de salud? | Sí | No | No sé | ¿Qué pasó después de denunciar? | |
| 42 | ¿Hace algo para prevenir las vinchucas? | Sí | No | No sé | ¿Qué? | |
| 43 | ¿Algún hijo suyo ha tenido alguna vez algunas de las siguientes molestias? | Fiebre prolongada | Chagoma  (inflamación de ganglios en zona de picadura) | Signo de Romaña (hinchazón persistente del ojo) | ¿Cuándo? | ¿Se trató?  ¿Con qué/quién? |
| 44 | ¿Cómo se encuentra usted de salud? | Muy bien | Bien | Regular | Mal | Muy mal |
| 45 | ¿Cómo se siente emocionalmente? | Muy bien | Bien | Regular | Mal | Muy mal |
| 46 | En los últimos años, ¿ha tenido problemas en acceder a los servicios de salud? | No intentó acceder a servicios de salud | Sin dificultades | Hubo problemas. Por favor, explique: | | |
| 47 | ¿Siente usted **habitualmente** algunas de las siguientes molestias? | Dolor en el pecho | Desmayos | Falta de aire | Hinchazón de piernas | Corazón muy rápido / lento |
|  |  | Estreñimiento | Problemas al tragar | Dolor de estómago | ¿Desde cuándo? | |
| 48 | ¿Se ha hecho una prueba de Chagas? | Sí | No | No sé | ¿Dónde? | |
| ***Solo responder si se hizo el test*** | | | | | | |
| ¿Cuál fue el resultado? | | Positivo | Negativo | No sé | No quiero responder | |
| ¿Cuántos test de Chagas se ha hecho? | | ¿Cuándo fue el primer test?  **¿Cuándo fue el primer test positivo?**  ¿Cuándo fue el último test? | | | | |
| ¿Por qué se hizo la prueba de Chagas? | | Embarazada | Molestias | Muerte / Enfermedad de alguien | El médico lo recomendó | Otros: |
|  |  | Por una campaña/ un proyecto | ¿Cuál? | | | |
| ***Solo responder si tuvo un test positivo*** | | | | | | |
| Cuando se hizo la prueba de Chagas, ¿tenía las molestias (mencionadas arriba)? | | Sí | No | No sé | ¿Cuáles?  ¿Durante cuánto tiempo? (meses) | |
| ¿Se ha tratado? | | Sí, ¿con qué?  *Múltiples respuestas posibles* |  | | | |
|  |  | No, ¿por qué no? |  | | | |
| ¿Ha completado el tratamiento? | | Sí | No | ¿Por qué no? | | |
| ¿Cuánto tiempo pasó entre el diagnóstico y el tratamiento? (meses) | |  | Motivos de posible retraso de tratamiento (>1 mes): | | | |
| ***Solo responder si nunca se hizo un test*** | | | | | | |
| ¿Por qué nunca se hizo una prueba de Chagas? | |  | | | | |
| ¿Cómo estima la probabilidad de que esté infectado/a? | | Imposible / Cero | Baja | Mediana /  No sé | Alta | Muy alta |
| ***Todos tienen que responder*** | | | | | | |
| 49 | ¿Ha considerado hacerse (nuevamente) la prueba? | Sí | No | No sé |  | |
| 50 | ¿Cree que el laboratorio le indica la gravedad de la enfermedad de Chagas? | Sí | No | No sé |  |  |
| 51 | ¿Alguna vez ha tomado Ivomec contra el Chagas? | Sí | No | Cuál: | Humano | Veterinario |
| 52 | ¿Ha escuchado del proyecto de Chagas de Médicos Sin Fronteras (MSF)? | Sí | No | No sé | ¿Cuándo? | ¿Dónde? |
| ***Solo responder si supo de MSF*** | | | | | | |
| ¿Ha participado en una actividad de MSF? ¿En cuál? | | Charlas educativas | Encuentros comunitarios | Actividad de diagnóstico | Otros: | |
| ¿Ha cambiado algo después del proyecto de MSF? ¿Qué? | |  | | | | |
| ¿El proyecto de MSF influyó en su comportamiento? ¿Cómo? | |  | | | | |
