# Supplementary material for: “We have already heard that the treatment doesn't do anything, so why should we take it?”: A mixed method perspective on Chagas disease knowledge, attitudes, prevention, and treatment behaviour in the Bolivian Chaco
Source: PLoS Negl Trop Dis. 2020 Oct 29;14(10):e0008752. doi: 10.1371/journal.pntd.0008752 (PMC7595318; doi:10.1371/journal.pntd.0008752)
Supplement: S2 Text — (DOCX) [file pntd.0008752.s002.docx]

**S2 Text. Initial guidelines for qualitative interviews.** Guidelines were adapted and focused throughout the study. Below the English translation as well as the Spanish original version can be found.

**INTERVIEW GUIDELINE FOR PATIENTS AND RELATIVES**

| **Themes** | **Main questions** | **Follow up questions** |
| --- | --- | --- |
| **Experience with CD** | Please cite the first 3 words that come into your mind when you hear ‘Chagas’ |  |
|  | Please tell me your story about Chagas | How did you know you got infected?  What was your first thought/reaction when you knew you had Chagas disease?  What physical symptoms did you have?  What did you do when you started to have symptoms/ were diagnosed Chagas?  Did you take any kind of remedies?  Who told you about the diagnosis? |
| **Experience seeking diagnosis** | Can you tell me about your experience seeking diagnosis of Chagas? | Who did you consult?  Did you have to travel somewhere?  How was your experience with the health center staff?  How long did it take between the first symptoms and the Chagas test?  Where were you diagnosed?  What did you do after you were diagnosed with Chagas? |
| **Experience with treatment** | What is your opinion about Chagas treatment? | Have you started treatment? with whom?  How long after the diagnosis did you initiate it?  Postponed treatment for any reason?  Can you tell me how was your experience taking the drugs?  Have you taken it regularly?  Have you had any adverse effects?  Have you completed treatment?  Do you feel the treatment helped you? |
|  | Do you know anything about alternative Chagas treatments? | What are the alternative drugs used against Chagas?  Do you know of any experience (your/or someone else)?  What's your opinion on that? |
| **Health system access barriers**  (5 dimensions of access: accommodation, accessibility, availability affordability, acceptability) | Did you have difficulties getting a diagnosis or treatment?  Which ones? | Were there any financial difficulties? Which ones?  Can you please describe a typical situation?  Did you have to travel long distances?  Do you think the current health system covers your needs and expectations as a patient with Chagas disease?  How was the availability of diagnostic tests and medicines?  What do you think about the knowledge and capacities of health personnel regarding Chagas disease? |
| **Delay of diagnosis and treatment** | Why do you think that many people in Monteagudo have not yet been diagnosed with CD? | What could be the cause of someone not wanting to be tested?  What could be causes of not starting treatment?  Has the problem of Chagas and treatment been promoted?  What could be improved for more people to have access to treatment? |
| **Chronic morbidity** | What does having CD mean to you? | Please describe if there have been changes in your life since the diagnosis?  Do you have symptoms?  How is the topic handled within the family/with friends?  Has anything changed in your work situation?  Does Chagas produce affective consequences? |
| **Coping** | What helps you? What makes you feel better? | What do you do to face difficult times?  Can you tell me a specific experience (from a difficult event)?  What support in the family/friends/at work do you have?  What is your opinion about the association of people affected by Chagas? |
| **Prevention of reinfection** | What steps do you take to prevent reinfection? | Are there vinchucas in your house?  How do you prevent the house from re-infesting with vinchucas?  What do you recommend to your friends as preventive measures?  Do you know if a neighbor's house has vinchucas?  Where would you report in case of detecting vinchucas?  Do you get visits from the Chagas program technicians-what do they do during the visits? |
| **Experience with former projects** | Could you tell me about your experience with former projects against Chagas? | With respect to previous projects, what worked well, what could be improved?  Do you think that after the project something has changed in the community?  Has the community´s/ your behavior changed? |
|  | What should be improved, so that the community can protect itself against Chagas? | What should be done to get the community involved in preventive activities? (1. To avoid vinchucas; 2. For people to be diagnosed and treated in time).  Who should be included in these activities?  How would you define a good health project for your community? |

**INTERVIEW GUIDELINE FOR KEY INFORMANTS**

| **Themes** | **Main questions** | **Follow up questions** |
| --- | --- | --- |
| **Experience with CD** | Please cite the first 3 words that come into your mind when you hear ‘Chagas’ |  |
|  | What importance does CD play in your community? | Please tell me your experience with a typical situation |
| **Diagnosis and treatment of CD** | How is the procedure to obtain the diagnosis and treatment of Chagas disease? | What works well?  What could be improved?  What symptoms should a person have to do the Chagas test?  How long do you think is the average between symptomatology and diagnosis (in acute, chronic stages)?  What can be factors that delay the timely diagnosis? |
|  | What role do alternative treatments play in the municipality? | What are the alternative drugs used against Chagas?  How does the integration of biomedicine and traditional medicine work with respect to Chagas disease?  Which actors play a role in the promotion of different treatments?  Can you describe a typical situation? |
| **Health access barriers** | What problems can a person confront if seeking diagnosis and treatment for CD? | Will there be financial difficulties? Which ones?  How is the availability of tests and medicines in urban and rural areas?  Can there be problems with distances for certain populations?  Does the current system cover the needs and expectations of different populations?  What do you think about the knowledge and capabilities in the health system to diagnose and treat in a timely manner? |
| **Delay of diagnosis and treatment** | Why do you think many people in Monteagudo have not yet been tested for CD? | What could be the cause of someone not wanting to be tested?  What could be causes of not starting/postponing treatment?  What could be improved for more people to have access to treatment? |
| **Social determinants** | What social problems can a Chagas infected person face in Monteagudo? | Are there any working difficulties? Which ones?  Have you met people who were infected by CDs who were dismissed for Chagas? /who were not hired?  Do you know about protection laws and/or activities for Chagas-infected workers?  What works well, which could be improved?  Is there stigma associated with the disease?  What other social problems could there be? |
| **Chagas control** | What do you think about the organization of communities with respect to Chagas? | Were there any interventions on Chagas disease? from whom? With what content?  Has the situation and organization of the community changed after that?  What components does a successful intervention have to cover?  What works well, what could be improved in the interaction of the National Program and the communities? |
| **Coping** | What do people do to feel better? | What support networks are there within the community?  How does the patient association work? |
| **Snowball sampling** | Who else do you think I should interview? | What information will this person give me?  What is his/ her area of expertise? |

**INITIAL GUIDELINE FOR FOCUS GROUP DISCUSSION**

| **Themes** | **Main questions** | **Follow up questions** |
| --- | --- | --- |
| **Health Priorities** | What are the main health issues in your community? |  |
| **Health System**  (5 dimensions of access: accommodation, accessibility, availability affordability, acceptability) | How does the health system work in your community? | Does the current system cover the needs and expectations of the different populations?  Due long distances cause access problems for certain populations?  How is the availability of diagnostic tests and medications?  Are the costs accessible to all?  What do you think about the knowledge and capacities in the health system to diagnose and treat CD in a timely manner? |
|  | How important are alternative treatments in Monteagudo? | How do people decide between alternative/ traditional medicine and biomedical treatment? Which treatment is used for what? |
| **Importance of CD** | What importance does Chagas play in the Community? | Is this a frequently spoken topic?  Is it taught at school?  What is the percentage that you estimate is infected? |
|  | In what situation should people in the community be tested for Chagas? | What parts of the population are vulnerable/affected?  How is the problem of transmission during pregnancy discussed in the community?  How many are estimated to have performed a test, how many have taken biomedical treatment?  Can there be reasons why someone in the community prefers not to take the test? |
|  | What local services are available for CD? | Where does a person have to go if there are no local services? |
|  | What are the main problems caused by CD in your community? | What symptoms do people have?  Does Chagas produce social consequences in your community? -Can you please describe, if you have heard of someone who suffered from consequences?  Do infected people have the same chances of professional development? |
| **Former projects and campaigns** | Can you tell me about experiences in the community with former campaigns/ projects against CD? | Are there campaigns by the health system?  Were there international NGO campaigns?  Concerning previous projects, what has been done well, what could be improved?  What has changed after previous projects?  Were the changes sustainable? |
| **Preventive measures** | What measures does the community take to protect itself from Chagas? | How many houses do you think have vinchucas?  How are the houses being protected against vinchucas?  Is there any kind of community-level coordination to detect and report on the presence of vinchucas? |
|  | What should be improved, so that the community can protect itself against Chagas? | What should be done to get people in the community involved in preventive activities?  How would you define a good health project for your community? |

**GUÍA DE PREGUNTAS PARA PACIENTES Y FAMILIARES**

| **Temas** | **Preguntas principales** | **Preguntas consecutivas** |
| --- | --- | --- |
| **Experiencia con Chagas** | Por favor, diga las primeras tres palabras que le vienen a la mente si escucha ‘Chagas’ |  |
|  | Por favor, cuénteme su historia respecto al Chagas | ¿Cómo supo que se infectó?  ¿Cuál fue su primer pensamiento/ reacción cuando supo que tenía Chagas?  ¿Qué molestias físicas tenía?  ¿Qué hizo usted cuando empezaron las molestias/ le diagnosticaron Chagas?  ¿Tomó algún tipo de remedio?  ¿A quién le contó sobre el diagnóstico? |
| **Experiencia buscando diagnóstico** | ¿Me puede contar su experiencia buscando diagnóstico de Chagas? | ¿A quién consultó?  ¿Tuvo que viajar a alguna parte?  ¿Cómo fue su experiencia con el personal del centro de salud?  ¿Cuánto tiempo pasó entre los primeros síntomas y la prueba de Chagas?  ¿Dónde le diagnosticaron?  ¿Qué hizo después de que le diagnosticaron el Chagas? |
| **Experiencia con tratamiento** | ¿Que opinión tiene sobre el tratamiento de Chagas? | ¿Ha iniciado tratamiento? ¿Con quién?  ¿Cuánto tiempo después del diagnóstico lo inició?  ¿Postergó el tratamiento por algún motivo?  ¿Me puede contar cómo fue su experiencia tomando los medicamentos?  ¿Lo ha tomado con regularidad?  ¿Ha tenido efectos adversos?  ¿Usted ha completado tratamiento?  ¿Siente que el tratamiento le ayudó? |
|  | ¿Sabe algo sobre tratamientos alternativos contra el Chagas? | ¿Cuáles son los medicamentos alternativos que se utilizan contra el Chagas?  ¿Sabe de alguna experiencia (suya/ u otra persona)?  ¿Cuál es su opinión al respecto? |
| **Barreras de acceso al Sistema de Salud**  (5 dimensiones de acceso: adaptación, accesibilidad, disponibilidad, asequibilidad, aceptabilidad) | ¿Tuvo dificultades en el momento de buscar diagnóstico o tratamiento?  ¿Cuáles? | ¿Hubo dificultades financieras? ¿Cuáles?  Por favor, ¿me puede describir una situación típica?  ¿Tuvo que viajar largas distancias?  ¿Opina que el actual sistema de salud cubre sus necesidades y expectativas como paciente con Chagas?  ¿Cómo fue la disponibilidad de pruebas diagnósticas y medicamentos?  ¿Qué opina sobre los conocimientos y capacidades del personal de salud respecto al Chagas? |
| **Retraso del diagnóstico y tratamiento** | ¿Por qué piensa que muchas personas en Monteagudo aún no se han hecho la prueba de Chagas? | ¿Cuáles podrían ser causas por las que alguien no quisiera hacerse la prueba?  ¿Cuáles podrían ser causas de no comenzar tratamiento?  ¿Ha sido informado sobre las formas de tratar el Chagas y su problemática?  ¿Qué se podría mejorar para que más personas tengan acceso al tratamiento? |
| **Morbilidad crónica** | ¿Qué significa tener Chagas para usted? | Por favor, describa si hay cambios en su vida desde el diagnóstico.  ¿Tiene síntomas?  ¿Cómo se maneja el tema dentro de la familia / con amigos?  ¿Ha cambiado algo en su situación laboral?  ¿El Chagas le produce consecuencias afectivas / en su estado de ánimo? |
| **Superación** | ¿Qué le ayuda a usted? / ¿Qué le hace sentir mejor? | ¿Qué hace usted para superar momentos difíciles?  ¿Me puede contar una experiencia concreta (de algún evento difícil)?  ¿Qué apoyo en la familia/ amigos/ en el trabajo tiene?  ¿Cuál es su opinión sobre la asociación de personas afectadas por Chagas? |
| **Prevención de reinfección** | ¿Qué medidas toma usted para prevenir una reinfección? | ¿Hay vinchucas en su casa?  ¿Cómo evita que la casa se re-infeste con vinchucas?  ¿Qué recomienda usted a sus amigos como medidas de prevención?  ¿Sabe si la casa de algún vecino tiene vinchucas?  ¿Dónde hace la denuncia en caso de detectar vinchucas?  ¿Recibe visitas de técnicos del programa de Chagas? ¿Qué hacen ellos durante las visitas? ¿Se prepara antes de las visitas? |
| **Experiencia con proyectos previos** | ¿Me puede contar su experiencia con proyectos previos contra el Chagas? | Con respecto a proyectos anteriores, ¿qué funcionó bien?, ¿qué se podría mejorar? ¿Piensa que después del proyecto ha cambiado algo? ¿Ha cambiado el comportamiento en la comunidad /su comportamiento? |
|  | ¿Qué se debería mejorar, para que la comunidad pueda protegerse contra el Chagas? | ¿Qué se debería hacer para que la comunidad se involucre en actividades preventivas? (1. Para evitar vinchucas; 2. Para que las personas se diagnostiquen y traten a tiempo)  ¿A quién hay que incluir en estas actividades?  ¿Cómo definiría usted un buen proyecto de salud para su comunidad? |

**GUÍA DE PREGUNTAS PARA INFORMANTES CLAVE**

| **Temas** | **Preguntas principales** | **Preguntas consecutivas** |
| --- | --- | --- |
| **Experiencia con Chagas** | Por favor, diga las primeras tres palabras que le vienen a la mente si escucha ‘Chagas’ |  |
|  | ¿Qué importancia piensa usted que tiene el Chagas en la comunidad de Monteagudo? | Por favor, cuénteme sus experiencias o situaciones típicas |
| **Diagnóstico y tratamiento de Chagas** | ¿Cómo es el procedimiento para obtener el diagnóstico y tratamiento de Chagas? | ¿Qué funciona bien?  ¿Qué se podría mejorar?  ¿Qué síntomas debería tener una persona para hacerle la prueba del Chagas?  ¿Cuánto tiempo estima que es normal entre sintomatología y diagnóstico (en agudo, en crónico)?  ¿Cuáles pueden ser factores que retrasan el diagnóstico oportuno? |
|  | ¿Qué papel juegan tratamientos alternativos en el municipio? | ¿Cuáles son los medicamentos alternativos que se utilizan contra el Chagas?  ¿Como funciona la integración de la biomedicina y la medicina tradicional con respecto al Chagas?  ¿Qué actores juegan un papel en la promoción de los diferentes tratamientos?  ¿Puede describir alguna situación típica? |
| **Barreras de acceso al Sistema de Salud** | ¿Con qué dificultades se puede encontrar una persona que esté buscando diagnóstico o tratamiento? | ¿Habrá dificultades financieras? ¿Cuáles?  ¿Cómo es la disponibilidad de test diagnósticos y medicamentos en zonas urbanas y rurales?  ¿Puede haber problemas de distancias para ciertas poblaciones?  ¿El actual sistema cubre las necesidades y expectativas de las diferentes poblaciones?  ¿Qué opina sobre los conocimientos y capacidades en el Sistema de Salud de diagnosticar y tratar de manera oportuna? |
| **Retraso de diagnóstico y tratamiento** | ¿Por qué piensa que muchas personas en Monteagudo aún no se han hecho la prueba de Chagas? | ¿Cuáles podrían ser causas por las que alguien no quisiera hacerse la prueba?  ¿Cuáles podrían ser causas de no comenzar/postergar tratamiento?  ¿Qué se podría mejorar para que más personas tengan acceso al tratamiento? |
| **Determinantes Sociales** | ¿Con qué problemas sociales puede enfrentarse una persona infectada de Chagas en Monteagudo? | ¿Existen dificultades para realizar actividades laborales? ¿Cuáles?  ¿Ha conocido a personas infectadas que fueron despedidas por tener Chagas? / ¿Que no fueron contratados?  ¿Conoce las leyes y/o actividades de protección para trabajadores infectados por Chagas?  ¿Qué funciona bien, qué se podría mejorar?  ¿Hay estigma asociado a la enfermedad?  ¿Qué otros problemas sociales podría haber? |
| **Control de Chagas** | ¿Qué opina sobre la organización de las comunidades con respecto al Chagas? | ¿Hubo intervenciones sobre el Chagas? ¿De quién? ¿Con qué contenidos?  ¿Ha cambiado la situación y la organización de la comunidad después de la campaña?  ¿Qué componentes debe abarcar según usted una intervención exitosa?  ¿Qué funciona bien? ¿Qué se podría mejorar en la interacción del Programa Nacional y las comunidades? |
| **Superación** | ¿Qué hacen las personas para sentirse mejor? | ¿Qué redes de apoyo hay dentro de la comunidad?  ¿Cómo funciona la asociación de pacientes? |
| **Muestreo de bola de nieve** | ¿A quién más piensa que debería entrevistar? | ¿Qué información me dará esta persona?  ¿Cuál es su área de experiencia? |

**GUÍA INICIAL PARA GRUPOS FOCALES**

| **Temas** | **Preguntas principales** | **Preguntas consecutivas** |
| --- | --- | --- |
| **Prioridades en salud** | ¿Cuáles son los problemas de salud primordiales en su comunidad? |  |
| **Sistema de Salud**  (5 dimensiones de acceso: adaptación, accesibilidad, disponibilidad, asequibilidad, aceptabilidad) | ¿Cómo funciona el sistema de salud en su comunidad? | ¿El actual sistema cubre las necesidades y expectativas de las diferentes poblaciones?  ¿Puede haber problemas de distancias para ciertas poblaciones?  ¿Cómo es la disponibilidad de test diagnósticos y medicamentos?  ¿Los costos son accesibles para todos?  ¿Qué opina sobre los conocimientos y capacidades en el Sistema de Salud para diagnosticar y tratar enfermedades de manera oportuna? |
|  | ¿Qué papel juega la medicina tradicional/ natural en la comunidad? | ¿Cómo se deciden las personas entre tomar medicamentos biomédicos o naturales?  ¿Para qué se toma qué? |
| **Importancia de Chagas** | ¿Qué importancia tiene el Chagas en la comunidad? | ¿Es un tema que se habla frecuentemente?  ¿Se enseña en la escuela?  ¿Cuál es el porcentaje que estima que está infectado? |
|  | ¿En qué situación las personas de la comunidad se deberían realizar el test de Chagas? | ¿Qué partes de la población son vulnerables/afectadas?  ¿Cómo se ve el tema de transmisión durante el embarazo en la comunidad?  ¿Cuántos se estiman que se habrán hecho un test, cuántos han tomado remedios?  ¿Puede haber razones por las cuales alguien de la comunidad prefiera no realizarse el test? |
|  | ¿Qué servicios locales hay para Chagas? | ¿Dónde tiene que acudir una persona si no hay servicios locales? |
|  | ¿Cuáles son los principales problemas causados por el Chagas en su comunidad? | ¿Qué síntomas tienen las personas?  ¿El Chagas produce consecuencias sociales en su comunidad? (por favor, ¿puede describirla, si sabe de alguien que sufrió consecuencias?)  ¿Las personas infectadas tienen las mismas oportunidades de desarrollarse en el trabajo? |
| **Campañas y proyectos anteriores** | ¿Qué experiencia tiene la comunidad con campañas de Chagas? | ¿Hay campañas del Sistema de Salud?  ¿Hubo campañas de ONG internacionales?  En proyectos anteriores, ¿qué se ha hecho bien? ¿Qué se podría mejorar?  ¿Qué ha cambiado después de proyectos anteriores?  ¿Los cambios fueron sostenibles? |
| **Medidas de prevención** | ¿Qué medidas toma la comunidad para protegerse del Chagas? | ¿Cuántas casas estima que tienen vinchucas?  ¿Cómo se cuidan las casas contra la vinchuca?  ¿Hay algún tipo de coordinación a nivel de comunidad para detectar e informar sobre la presencia de vinchucas? |
|  | ¿Qué se debería mejorar para que la comunidad pueda protegerse contra el Chagas? | ¿Qué se debería hacer para que las personas de la comunidad se involucren en actividades preventivas?  ¿Cómo definiría usted un buen proyecto de salud para su comunidad? |
