# Supplementary material for: “We have already heard that the treatment doesn't do anything, so why should we take it?”: A mixed method perspective on Chagas disease knowledge, attitudes, prevention, and treatment behaviour in the Bolivian Chaco
Source: PLoS Negl Trop Dis. 2020 Oct 29;14(10):e0008752. doi: 10.1371/journal.pntd.0008752 (PMC7595318; doi:10.1371/journal.pntd.0008752)
Supplement: S1 Table — (DOCX) [file pntd.0008752.s003.docx]

**S1 Table. Description of included communities.**

| **Community** | **N total** | **Vector infestation 2017** | **Distance to health center** | **Electricity** | **Poverty**  **index** | **MSF** | **CD test** |
| --- | --- | --- | --- | --- | --- | --- | --- |
| 16 de Febrero | 30 | 0 | 1 | Yes | 19.1 | 6.7 | 76.7 |
| Alto Cazadero | 5 | 0 | 25 | No | 100.0 | 20.0 | 40.0 |
| Alto Valle Nuevo | 25 | 0 | 8 | Yes | 73.9 | 40.0 | 84.0 |
| Barrio 21 de Septiembre | 61 | NaN | 1 | Yes | 42.4 | 11.5 | 83.6 |
| Barrio Los Pinos | 103 | NaN | 1 | Yes | 19.1 | 3.9 | 78.6 |
| Barrio Paraíso | 142 | NaN | 1 | Yes | 19.1 | 7.0 | 81.0 |
| Bartolo | 27 | 17.2 | 28 | No | 92.5 | 14.8 | 77.8 |
| Bohorquez | 11 | 31.8 | 18 | No | 98.0 | 18.2 | 72.7 |
| Canizal | 1 | 0 | 20 | Yes | 95.9 | 0.0 | 0.0 |
| Capilla Vieja | 13 | 6.6 | 12 | Yes | 97.6 | 7.7 | 69.2 |
| Cruce Piraymiri | 34 | 0.7 | 0 | Yes | 63.2 | 26.5 | 79.4 |
| Despensas | 17 | 0 | 0 | Yes | 98.2 | 17.7 | 64.7 |
| Hierba Pampa | 13 | 0 | 5 | No | 98.9 | 30.8 | 84.6 |
| Itapenty | 18 | 11.0 | 0 | Yes | 87.6 | 38.9 | 72.2 |
| La Capilla | 12 | 0 | 5 | Yes | 96.0 | 16.7 | 91.7 |
| Los Pinos | 8 | 0 | 15 | No | 100.0 | 12.5 | 62.5 |
| Nogalito | 13 | 0 | 12 | Yes | 95.9 | 15.4 | 61.5 |
| Pedernal | 31 | 0 | 10 | Yes | 86.3 | 19.4 | 45.2 |
| Potreros | 15 | 0 | 25 | No | 100.0 | 6.7 | 73.3 |
| Pucara Alto | 4 | 0 | 3 | Yes | 96.8 | 25.0 | 50.0 |
| San Juan del Piray | 34 | 1.8 | 0 | Yes | 40.1 | 32.4 | 88.2 |
| San Miguel del Bañado | 19 | 5.2 | 0 | Yes | 66.3 | 5.3 | 57.9 |
| Saucecitos | 8 | 0 | 5 | No | 100.0 | 0.0 | 75.0 |
| Sombrerillos | 5 | 1.0 | 6 | Yes | 91.5 | 60.0 | 100.0 |
| Tacuara | 14 | 15.0 | 5 | Yes | 97.5 | 21.4 | 78.6 |
| Uli Uli | 6 | 4.3 | 8 | No | 88.7 | 16.7 | 66.7 |

**N total:** Number of households included from community; **Vector infestation 2017** [11]**:** Official % of community houses found with vector presence within or around the house by National Chagas Program (NaN=Not a Number); **Distance to health center:** km to nearest health center; **Electricity:** availability of electricity within community [25]; **Poverty index:** % of households with unsatisfied basic needs (*NBI=necesidades básicas insatisfechas*) [25]; **MSF:** % of surveyed community members indicating participation in MSF activities; **CD Test:** % of surveyed community members that report testing for CD.
