# Supplementary material for: “We have already heard that the treatment doesn't do anything, so why should we take it?”: A mixed method perspective on Chagas disease knowledge, attitudes, prevention, and treatment behaviour in the Bolivian Chaco
Source: PLoS Negl Trop Dis. 2020 Oct 29;14(10):e0008752. doi: 10.1371/journal.pntd.0008752 (PMC7595318; doi:10.1371/journal.pntd.0008752)
Supplement: S2 Table — (DOCX) [file pntd.0008752.s004.docx]

**S2 Table. Verbatims of study participants.** Original transcribed verbatims of study participants translated into English as well as the original Spanish language versions.

| **Verbatims on Sources of Information** |
| --- |
| V1: [They talk a lot about CD in the community] because, apparently, most of the elderly suffer from it. [...] I have never heard people of my age talking about it. [...] A long time ago, when I was at school, they came and explained a few things [to us]. After this I never heard [about CD] again. (Man, P, K-Y) |
| V1_S: *[En la comunidad se habla mucho de chagas] porque la gente de antes parece que la mayoría sufre de chagas […] entre los jóvenes casi no se escucha así, de mi edad, hablar de chagas. […] Hace mucho, cuando estaba en la escuela, aquí vinieron a explicarnos unas cuantas cosas y ya después de eso no escuche más [sobre chagas]. (Hombre, P, Cl-J)* |
| V2: During the 3 years that I have been at the university, I was never told about this. (Man, R, K-Y) |
| *V2_S: En los tres años que llevo en la universidad nunca se ha hablado de eso, nunca. (Hombre, F, Cl-J)* |
| V3: They always talk about it at school, during campaigns, they used to come to talk about this disease […] here, in the community. (Woman, K-Y, FGD)  The fairs they organize, the talks they give here in the hospital are free for everyone, you can come and listen to learn many things. (Woman, K-Y, FGD) |
| *V3_S: Siempre se habla de esto en la escuela, alguna feria aquí, en la comunidad. Siempre vienen a hablar de esta enfermedad […] aquí, en esta comunidad. (Mujer, Cl-J, GF)*  *Porque las ferias que hacen, las charlas que dan aquí en el hospital, es libre para todos, pueden venir a escuchar y aprender muchas cosas. (Mujer, Cl-J, GF)* |
| **Verbatims on Treatment Decision Process** |
| V4: If you say that you have this disease, there is always someone giving you suggestions [...] We hear so many things. They say “you have to take this, take that, these herbs, these other herbs”. Because of this I haven’t taken anything yet [...]. I live wondering about whether I should take this or that treatment. (Woman, P, K-T) |
| *V4_S: Uno cuando comenta que tiene esa enfermedad siempre hay alguien que te dice esto es bueno […] Ahora lo estoy escuchando: “si tienes chagas toma ivomec […] eso y otro […] el agua de cumanda” […] Pero ninguna de las cosas lo hice […]. Así vivo, pensando si lo hago el tratamiento de esto o no lo hago. (Mujer, P, Cl-M)* |
| V5: I had gone [to the health centre] and joined the line but my neighbours arrived and told me that the tablets had been bad for them. (Woman, P) |
| *V5_S: Yo también fui [al centro de salud] a hacer fila para sacar ficha para hacerme ver y llegan ahí las señoras, mis vecinas igual, otro, otro, me ha hecho mal la tableta, me ha hecho mal la tableta. (Mujer, P)* |
| V6: People only take [treatment] if they see that someone has improved when taking it. Otherwise they don’t want to take the risk. (Man, K-V) |
| *V6_S: Si ven que uno ha mejorado, recién inicia el otro [el tratamiento]. No se arriesgan. (Hombre, Cl-V)* |
| **Verbatims on Knowledge and Misconceptions about Clinical Manifestations of CD** |
| V7: What symptoms does Chagas cause in the beginning? [...] does it have a cure? (Man, R, K-Y) |
| *V7_S: ¿Qué síntomas tiene el chagas, por ejemplo, al iniciar? […] Pero, ¿eso tiene cura? (Hombre, F, Cl-J)* |
| V8: Chagas does not hurt; it is said that it keeps advancing through the veins until finding the heart. Your [CD] can be in your blood for up to 20 years without doing anything. You don't feel it […] it's like a stomach ache, it dries up the gut. (Man, P, K-C) |
| *V8_S: Chagas no duele pues, solamente dice que va avanzando por las venas hasta pillarlo al corazón. Veinte años puede estar tu chagas en tu sangre, no te hace nada, no te sientes […] es como un dolor de estómago, así que es, y a algunos se ha secado la tripa. (Hombre, P, Cl-L)* |
| V9: Chagas causes epilepsy and people don't want to be near someone [with it], they escape and are afraid. This epilepsy causes cerebral trauma and makes [people with it] attack [those around them]. (Man, K-TH) |
| *V9_S: El chagas provoca epilepsia y ya a esa persona poco se le apegan, tratan de escapar no más, la gente ya le tiene miedo. Con esa epilepsia hay trauma cerebral y estas personas tratan de golpear a las personas que estén a su alrededor. (Hombre, Cl-TH)* |
| **Verbatims on Knowledge and Misconceptions about Transmission and Prevention of CD** |
| V10: They should use insecticides, keep their houses clean and keep their animals away from their homes. (Man, K-Y) |
| *V10_S: Deberían fumigarlo, tener limpias sus casas, tener sus animales lejos de su casa. (Hombre, Cl-J)* |
| V11: If you find one [*vinchuca*] out there, one has to hunt it and bring it immediately [to the HC]. (Man, K-Y) |
| *V11_S: Si encuentran digamos uno [insecto vector] por ahí, uno hay que cazar y llevar inmediatamente a entregar acá. (Hombre, Cl-J)* |
| V12: [...] [ivermectin] must have some effect [against CD] because it is a broad-spectrum antiparasitic. (Woman, P, K-HC) |
| *V12_S: [...] [ivermectina] como es antiparasitario de amplio aspecto, debe ser ¿no? que deja algún efecto sobre eso [enfermedad de Chagas]. (Mujer, P, Cl-PS)* |
| **Verbatims on Thread Appraisal** |
| V13: I didn´t believe that it was severe, now I think it is [...] they say that you can die at any time: when you’re going on your way, in your car, or wherever. Chagas is not a joke […] (Woman, P) |
| *V13_S: Yo no pensaba que era grave, ahora yo pienso que es grave […] es donde decían unos mueren en el camino, el auto, en donde sea […] no es chiste ahora. (Mujer, P)* |
| V14: You think that you only have a limited time left. (Woman, P, K-HC) |
| *V14_S: Uno cree que […] tiene una cierta etapa de vida ya marcada y cree que de un tiempo a cierto tiempo ya no hay cura para esto y se va morir. (Mujer, P, Cl-PS)* |
| V15: [The test] came out positive, but I was not very alarmed. I mean, I already knew that all my other siblings had it too [...] We are nonchalant, you see? Only when you are in pain do you go to the doctor. (Woman, P, K-T) |
| *V15_S: [El test diagnóstico] salió positivo, pero no me alarmé tanto, o sea, como ya sabía que mis otros hermanos también tienen [...] ¿No ve? Es uno dejado, así, siempre que te viene un dolor de algo así feo recién vas al médico. (Mujer, P, Cl-M)* |
| V16: [Patients] live from day to day [with it] and don´t see all that the disease implies, because it evidently doesn´t progress from one day to the next. It´s a chronic disease so other things are prioritized. (Woman, K-HC) |
| *V16_S: [Los pacientes] tal vez se concentran en el día a día y no ven todo lo que implica la enfermedad, porque la enfermedad evidentemente no progresa de un día a otro, es una enfermedad crónica, entonces se priorizan otras cosas siempre. (Mujer, Cl-PS)* |
| V17: I think that it´s already been eliminated. In the countryside most of the houses have been improved. [...] I didn´t get my children tested. Before, there were a lot of these bugs, it wasn´t one or two [...] they were flying everywhere, walking all over my sleeping brother [...] you had to make a fire, so that the smoke could disperse them [...] until once we set fire to the roof. (Woman, P, K-T) |
| *V17_S: Creo que se eliminó ya, en el campo ahoritita mayormente ya hay esas viviendas, mejoramiento de vivienda que decimos. [...] Mis hijos, no les he hecho ningún análisis, pero antes había harto ese bicho, no era pues uno o dos que vuelen [...] uuuh volaban por todo, ellos, y mire, mi hermano que estaba por allá durmiendo, por todo su cuerpo caminaba [...] humo hacían dentro de la casa; era de hacer una fogata que el humo lo disperse un poco [...] hasta que [una vez] lo quemamos el techo de la casa porque era de paja. (Mujer, P, Cl-M)* |
| V18: People don´t understand [...] once you eliminate *Triatoma infestans*, triatomae from the jungle will migrate to populated areas, because they won´t have competition [...] Eliminating the *vinchuca* is just a first step in dealing with the problem of Chagas disease. (Woman, K-HC) |
| *V18_S: La gente no entiende realmente [...] eliminando el Triatoma infestans el triatoma que viene de la selva va migrar al centro poblado, porque ya no tiene competencia [...] eliminar la vinchuca es un pasito para mejorar la atención al paciente que tiene chagas. (Mujer, Cl-PS)* |
| **Verbatims on Coping Appraisal** |
| V19: People are tired of finding *vinchucas* in their houses. We don´t want them anymore. We have learned that it is a deadly disease. So of course, [home owners] will fumigate, and buy [their] own insecticides [...] just to prevent it. (Man, R, K-C) |
| *V19_S: Aparece una vinchuca en nuestra casa, el dueño se fatiga. No queremos, ya sabemos cómo es con esas capacitaciones que es una enfermedad mortal. Entonces, bueno, lógicamente el dueño trata de fumigar, el dueño compra su insecticida [...] prevenir, digamos. (Hombre, F, Cl-L)* |
| V20: If [the test] is positive at the beginning and stays positive at the end, you say: I am still sick, I am going to die [...] all this time I took [the medical treatment] for nothing [...] With serologic tests we can see [the disease activity goes] up or down, but the antibodies continue. We can see [from the serologic tests] if the disease has paused, [in other words] if it is not progressing. (Man, K-V) |
| *V20_S: Si [el test] te sale positivo al inicio y te sale positivo al final él va a decir: yo sigo enfermo, me voy a morir [...] tanto tiempo he perdido, mi tiempo tomando ese medicamento y no me hace nada [...] En el tema del estudio serológico vemos si ha bajado o subido, pero los anticuerpos siguen [...] pero si la enfermedad se ha pausado, ya no está siguiendo. (Hombre, Cl-V)* |
| V21: [The treatment] didn´t show any results. According to those who [took the treatment] and repeated the tests it came out positive again [...] it only caused more problems. Before the treatment they didn´t feel anything and now they feel more fatigue [...] apparently the disease progressed with the treatment. That´s why nobody wants to be treated. (Woman, K-Y, FGD)  We have already heard that the treatment doesn´t do anything, so why should we take it? (Man, K-Y, FGD) |
| *V21_S: No han visto los resultados, según nos han contado los que se han hecho [tratamiento] que se han vuelto a hacer la prueba dicen que les ha salido positivo otra vez [...] tal vez les ha causado más enfermedad al ver que antes no sentían nada y dicen que ahora con el tratamiento sienten más síntomas, más cansancio [...] dicen todavía que la enfermedad está más avanzada. Por eso nadie quiere hacer tratamiento. (Mujer, Cl-J, GF)*  *Ya hemos escuchado que ya no hace nada el tratamiento, ¿para qué vamos a hacer? (Hombre, Cl-J, GF)* |
| V22: I see other people who are treated with these tablets and it gave them different reactions [...] some faint, others have swelling, and I'm scared (Woman, P, K-T) |
| *V22_S: Veo en otras personas que también hacen tratamiento con las tabletas que les viene, o sea, reacciones de diferentes maneras [...] algunos se desmayan, otros se hinchan y me da miedo. (Mujer, P, Cl-M)* |
| V23: We were educated that this is a chronic disease and that the treatment has more negative effects than benefits. I am talking about 7-8 years ago [...] This is how we treated patients. (Woman, K-HC, FGD) |
| *V23_S: Hemos tenido una educación en que esta enfermedad es una enfermedad crónica y que el tratamiento ha tenido más efectos adversos que beneficios. Hablo como de unos 7-8 años [...] a los pacientes con ese concepto se les ha tratado. (Mujer, Cl-PS, GF)* |
| V24: We generally forbid [patients] to eat pork, fat, soda and alcoholic beverages, spicy food in general [...] a lot of people say: “I won´t work, what will I do during those days”. Because here the sun shines strongly, those that work in agriculture feel conflicted [...] They say “If you forbid me to eat so many things, then what will I eat […]?” (Woman, K-HC) |
| *V24_S: Generalmente se prohíbe el chancho, las grasas, está la soda, las bebidas alcohólicas, los picantes generalmente [...] hay muchos que dicen “no voy a trabajar, qué voy a hacer esos días” porque acá el sol es fuerte, verdad, entonces eso sí, los que son de ocupación agricultor, sí se dificultan [...] Dicen “yo no tengo esto, entonces ¿qué voy a comer si me prohíben comer esto y aquello? (Mujer, Cl-PS)* |
| V25: They approach you at the vets and tell you: “Doctor, I want you to sell me Ivomec, I have CD and I want to be cured” [...] People believe in it more because they say that they feel better, that the relief is fast. (Man, K-V) |
| *V25_S: Se acerca ahí, en la veterinaria, y les dice prácticamente: “Doctor, quiero que me venda Ivomec, tengo chagas, quiero curarme” [...] La gente le tiene más fe al tema de uso veterinario porque ellos mismos dicen que se sienten mejor, el alivio es rápido. (Hombre, Cl-V)* |
| **Verbatims on Primary Preventive Practices** |
| V26: We sprayed the insecticide that we bought with our own money. You don´t see the [vectors] anymore. (Woman, P) |
| *V26_S: Se ha fumigado, nosotros con nuestra propia plata hemos comprado y hemos empezado a fumigar, no hay más [vectores]. (Mujer, P)* |
| V27: Sometimes [the people] continue living in their old homes [...] if we have housing improvement projects in a community, we should teach the community to destroy the old ones. (Man, K-AU) |
| *V27_S: Algunas veces siguen viviendo en su casa antigua [...] si bien entramos con mejoramiento a una comunidad, lo bueno sería instruir a la comunidad que esa casa antigua lo deshaga. (Hombre, Cl-Au)* |
| **Verbatims on Early Diagnosis and Treatment** |
| V28: I came for something else and there was sign that said “Chagas” and I said, “why don't I use my waiting time to do the CD test, to remove my doubts”. I got in and they said: “it's for free” and that was all. (Woman, P, K-T) |
| *V28_S: A otra cosa vine aquí al médico y entonces pensé, sentada ahí estaba y miré. “El Chagas”, decía. “Ay, por qué no aprovecho mientras espero de hacerme el análisis del chagas y de una vez voy a salir de dudas” dije, y entonces entré. “Es gratuito”, me dijeron, “vaya no más y listo”. (Mujer, P, Cl-M)* |
| V29: They offered voluntary testing [at the university]. I saw the announcement with a group of female friends. (Woman, P, K-HC) |
| *V29_S: Un día vimos el aviso ahí [en la universidad] donde decía que quiénes querían hacerse de forma voluntaria en el laboratorio y bueno, en un grupo de amigas tomamos la opción y nos hacemos. (Mujer, P, Cl-PS)* |
| V30: During the campaign most of the youth came there saying “I want to know; I want to know if I have CD”. (Woman, K-HC, FGD) |
| *V30_S: Cuando estuvimos en campaña han venido la mayoría de los jóvenes “yo quiero saber si tengo chagas, yo quiero saber si tengo chagas”. (Mujer, Cl-PS, GF)* |
| V31: Not even 50% come back for their planned treatment. Sometimes they prioritize social festivities, their work, because they have to comply with their obligations or other pathologies that can be treated more easily. (Man, K-HC) |
| *V31_S: Ni 50% termina volviendo a sus consultas que ya ha programado para el inicio de tratamiento. Una persona a veces prioriza fiestas, prioriza algunas veces el trabajo porque tiene que cumplir sus obligaciones, incluso otras patologías que pueden ser todavía tratadas o procedimientos más sencillos, pero los prioriza. (Hombre, Cl-PS)* |
| V32: In order to treat heart conditions, we use other things. (Man, K-TH) |
| *V32_S: Nosotros para hacer tratamiento del corazón, aplicamos otras cosas. (Hombre, Cl-CT)* |
| V33: Yes, there is a doctor […] he has prescribed [veterinary ivermectin] to some of the patients. (Woman, P, K-HC) |
| *V33_S: Sí, sí, hay un médico [...] algunos que tienen se les ha recetado [ivermectina veterinaria]. (Mujer, P, Cl-PS)* |
| V34: I went to all the doctors, then to a company who make medicines, they sell herbs, powders, solutions [...] I brought it back from far away, it costed me 900 (bolivianos). This [alternative treatment] has helped me a little.  [...] After that I took Ivomec; people made me take it for CD [...] it helped me, I took it for a long time, I felt as if it cured me. I wasn't feeling tired [...] I could wash, cook, walk. (Woman, P) |
| *V34_S: A toditos los médicos, después a una empresa de los que hacen medicamentos, que hacen, ya, venden hierbas, ya, venden polvos para tomar aguas [...] también me he ido a traer de lejos ese, con novecientos [bolivianos] compré una caja, ese he estado tomando, pero ese un poco me ha hecho [tratamiento alternativo]. [...] Después tomé Ivomec, la gente me ha hecho tomar para el chagas [...] me ha ayudado, he estado tiempo con eso, como si estuviera sano andaba, no me cansaba, ya podía hacer, ya podía lavar, ya podía cocinar, ya podía andar. (Mujer, P)* |
| V35: If in a community there is a person who has done the treatment, that has shown results and has controlled the disease [...] we should use that person -with their consent- to be an example within the community. (Man, K-V) |
| *V35_S: Si en alguna comunidad hay una persona que ha hecho el tratamiento, que se ha hecho los resultados y se ha controlado la enfermedad [...] utilizarla a esa persona, con el mismo consentimiento de esa persona, ahí está el ejemplo en la misma comunidad. (Hombre, Cl-V)* |
| **Verbatims on Management of Chronic Complications of CD** |
| V36: We need to increase our services. Because, even if we give antiparasitic treatment, we are not taking care of the population that already has an advanced pathology, a visceral complication. And this population does not have access to any kind of help [...] the insurance doesn't cover it. (Woman, K-HC) |
| *V36_S: Nos falta también ampliar la gama de atención, porque si bien se da tratamiento etiológico a la enfermedad, estamos desatendiendo a la población que ya tiene una patología avanzada, una complicación visceral, y esta población no tiene acceso a ningún tipo de servicio [...] el seguro no cubre. (Mujer, Cl-PS)* |
| V37: To have a specific place for patients with chronic cardiac and gastric diseases in order to avoid more complications. (Woman, K-HC) |
| *V37_S: Tener un lugar específico para pacientes que tengan ya patologías cardiacas y digestivas, entonces para evitar las complicaciones más severas. (Mujer, Cl-PS)* |
| V38: My brother became ill with CD [...] it cost around 5000 dollars. He was sick for a long time and then died young. (Man, P, K-HC, FGD)  A patient that can't go to a basic reference hospital will not benefit [not even from a free pacemaker campaign] and will just wait for death. (Woman, K-HC, FGD) |
| *V38_S: Mi hermano ha enfermado de chagas [...] costaba algo de cinco mil dólares. Así mucho tiempo ha estado enfermo hasta que falleció joven. (Hombre, P, Cl-PS, GF)*  *Una paciente que no va a poder acudir hasta un hospital de referencia básica no se va beneficiar [de una campaña de marcapasos gratuitos] y solo va esperar la muerte. (Mujer, Cl-PS, GF)* |
| V39: [...] sometimes we have to get them out of hospital [...] let's say tomorrow the pacemaker surgery is planned, so tonight we do everything we can to get him out of there [...] pacemakers are prone to miscalibrations over time. Someone with a pacemaker can be walking through the street, if there is a lightning bolt then it can discharge and it’s all over. (Man, K-TH) |
| *V39_S: [...] a veces tenemos que sacarle del hospital [...] digamos mañana se declara la operación de colocarle marcapasos, esta noche hace lo posible el paciente de salirse de ahí [...] Los marcapasos están propensos a una descalibración del tiempo. Uno con marcapasos puede estar pasando por esta calle y tira un trajín [rayo] el tiempo, con eso se descarga y listo. (Hombre, Cl-CT)* |
| V40: There are superstitions. People believe that someone with a pacemaker can get struck by lightning or be affected by rain or thunder or by using domestic electronics. This fear makes them take a step back when we have the possibility to help them. (Man, K-HC) |
| *V40_S: Hay supersticiones donde la gente cree que un portador con marcapaso la gente tiene temor a que le caiga un rayo, o que le afecte la lluvia o los truenos, o a usar aparatos electrodomésticos. Y ese temor les hace dar un paso atrás cuando tenemos la posibilidad de ayudarles. (Hombre, Cl-PS)* |
| **Verbatims on Healthcare System Barriers and Social Determinants of CD** |
| V41: When the rivers rise [from January to April] the road breaks down, we are cut off. (Woman, K-HC, FGD)  Last time we organized a transfer, the patient was stable [...] The other ambulance came from Monteagudo and he had to walk [through the river] with his i.v. infusions. (Man, P, K-HC, FGD) |
| *V41_S: Se padece mucho de la crecida de ríos [de enero a abril], se ‘frega’ el camino, a veces nos quedamos encerrados aquí. (Mujer, Cl-PS, GF)*  *La anterior vez hemos hecho un traspaso, hemos llegado hasta donde es el camino y ese paciente era un poco estable [...] Ha venido la otra ambulancia de Monteagudo, ha tenido que ir con el suero así, caminando [cruzando el río]. (Hombre, P, Cl-PS, GF)* |
| V42: As rural people we want to be attended to as we are. We arrive at the hospital and the first thing they say to us is: “take a shower” [...] people prefer to suffer and die and then to go to hospital [...] As traditional healers we have to go hand in hand with the patient [...] you have to go to the patient, not the patient to the doctor. (Man, K-TH) |
| *V42_S: La gente de campo queremos que nos atiendan tal como somos, tal como queremos. Vamos al hospital y lo primero que nos dicen es “báñese” [...] prefiere la gente sufrir morir y no ir al hospital [...] Los médicos tradicionales tienen que andar de la mano [...] esa es la forma de combatir la enfermedad; buscar al paciente, no el paciente al médico. (Hombre, Cl-CT)* |
| V43: They are more preoccupied as to what to eat, about how much money they have for tomorrow, because there is a lot of need in the villages. Even if there is a campaign for their own good, they might say: I have to work. (Man, R, K-Y) |
| *V43_S: Más se preocupan por comer o, digamos, cuánto dinero van a tener mañana. De eso se preocupan, porque hay harta necesidad, no ve, en los pueblos y, digamos, cuando ven una campaña así, aunque sea por su bien, ellos dicen “tengo que trabajar”. Por eso prefieren irse a trabajar. (Hombre, F, Cl-J)* |
| V44: I think it also has to do with a lack of education, which is misused by [other veterinarians] that sell [ivermectin]. (Man, K-V) |
| *V44_S: Yo creo que ese es el criterio más de la gente que no ha tenido un nivel de formación, que de esa parte se aprovecha también aquel colega [profesional] que vende su producto. (Hombre, Cl-V)* |
| V45: [...] to make the people in charge of economic resources understand, that this is a disease that is close to their family, that is close to their neighbors, that is close to everyone. We all have had friends, relatives, that have died of CD [...] [Through pacemaker implantation] we don't just heal a person, we healed a mother [...] that could have left orphans within a short time. There are figures that we didn't yet analyze, in terms of poverty and maternal mortality. (Woman, K-HC) |
| *V45_S: [...] hacerles ver a las personas de quienes depende el recurso económico que es una enfermedad que está cerca de su familia, que está cerca de sus vecinos, que está cerca de todos, porque todos provenimos prácticamente del área rural y todos en nuestra historia familiar hemos tenido conocidos, familiares, que han muerto por chagas. [...] [Al implantar un marcapasos] estamos no solamente curando a una persona, estamos curando a una mamá; quizás evitándole una cardiopatía chagásica que dentro de poco va dejar huérfanos a sus hijitos por la mortalidad. Hay figuras que no están analizando todavía; dentro de la lucha contra la pobreza, la mortalidad materna [...] (Mujer, Cl-PS)* |
| V46: [The authorities] make this housing improvement political. [...] Some people’s houses were quickly improved even though they had nice houses to begin with, and down there there are houses that are falling apart. [...] [housing improvement] should be for everyone or no one. (Man, K-TH) |
| *V46_S: Este mejoramiento de vivienda lo toman políticamente [...] se les ha dado mejoramiento a los que tienen lindas casas y allá en el canto, allá abajo hay casitas que se están cayendo. [...] El mejoramiento debería llegar para todos o para nadie. (Hombre, Cl-CT)* |
| V47: [Housing improvement] comes from God. Whoever gets it gets it, it's not the poorest [...] There are only maybe 10 tests for the canton. [...] [Regarding distribution of treatment during campaigns] It seems that the nurse choses who gets them on her own, for family or friends [...] and others that are in grave need she doesn’t inform, because there wouldn’t be enough. (Man, R, K-C) |
| *V47_S: Bueno, eso [mejoramiento de viviendas] vino de Dios; al que le tocó, le tocó. Al que no, aunque al más humilde que no a veces [...] [Los análisis] vienen contaditos, no sé cuantitos; por aquí al cantón parece que llega unos diez test para hacer análisis. [...] [El tratamiento] viene en campañas. Eligen, parece, a su modo de ver de la enfermera, otros parece que familiarmente amigos de ella, eligen tal, tal y listo. Y otros que están de gravedad, lógico, no los avisa porque va faltar. (Hombre, R, Cl-L)* |
| **Verbatims on Stigma and Discrimination** |
| V48: Sometimes among friends we hear: “Ah, you have Chagas”. It’s already a normal topic between us. (Man, K-V) |
| *V48_S: A veces, entre los mismos amigos, uno se entera. Si “vos tienes chagas” ya es normal el tema entre nosotros. (Hombre, Cl-V)* |
| V49: We have to inform [the society/companies] that with this disease you won ́t work less and won’t have deficiencies [...] you can work and be as efficient as anybody else. (Woman, P, K-HC) |
| *V49_S: Brindando información [a la sociedad, las empresas] tal enfermedad, digamos chagas, no hace que trabajes menos o tengas ciertas deficiencias a nivel laboral [...] puedes trabajar y ser tan eficiente como cualquier otro trabajador siendo chagásico. (Mujer, P, Cl-PS)* |
| V50: We came to this extreme situation that they ask for a CD test as a requisite for a loan, depending on the amount [...] and if it's positive, they don't give it [...] that’s discriminative. Nobody complains, nobody raised their voices [...] they should report it. (Man, K-V) |
| *V50_S: Llegamos a ese extremo de pedir resultados de laboratorio como un requisito, dependiendo del monto de dinero [...] y si salen positivos no les dan [...] eso es discriminatorio. Ahí está que nadie se ha quejado tanto, nadie ha levantado su voz [...] lo que se debería hacer, lo que siempre digo en la oficina, es denunciar. (Hombre, Cl-V)* |
| V51: The majority of companies ask for [the analysis] [...] Chagas affects the heart, I think that's why. (Man, P, K-Y) |
| *V51_S: En la mayoría de empresas donde entra creo que piden eso. Te hacen análisis [...] como dicen, el chagas afecta al corazón, y pienso por eso. (Hombre, P, Cl-J)* |
| **Verbatims on Lessons Learned from Former Projects and Priorities for Future Interventions** |
| V52: Chagas was given a level of importance since MSF came. Before this it was not diagnosed nor treated in health posts [...] only in the hospital, only those under the age of fifteen took the treatment. [...] Now we treat it just like any other disease. (Woman, K-HC) |
| *V52_S: El enfoque hacia chagas acá se ha dado la importancia desde que Médicos Sin Fronteras ha ingresado. Anterior a eso no se diagnosticaba, no se trataba en los puestos de salud [...] solamente en el hospital, solo tomaban la pauta menores de quince años. [...] Ahora sí lo manejamos como cualquier enfermedad. (Mujer, Cl-PS)* |
| V53: I think that there has been a change, with this awareness now and with these doctors for free that see us everywhere, this motivates us to get a cure, to have treatment. Now with that capacity we try even more to take care of ourselves. (Man, R, K-C) |
| *V53_S: Ahora sí ya está cambiando, más que todo con esta alerta que ya, ahora, ya hay esos médicos gratis que nos atienden en cualquier lado, dan ánimos de hacerse curar, de hacerse un tratamiento. Eso es lo bueno que entonces ya ahora, ya con esos cursos, más tratamos de cuidarnos. (Hombre, R, Cl-L)* |
| V54: MSF wasn't here for long. They arrived with the news that they would treat the whole population and there were elderly patients-over 80- that wanted to start treatment [...] they arrived advertising [...] that the whole population would be treated, would be cured. [...] And everybody came: “I want to be treated, I want to be treated”, but we had to explain that no, that you had to see in what stage the disease was. There are patients that are still coming [...] that became aware of it now, but the problem is that there are no medicines now [...] You have to guarantee the treatment of the patients and not to arrive, to treat some and then say to the others that are positive "that’s as far as we get”. (Woman, P, K-HC) |
| *V54_S: En realidad Médicos Sin Fronteras no estuvo mucho tiempo. Llegaron con la noticia de que se iba a hacer tratamiento a toda la población y había pacientes, personas de la tercera edad, que querían; ochenta años a partir esa edad ya querían iniciar tratamiento [...] llegaron con propaganda [...] de que la población que iba a hacer tratamiento iba a ser curada [...] “Quiero iniciar, quiero iniciar tratamiento”, venían las personas, pero se les tenía que explicar que no, que hay que ver primero la etapa donde está su enfermedad para iniciar tratamiento. Hay pacientes que después de eso quieren hacerse todavía [...] Pareciera que toman conciencia y ahora sí quieren hacerse el tratamiento; el problema es que no hay el medicamento [...] Para iniciar tratamientos, garantizar los tratamientos de los pacientes. O sea, no es llegar, iniciar ciertos y después, bueno, los que dan positivos “hasta aquí llegamos, los medicamentos no hay más y aquí nos quedamos”. (Mujer, P, Cl-PS)* |
| V55: They organized workshops, trainings [...] so we inform people from our community. I feel, how should I say this, they didn’t ́t come back again [...] between us we try to keep the [houses vector free], we know how to prevent. (Man, R, K-C) |
| *V55_S: Ellos nos daban taller, cursos de capacitación [...] así informamos a la gente de nuestra comunidad. Yo me siento, no sé cómo decirlo, ellos nos dijeron así pero no vinieron más [...] casi que entre nosotros no más nos tratamos de mantener, ya sabemos cómo es la enfermedad y por eso la prevención. (Hombre, R, K-C)* |
| V56: [...] organizing the patients so that they become a powerful society [...] the organized population can learn a little more about interventions and support themselves, by being step by step integrated into the system. So, it’s participatory in both ways, I think that that is very important. (Man, K-HC) |
| *V56_S: [...] organizándoles a los mismos pacientes, o sea, ellos se convierten en un poder social [...] también es darle la posibilidad de participar en la intervención, no que solamente sea del sistema hacia la asociación, sino que también la misma población organizada pueda aprender un poco más el tema de una intervención y refuerce, ¿no?, y se vaya integrando al sistema, que sea de ida y vuelta, participativo. Creo que eso es muy importante. (Hombre, Cl-PS)* |
| V57: [CD] is not a topic in the veterinary associations [...] it was never taken up, not even at congresses, we never had any meeting about it. (Man, K-V). |
| *V57_S: [En el colegio de veterinarios] Nunca se ha tomado ese tema [enfermedad de Chagas] ni en un congreso, digamos, que hemos tenido, ni en ninguna reunión. (Hombre, Cl-V).* |

Vx: verbatims translated into English language

Vx_S: original verbatims transcribed in Spanish language

FGD: Focus Group Discussion; P: patient; R: patient’s relative; K: key informant (HC: healthcare staff -medical doctor, nurse, etc.-; V: veterinary doctor; TH: traditional healer; T: teacher, AU= Authority, Y= Youth, C=Community Leader/ PIV (Vector information office)).

*GF: Grupo Focal; P: Paciente; F: Familiar de paciente; Cl: Informante Clave (PS: Personal Sanitario -médico/a, enfermero/a, etc.-; V: veterinario/a; CT: Curandero/a Tradicional; M: Maestro/a, J: Joven, AU: Autoridad, L: Líder de la comunidad /PIV (Punto de Información Vectorial)).*
