# Supplementary material for: “We have already heard that the treatment doesn't do anything, so why should we take it?”: A mixed method perspective on Chagas disease knowledge, attitudes, prevention, and treatment behaviour in the Bolivian Chaco
Source: PLoS Negl Trop Dis. 2020 Oct 29;14(10):e0008752. doi: 10.1371/journal.pntd.0008752 (PMC7595318; doi:10.1371/journal.pntd.0008752)
Supplement: S3 Table — (DOCX) [file pntd.0008752.s005.docx]

**S3 Table. Information sources and level of knowledge on CD.**

| **Source of Information** | **Number of participants** | **Percentage of participants (N=669)** |
| --- | --- | --- |
| Healthcare Center | 295 | 44.1 |
| Health campaign | 233 | 34.8 |
| Experience of someone close | 215 | 32.1 |
| Family | 204 | 30.5 |
| Hospital | 182 | 27.2 |
| Radio / TV | 155 | 23.2 |
| School | 87 | 13.0 |
| Internet / Social media | 21 | 3.1 |
| **Knowledge on transmission** | **Number of participants** | **Percentage of participants (N=669)** |
| No correct answer | 61 | 9.1 |
| Vectorial | 605 | 90.4 |
| Vertical | 247 | 36.9 |
| Blood / organ donation | 174 | 26.0 |
| Oral | 41 | 6.1 |
| **Misconceptions on transmission** | **Number of participants** | **Percentage of participants (N=669)** |
| Sexual transmission | 6 | 0.9 |
| Witchcraft | 2 | 0.3 |
| Contact with patient | 20 | 3.0 |
| Other (sharing utensil, cloths) | 10 | 1.5 |
| **Clinical knowledge on acute CD** | **Number of participants** | **Percentage of participants (N=669)** |
| No correct answer | 581 | 86.8 |
| Fever | 47 | 7.0 |
| Romana Sign | 19 | 2.8 |
| Chagoma | 6 | 0.9 |
| Asymptomatic | 22 | 3.3 |
| **Knowledge that latent phase can take up to decades (more than 10 years)** | | |
| Latent phase >10 years | 173 | 25.9 |
| **Clinical knowledge on chronic CD** | **Number of participants** | **Percentage of participants (N=669)** |
| No correct answer | 129 | 19.3 |
| Chest pain | 296 | 44.2 |
| Dyspnea | 249 | 37.2 |
| Irregular heartbeat | 84 | 12.6 |
| Brady / Tachycardia | 115 | 17.2 |
| Syncope | 118 | 17.6 |
| Edema | 129 | 19.3 |
| Dysphagia | 12 | 1.8 |
| Chronic Obstipation | 118 | 17.6 |
| **Knowledge on primary prevention** | **Number of participants** | **Percentage of participants (N=669)** |
| Improvement / Cleanliness housing | 412 | 61.6 |
| Insecticides spraying | 331 | 49.5 |
| Keeping animals at distance | 187 | 28.0 |
| **Knowledge on secondary prevention** | **Number of participants** | **Percentage of participants (N=669)** |
| Test during pregnancy | 55 | 8.2 |
| Early diagnosis | 102 | 15.2 |
| Early treatment | 131 | 19.6 |
| **Prevention without evidence** | **Number of participants** | **Percentage of participants (N=669)** |
| Cumanda | 117 | 17.5 |
| Honey / Propolis | 20 | 3.0 |
| Veterinary ivermectin | 109 | 16.3 |
