# Supplementary material for: “We have already heard that the treatment doesn't do anything, so why should we take it?”: A mixed method perspective on Chagas disease knowledge, attitudes, prevention, and treatment behaviour in the Bolivian Chaco
Source: PLoS Negl Trop Dis. 2020 Oct 29;14(10):e0008752. doi: 10.1371/journal.pntd.0008752 (PMC7595318; doi:10.1371/journal.pntd.0008752)
Supplement: S4 Table — (DOCX) [file pntd.0008752.s006.docx]

**S4 Table. Primary preventive practices of study participants against CD (re-)infection.**

| **Effective primary preventive practices** | **Number of participants** | **Percentage of participants (N=669)** |
| --- | --- | --- |
| No effective preventive practice | 272 | 40.7 |
| Household cleaning | 365 | 54.6 |
| Plastering of walls | 9 | 1.3 |
| Improvement of housing conditions | 34 | 5.1 |
| Keeping animals at distance | 12 | 1.8 |
| Moving of furniture | 10 | 1.5 |
| Physical barriers e.g. window/ bed nets | 6 | 0.9 |
| Routine Search for vectors | 4 | 0.6 |
| **Practice with doubtful effectiveness** | **Number of participants** | **Percentage of participants (N=669)** |
| Use of household insecticides | 174 | 26.0 |
| Traditional herbs | 9 | 1.3 |
| **Animals in/near (<50 m) household** | **Number of participants** | **Percentage of participants (N=669)** |
| No animals | 195 | 29.1 |
| Chickens | 349 | 52.2 |
| Dogs | 314 | 47.0 |
| Cats | 137 | 20.5 |
| Pigs | 74 | 11.1 |
| Ducks | 41 | 6.1 |
| Cows | 36 | 5.4 |
| **Self-reported vector presence** | **Number of participants** | **Percentage of participants (N=669)** |
| Current vector presence at the house | 72 | 10.8 |
| Unsure of current vector presence | 10 | 1.5 |
| Vector presence at the house during last year | 186 | 27.8 |
| Previous report of vector presence to authorities | 173 | 25.9 |
| **Response of NCP** | **Number of participants** | **Percentage of participants (N=173)** |
| NCP came to spray | 107 | 61.8 |
| No response by NCP | 48 | 27.8 |
| Inconclusive response | 18 | 10.4 |
