# Supplementary material for: “We have already heard that the treatment doesn't do anything, so why should we take it?”: A mixed method perspective on Chagas disease knowledge, attitudes, prevention, and treatment behaviour in the Bolivian Chaco
Source: PLoS Negl Trop Dis. 2020 Oct 29;14(10):e0008752. doi: 10.1371/journal.pntd.0008752 (PMC7595318; doi:10.1371/journal.pntd.0008752)

**S1 Fig. Effect of socioeconomic factors on the answers to questions by education level, employment and rural/urban dwelling.**

In examining the impact of socio-economic status (SES) on respondents’ knowledge and attitudes related to CD, three interview questions were selected to use as indicators of SES: rural/urban dwelling (binary); education level (four groups ranging from no education/incomplete primary school to tertiary education and above); and employment (unemployed, student, farming and employed). We used a Chi-squared hypothesis test to test for statistically significant relationships between responses to salient CD-related questions and SES. Response count data were recorded in contingency tables, with each table comparing a CD-related question (e.g. “Can CD be transmitted from mother to child during pregnancy?”) to an SES variable (e.g. education level). A chi-squared test statistic for each table was computed by calculating the sum of the normalized differences between the distribution of the response to the CD-related question between the different socio-economic groups. The CD-related questions were split into three groups: Questions related to access (labelled A questions), questions related to believe (labelled B questions), and questions related to the domestic situation of the respondent (labeled C questions). Histograms for each SES class were created for all questions, and the significance between the distributions was used to annotate each plot. The test statistic was used to compute a p-value at the 95% level. ns is p>0.05, * is 0.01<p<0.05, ** is 0.001<p<0.01, *** is p<0.001 and **** is p<0.0001.

S1A1 Fig. Telephone network coverage.


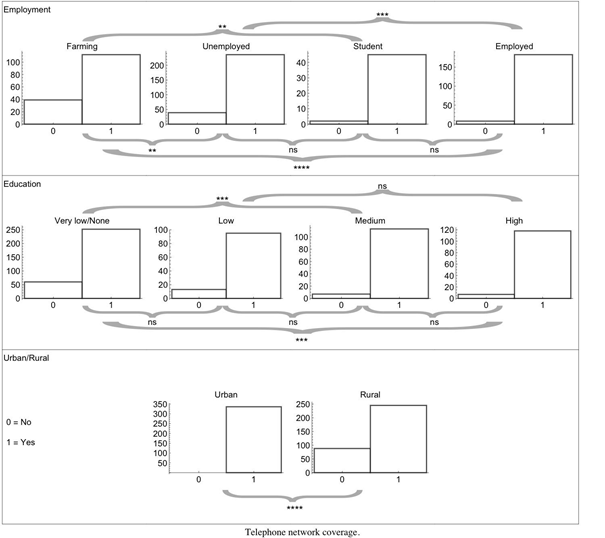


S1A2 Fig. Access to electricity.


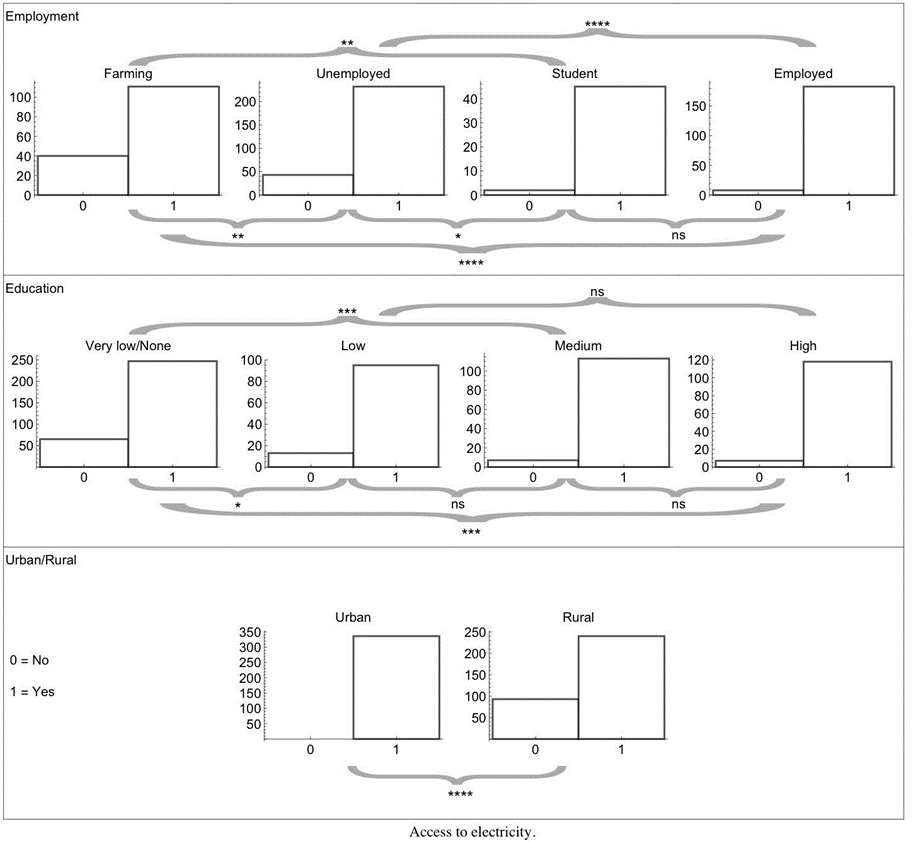


S1A3 Fig. Health insurance.


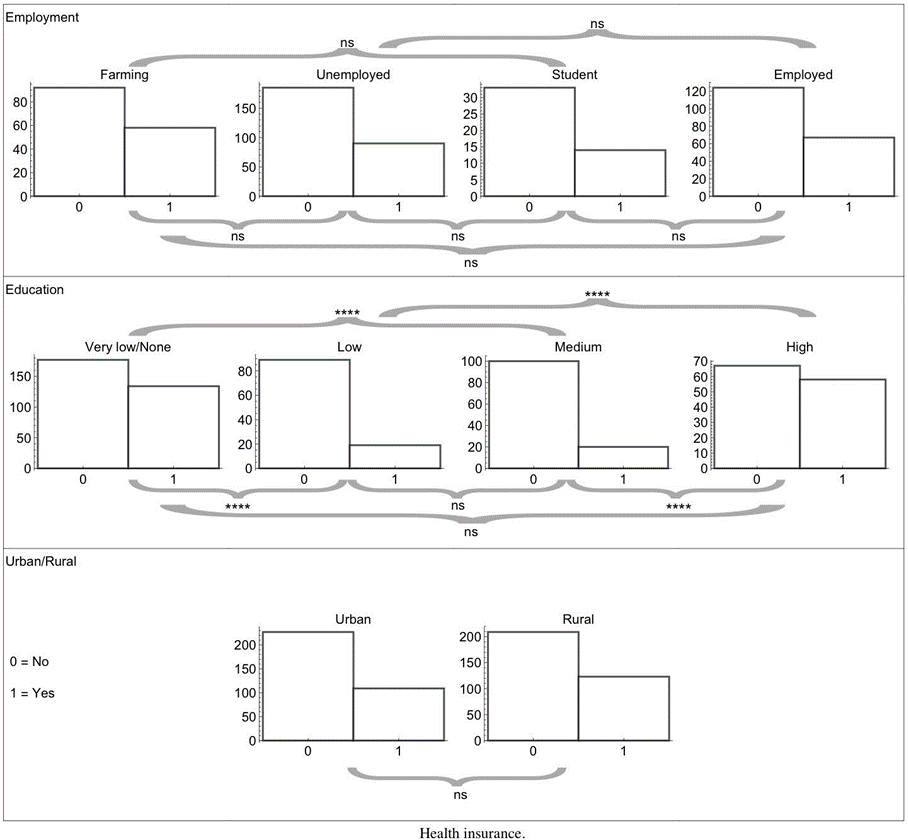


S1A4 Fig. Cost is a reason I could not get the medical help I need.


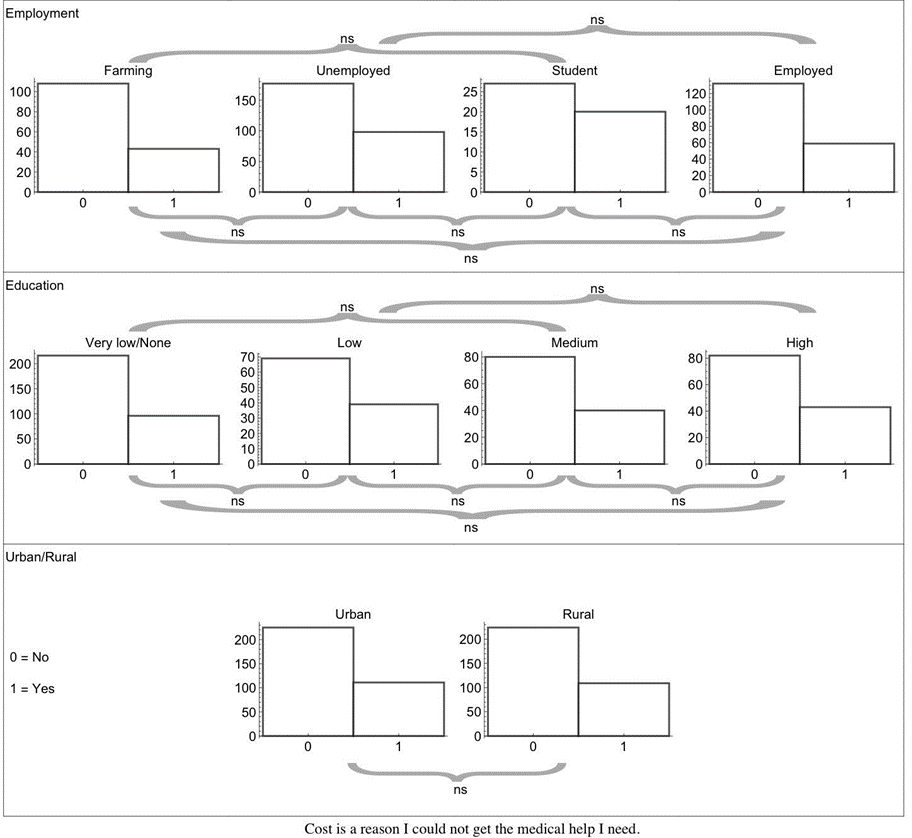


S1A5 Fig. Distance is a reason I could not get the medical help I need.


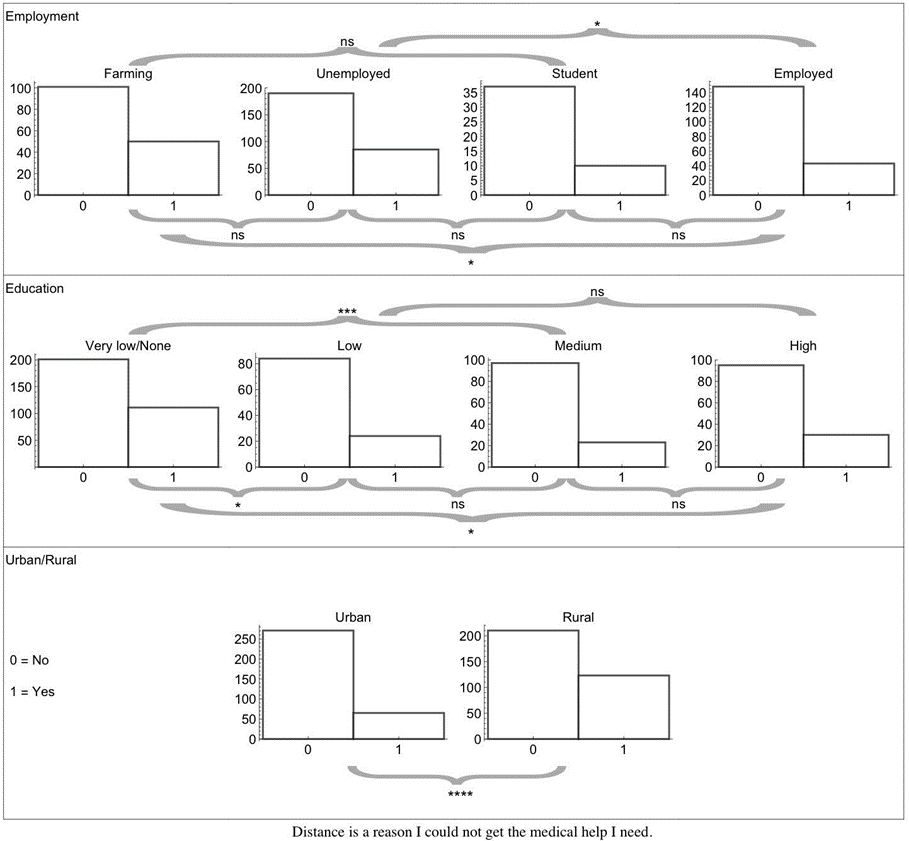


S1A6 Fig: Lack of stock of tests is a reason I could not get the medical help I need.


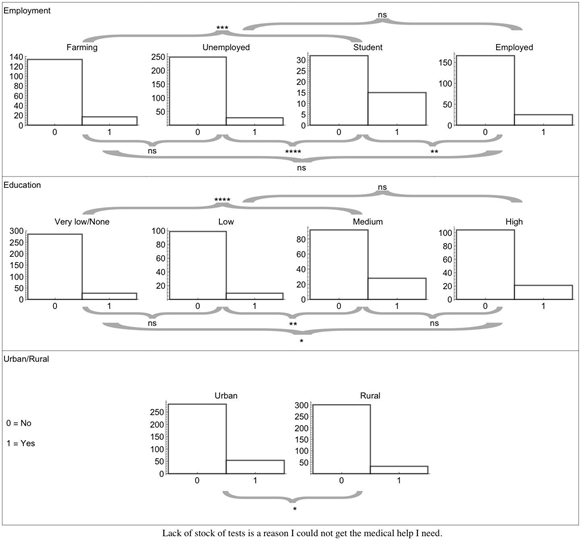


S1A7 Fig: Lack of medical knowledge is a reason I could not get the medical help I need.


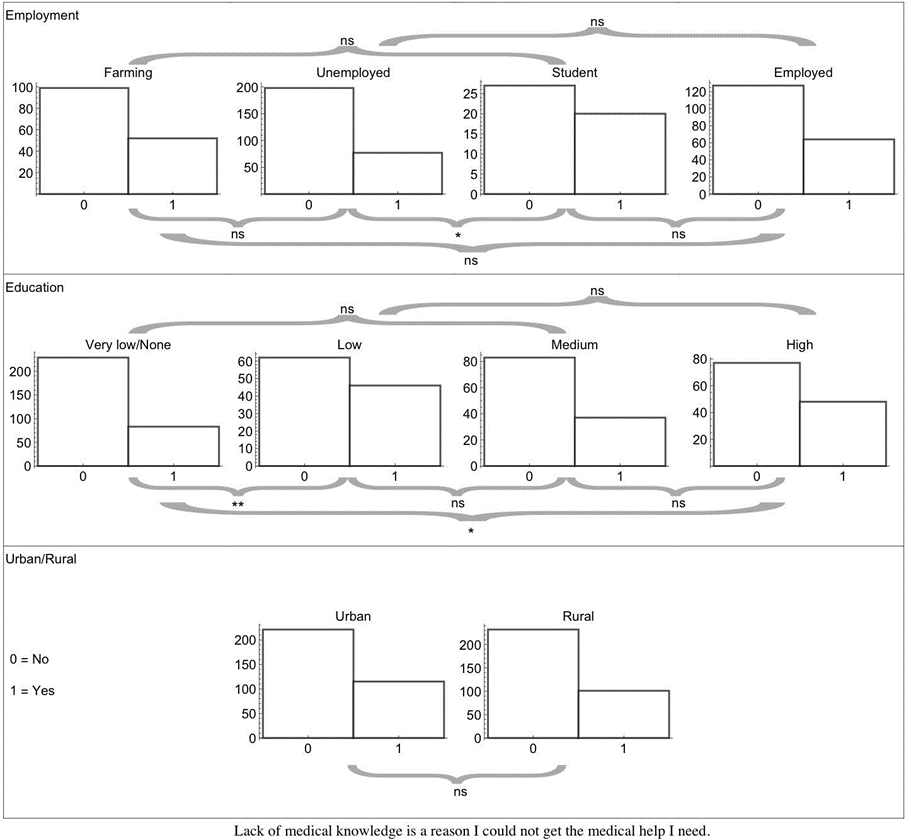


S1A8 Fig: Lack of medicine is a reason I could not get the medical help I need.


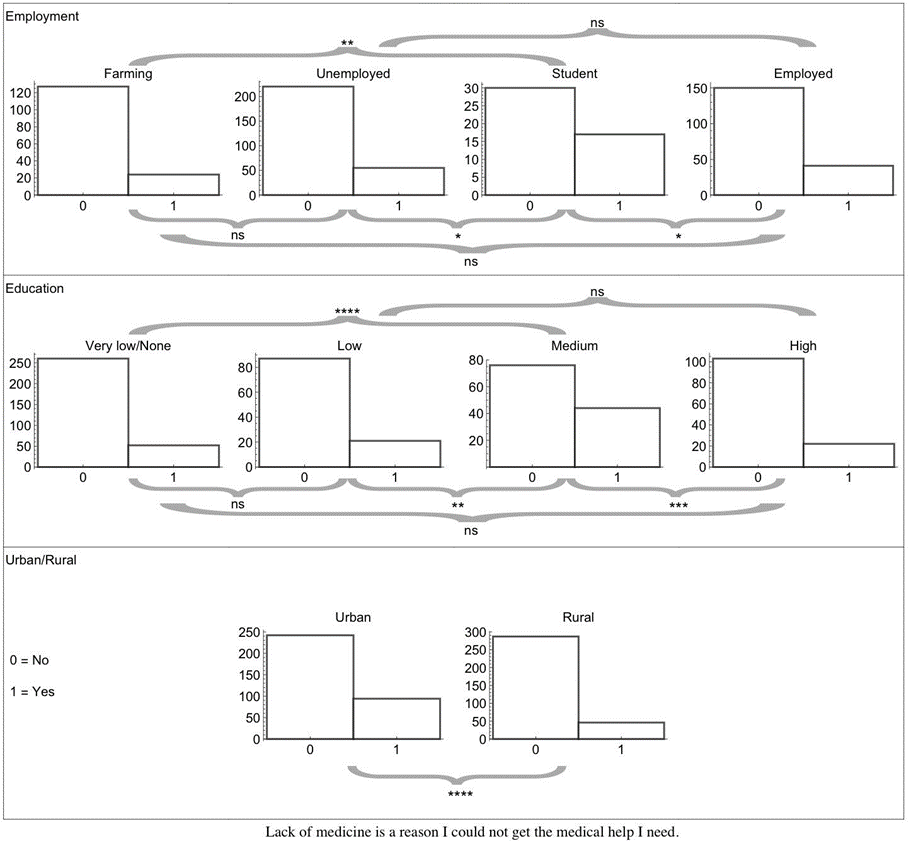


S1A9 Fig: Poor/low quality treatment is a reason I could not get the medical help I need.


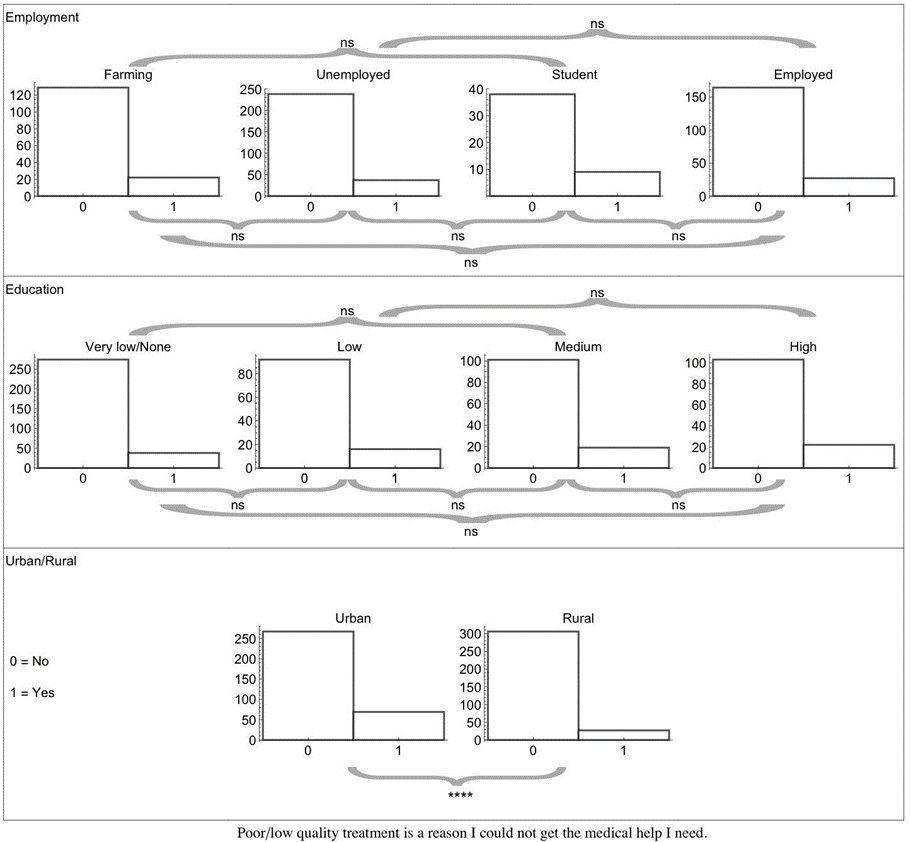


S1A10 Fig: I can't miss days of work to get medical help.


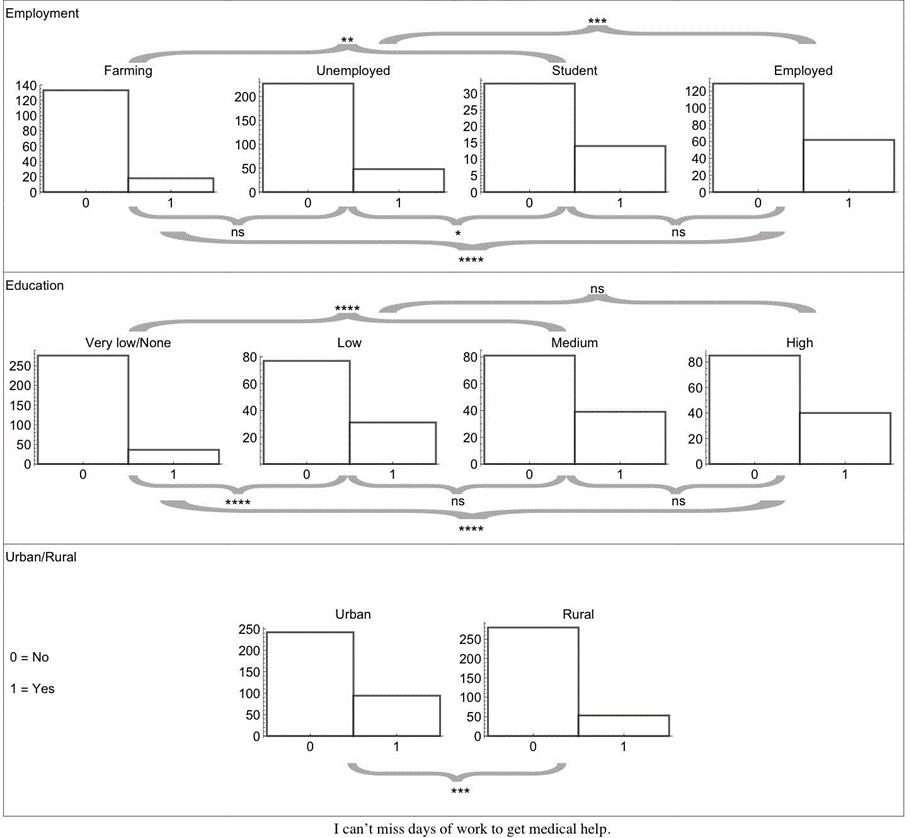


S1A11 Fig: Lack of health insurance is a reason I could not get the medical help I need.


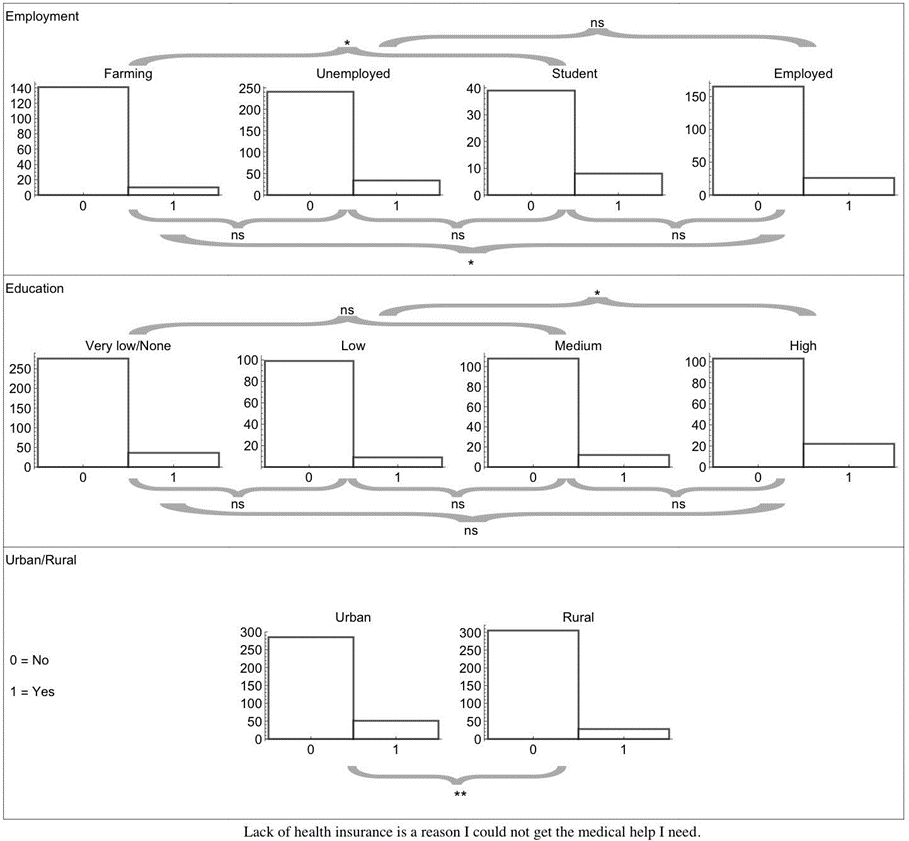


S1A12 Fig: Did you have any problems accessing health care during the last year?


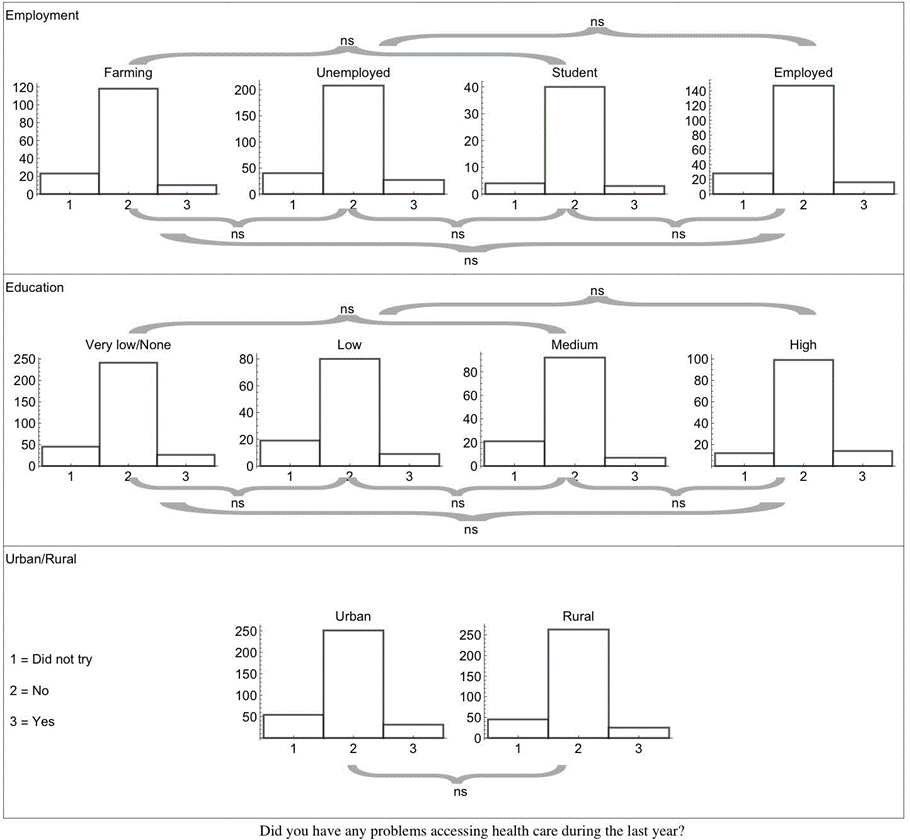


S1A13 Fig: Affordability/cost is a reason I could not get the medical help I need.


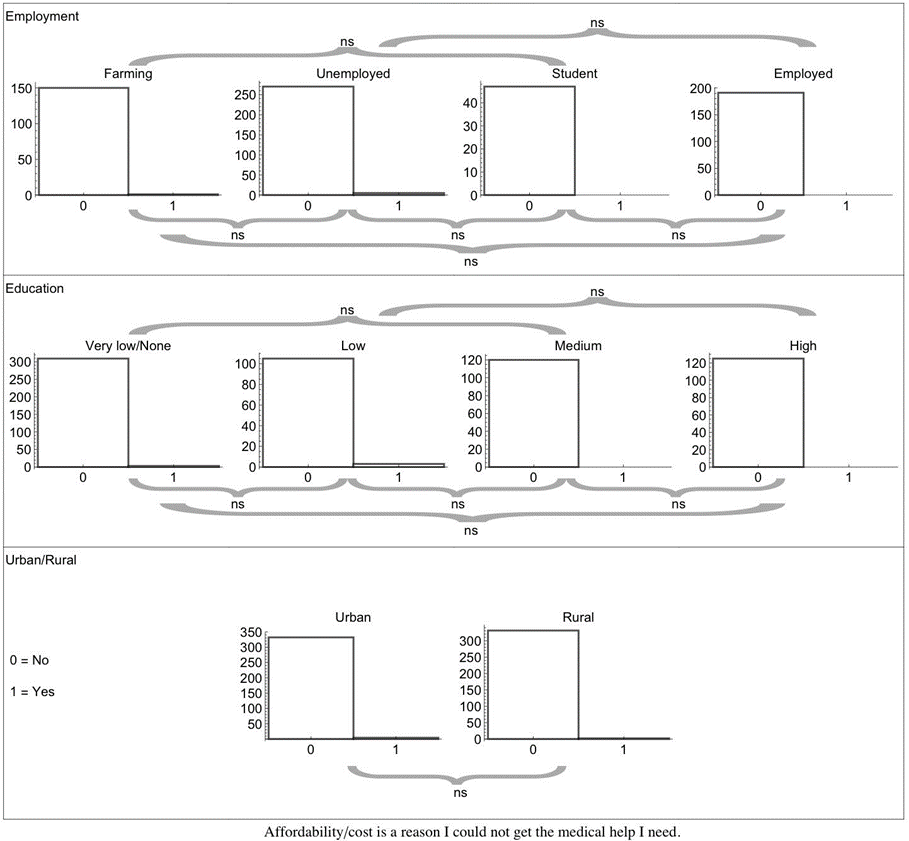


S1A14 Fig: I am able to make and maintain improvements to my house for the next 10 years?


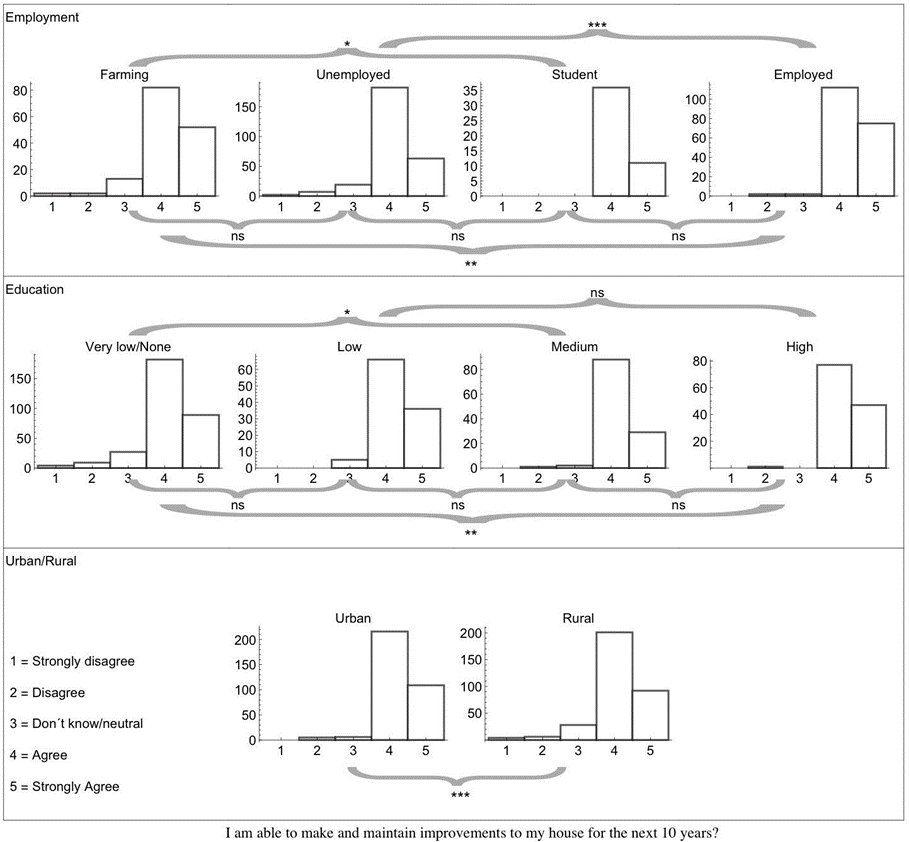


S1A15 Fig: I am able to prevent vector reinfestation during the next 10 years?


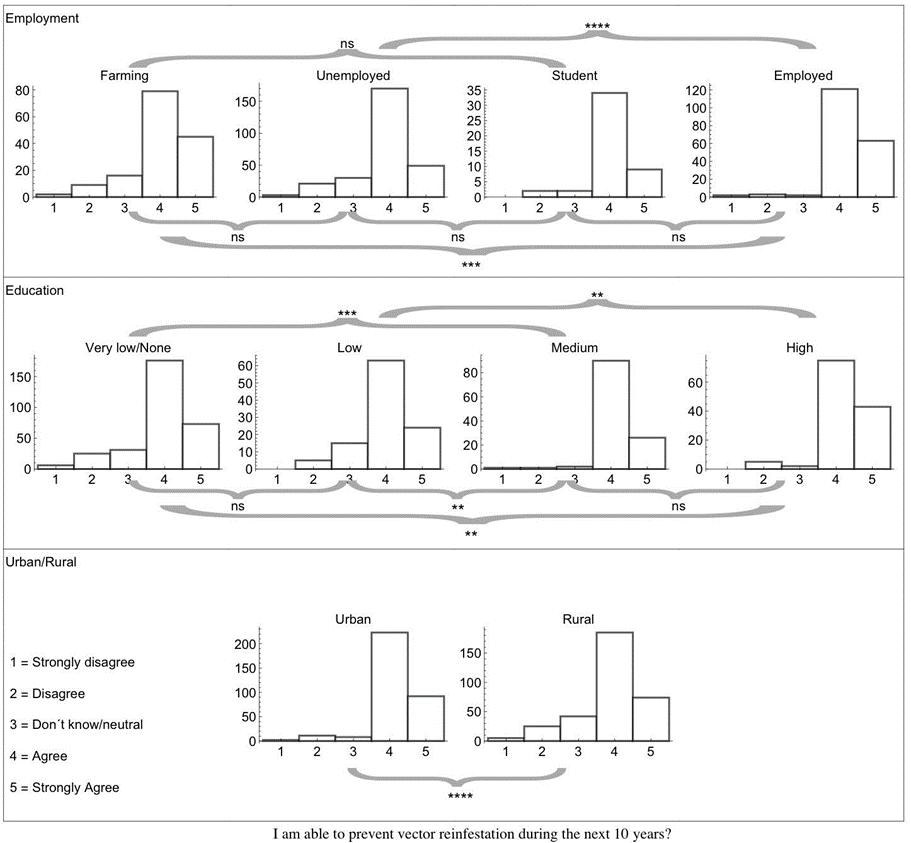


S1A16 Fig: I am able to keep animals far from the house for the next 10 years?


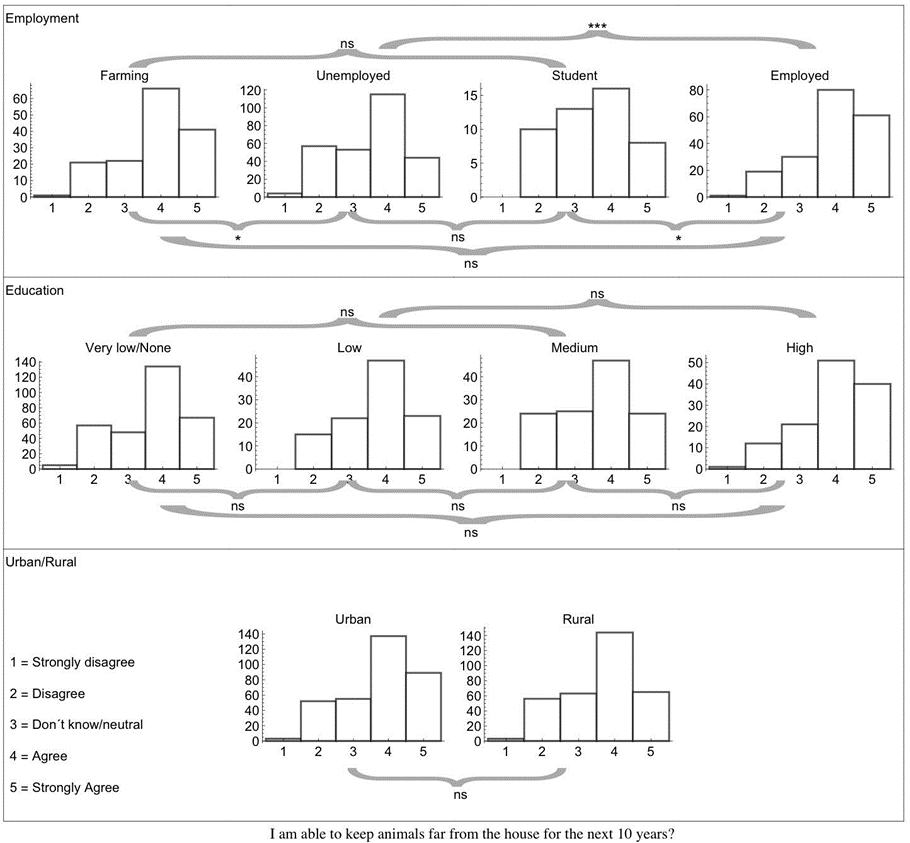


S1A17 Fig: I am able to prevent my family to get infected by CD in the next 10 years?


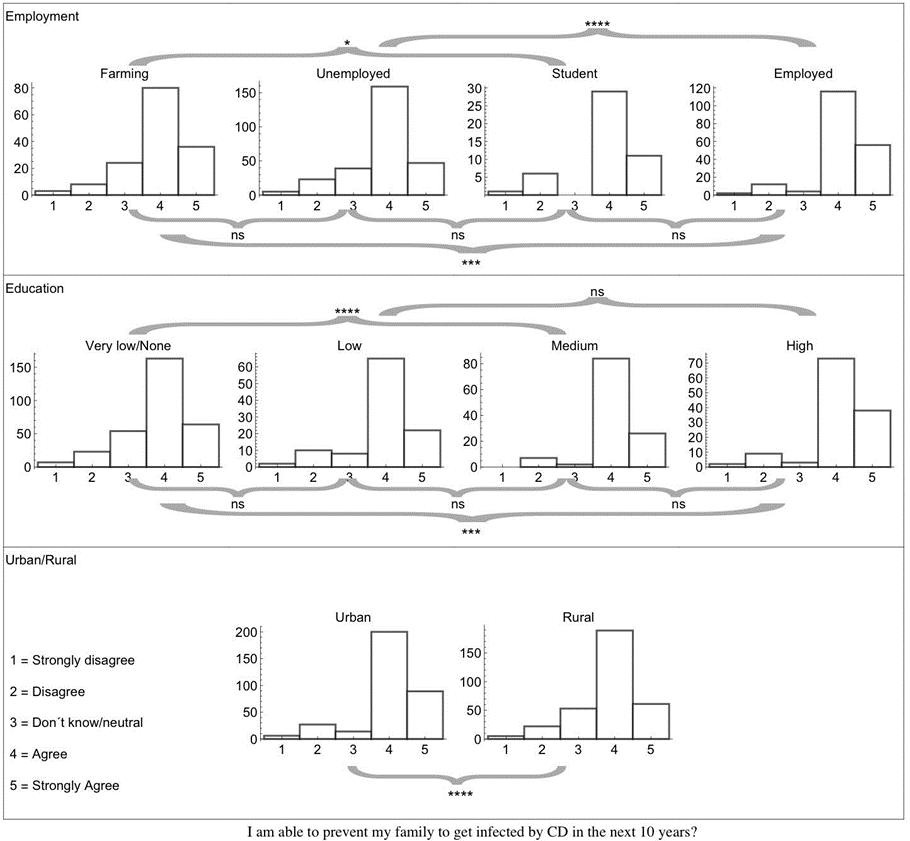


S1A18 Fig: I am able to obtain early diagnosis and treatment if my family gets infected?


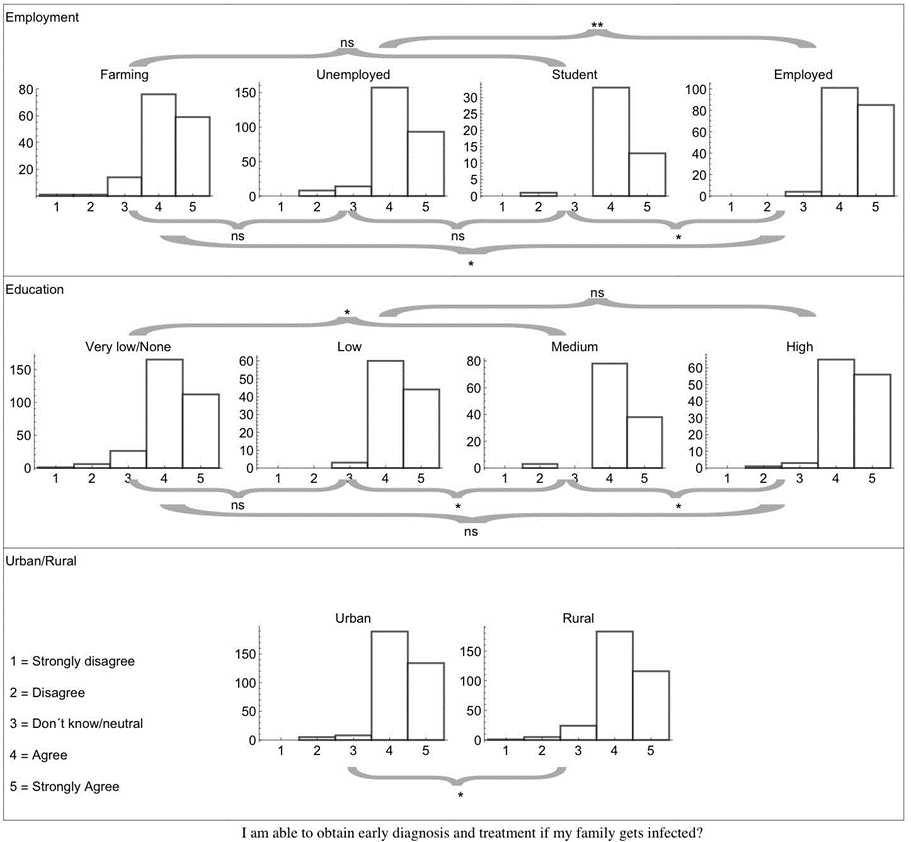


S1B1 Fig: How high is your level of knowledge about CD?


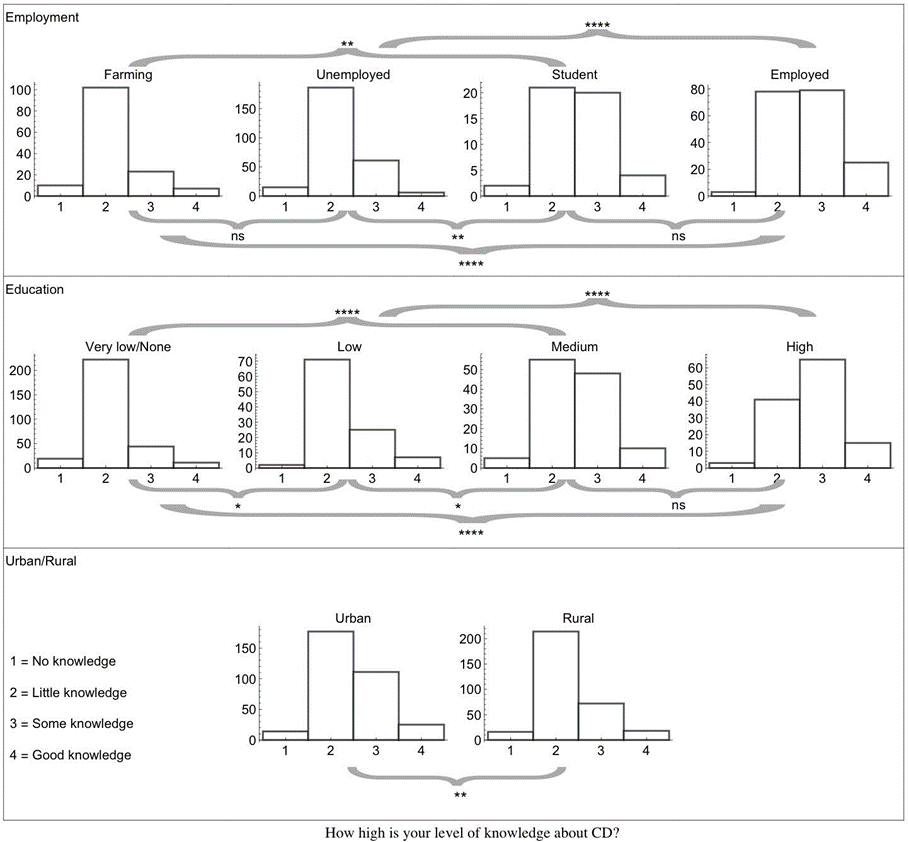


S1B2 Fig: Is CD transmitted by triatomine bugs?


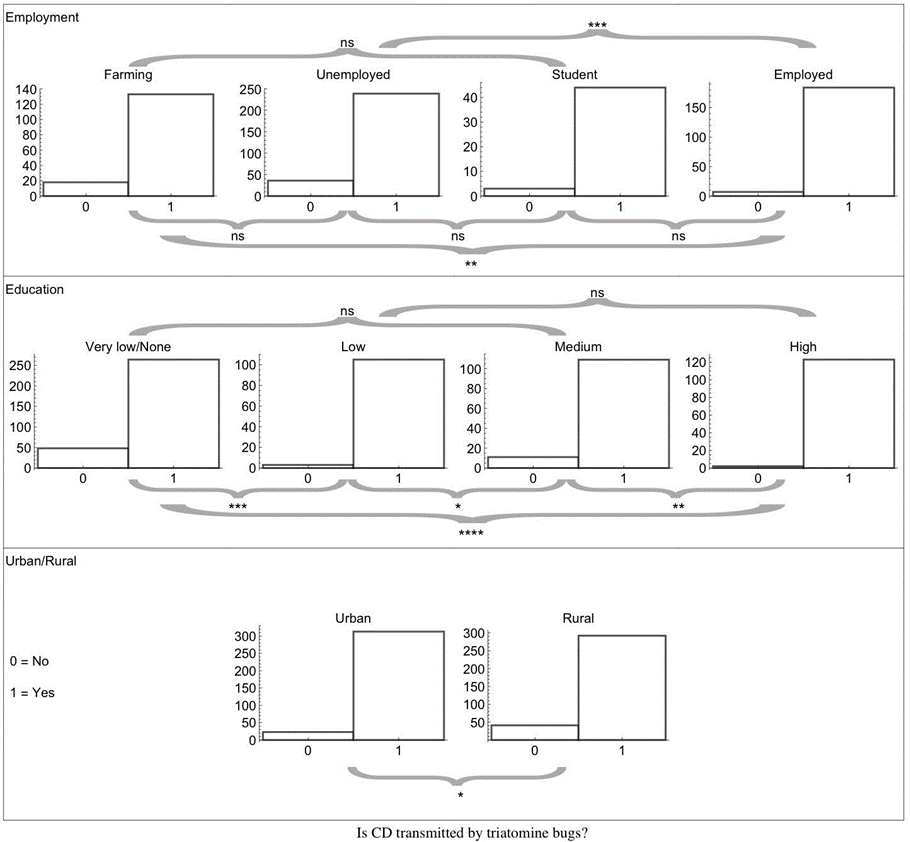


S1B3 Fig: Is CD transmitted by sexual intercourse?


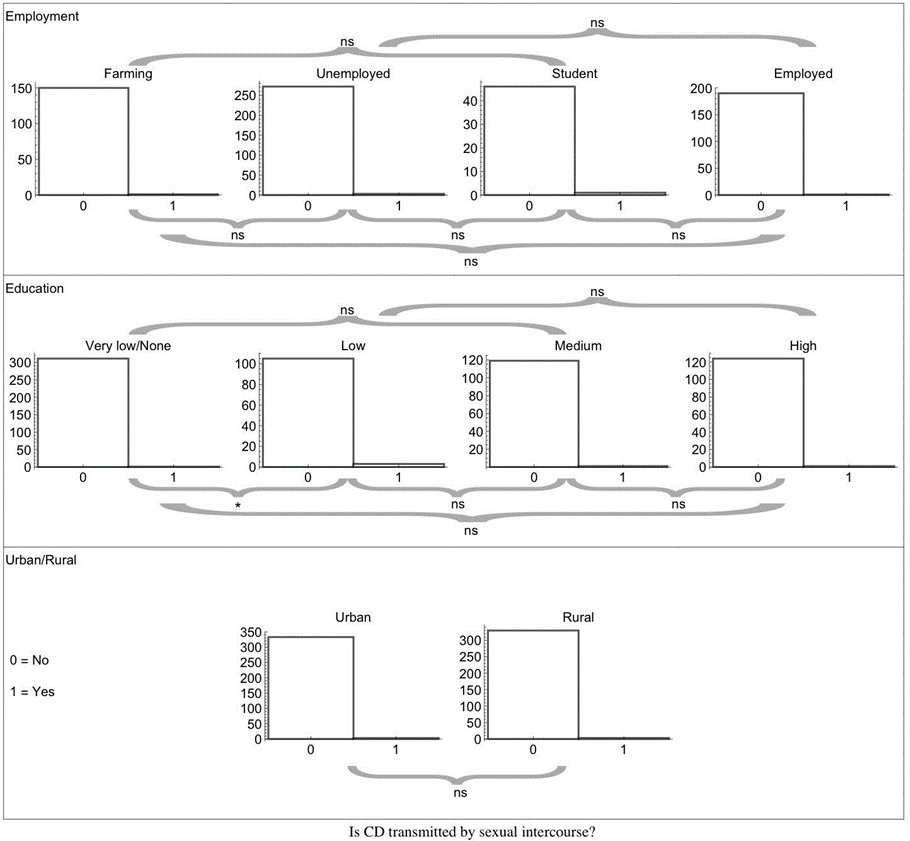


S1B4 Fig: Is CD transmitted by blood and/or organ donations?


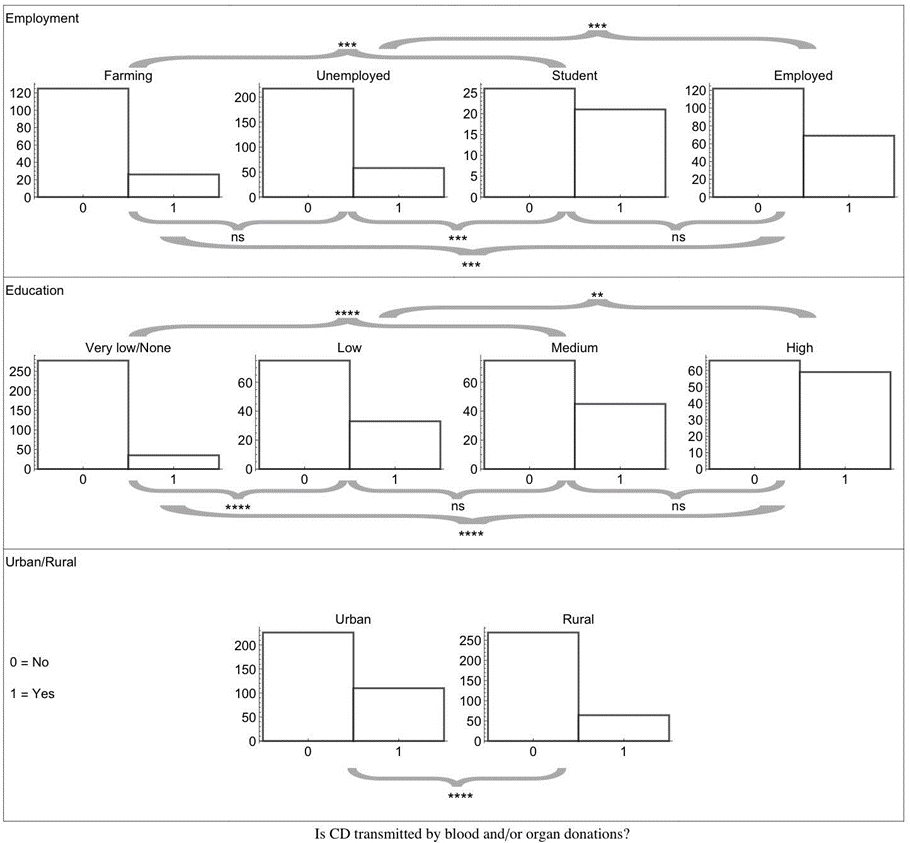


S1B5 Fig: Is CD transmitted by food contaminated with triatomine feces?


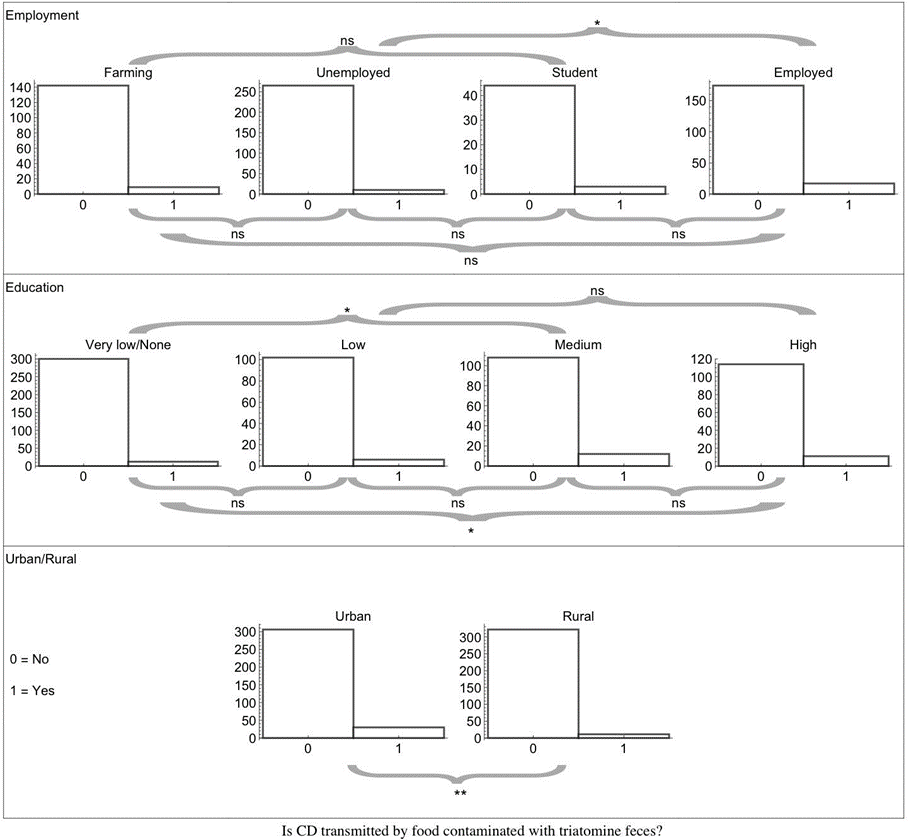


S1B6 Fig: Is CD transmitted by magic?


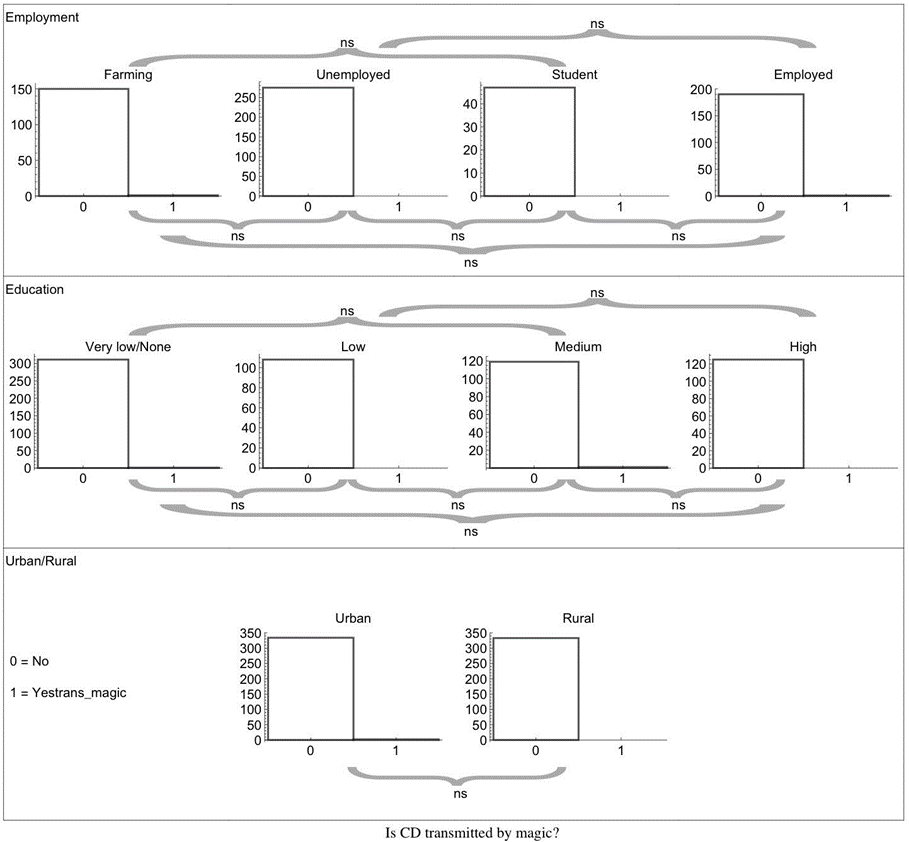


S1B7 Fig: Is CD transmitted from mother to child during pregnancy and/or birth?


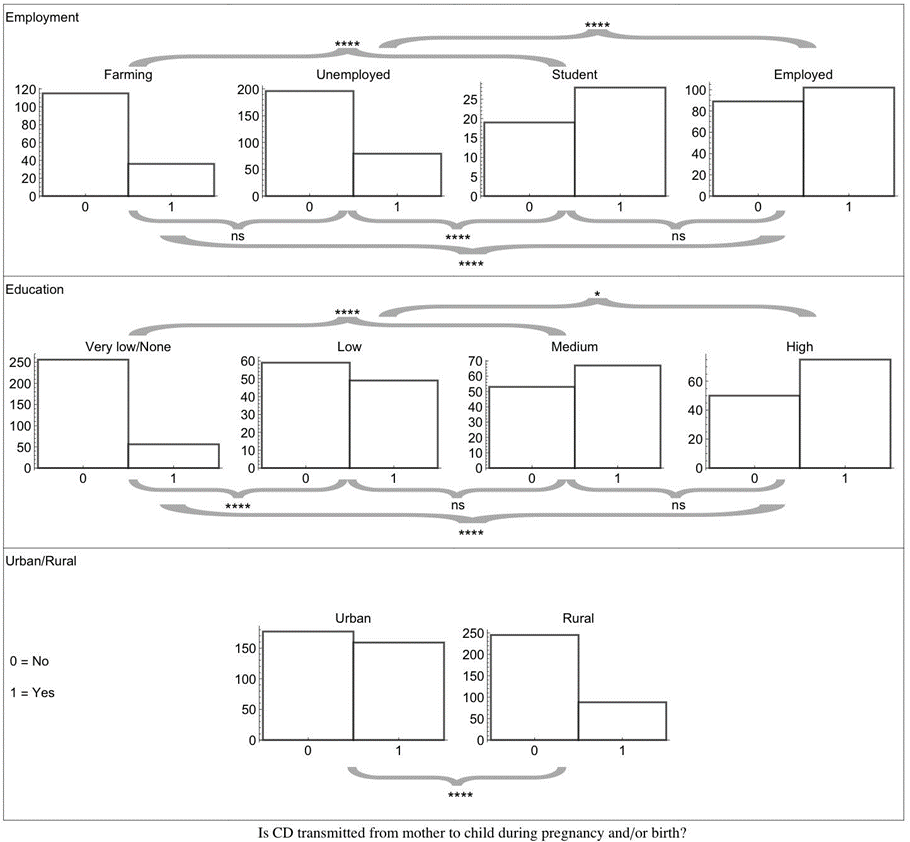


S1B8 Fig: Is CD transmitted by contact with an infected person?


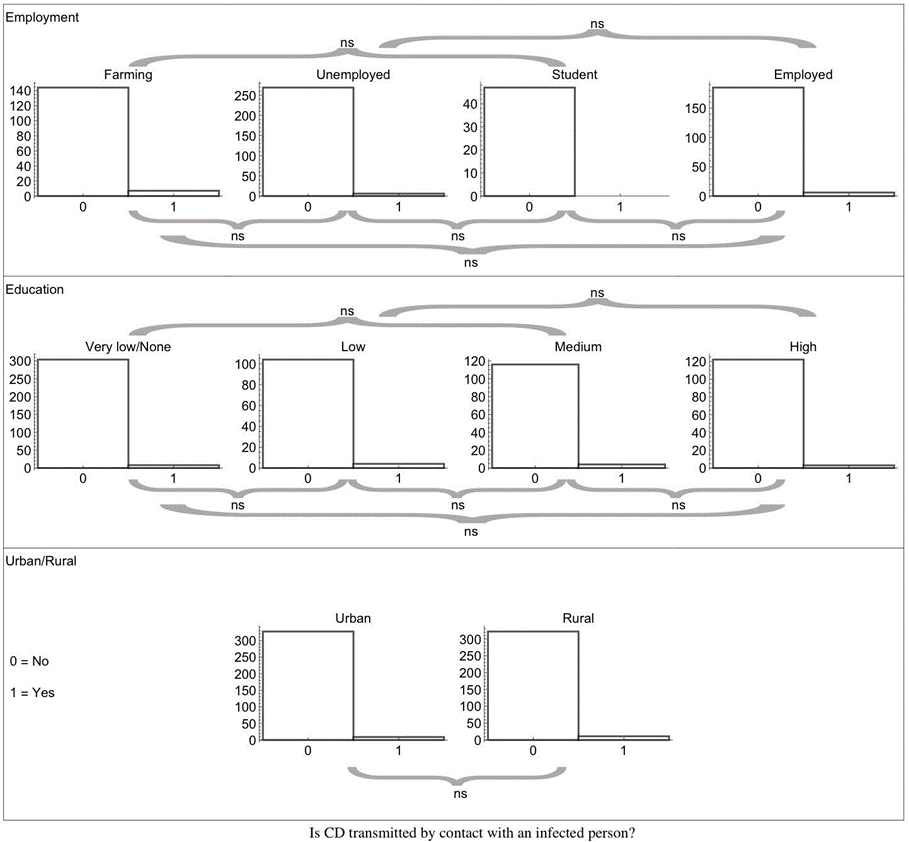


S1B9 Fig: Can CD be prevented by cleanliness?


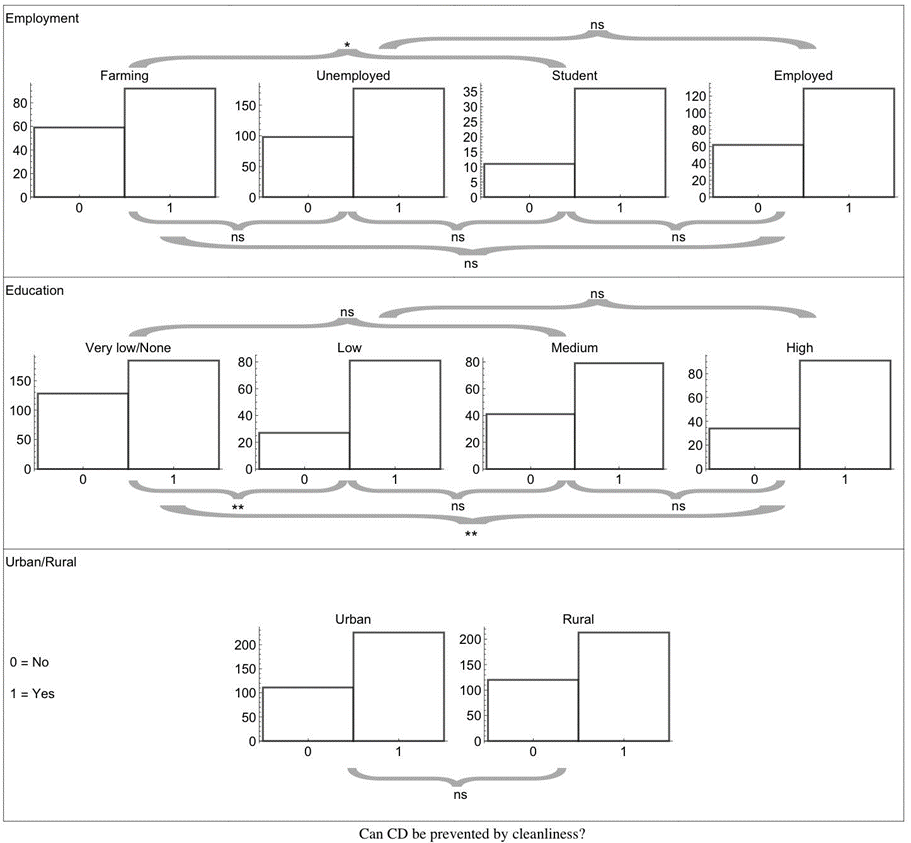


S1B10 Fig: Can CD be prevented by honey and/or bee-glue?


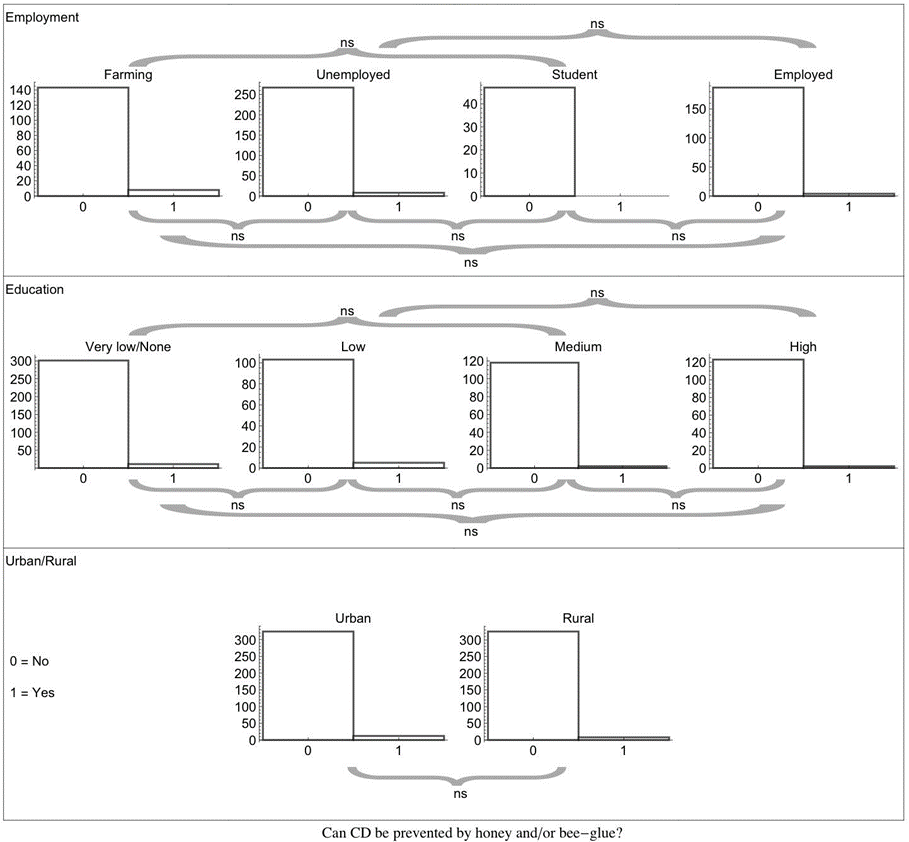


S1B11 Fig: Can CD be prevented by a test during pregnancy?


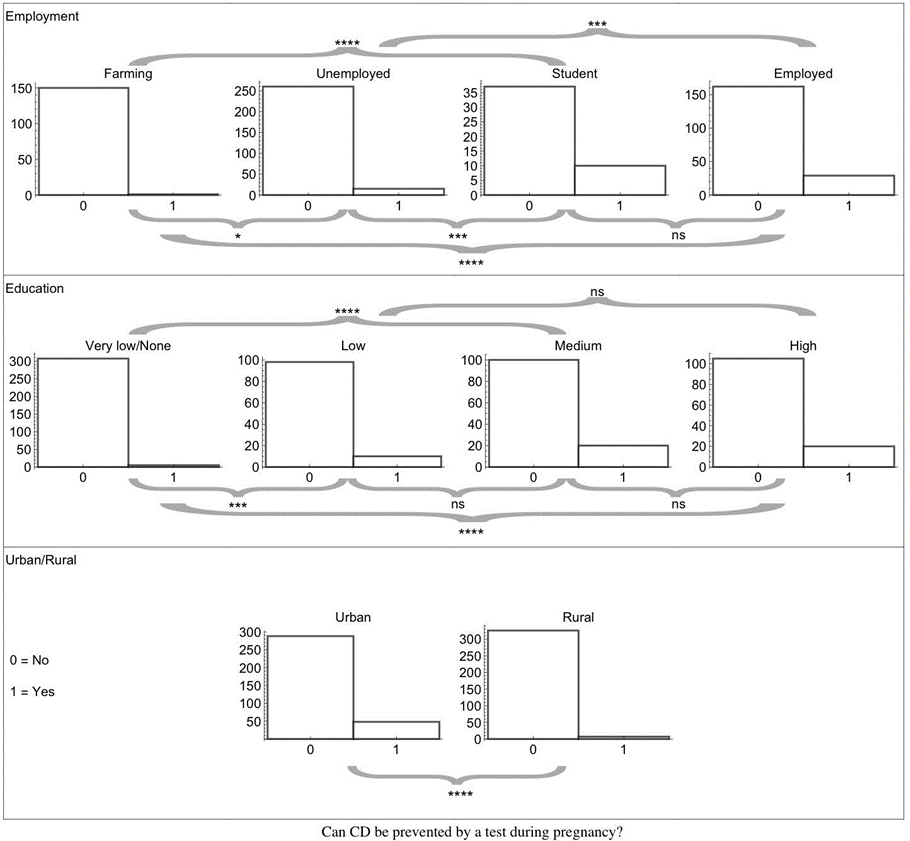


S1B12 Fig: Can CD be prevented by the administration of ivermectin?


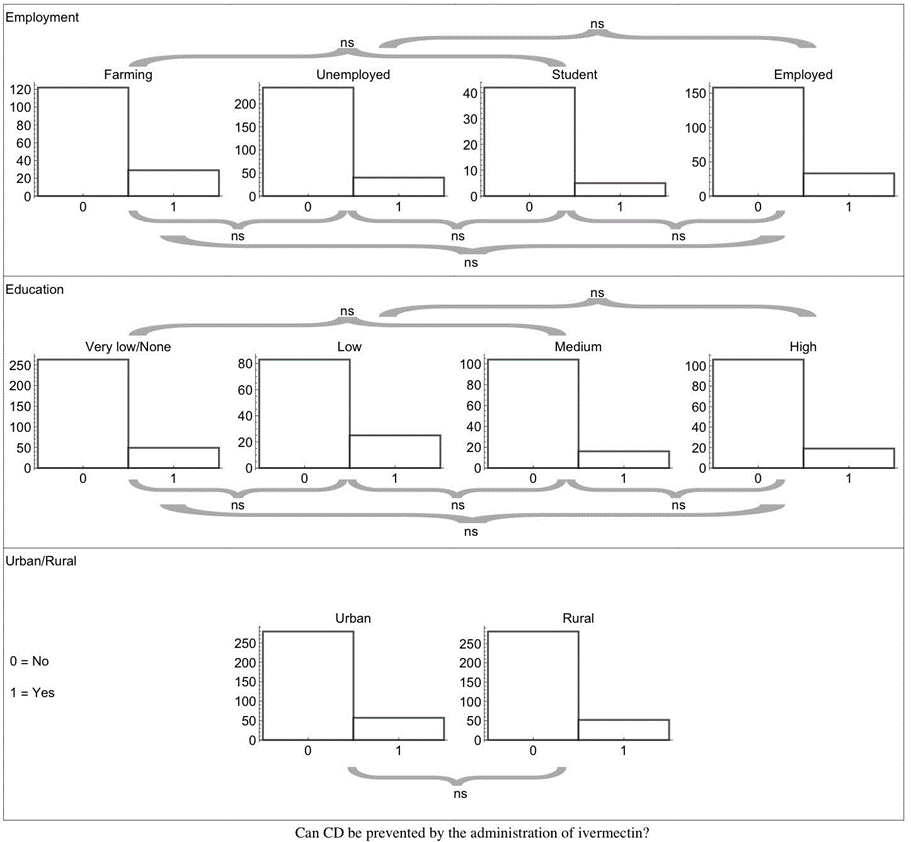


S1B13 Fig: Can CD be prevented by chemical vector control of triatominae?


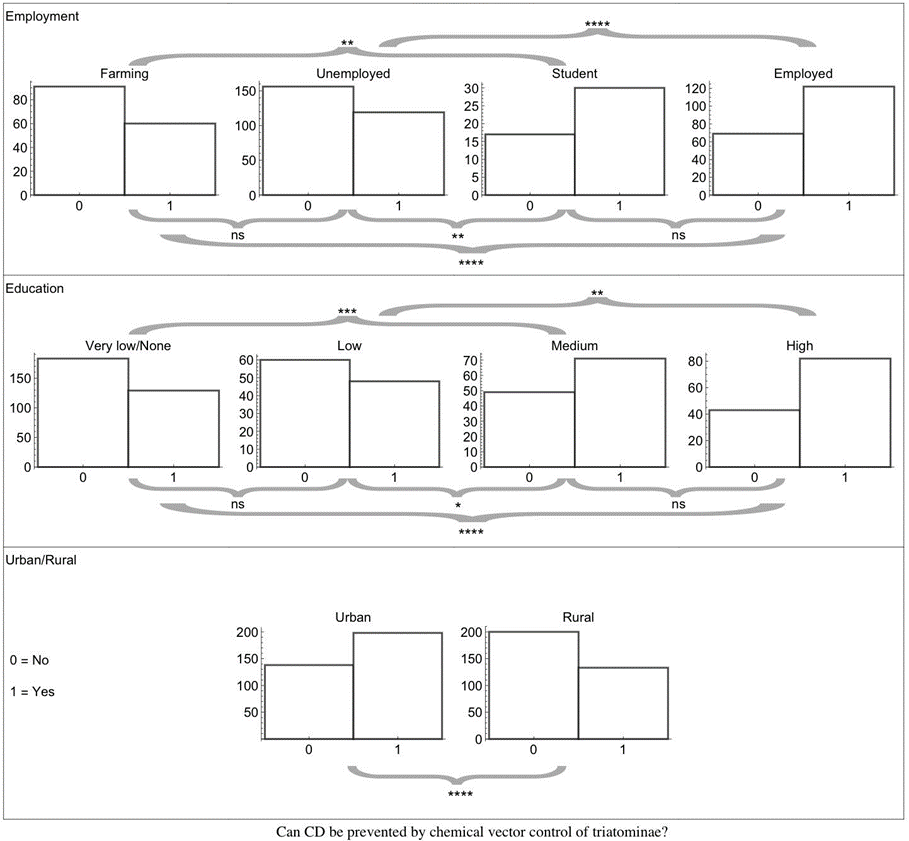


S1B14 Fig: Can CD be prevented by keeping poultry far away from the house?


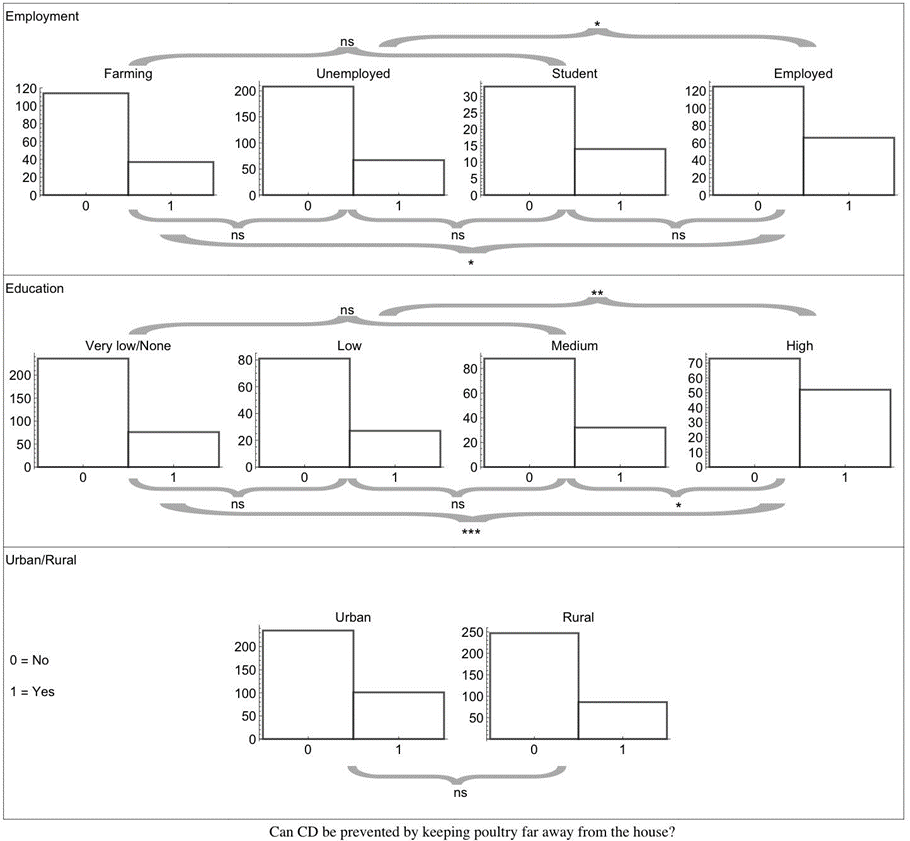


S1B15 Fig: Can CD be prevented by early diagnosis?


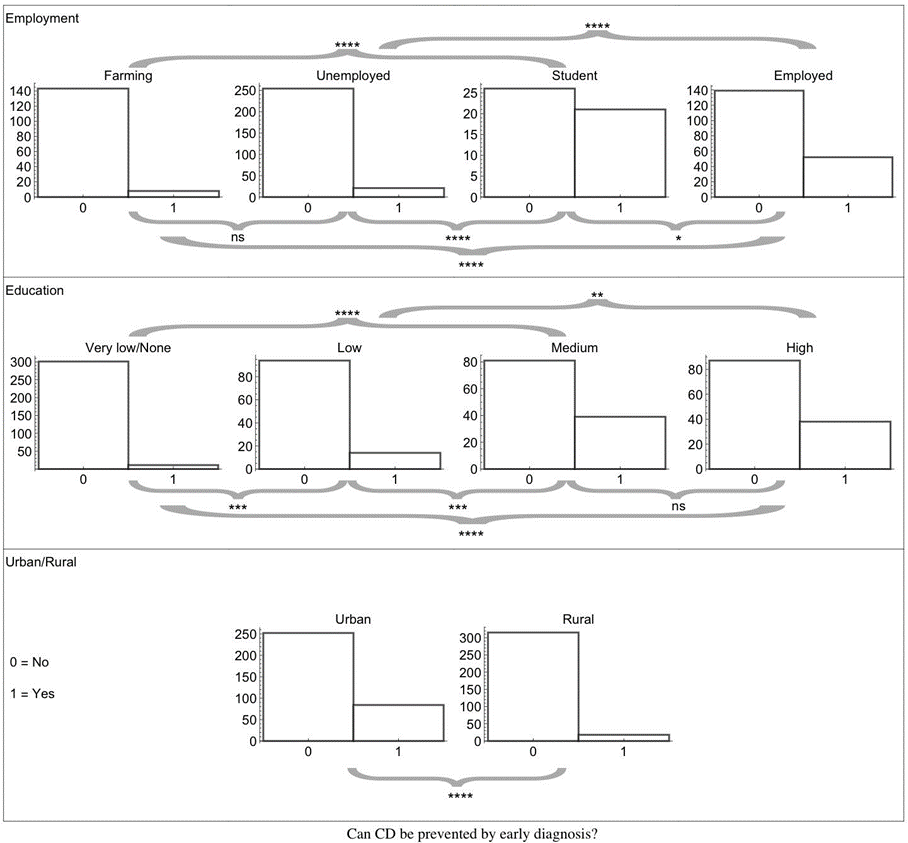


S1B16 Fig: Can CD be prevented by early treatment?


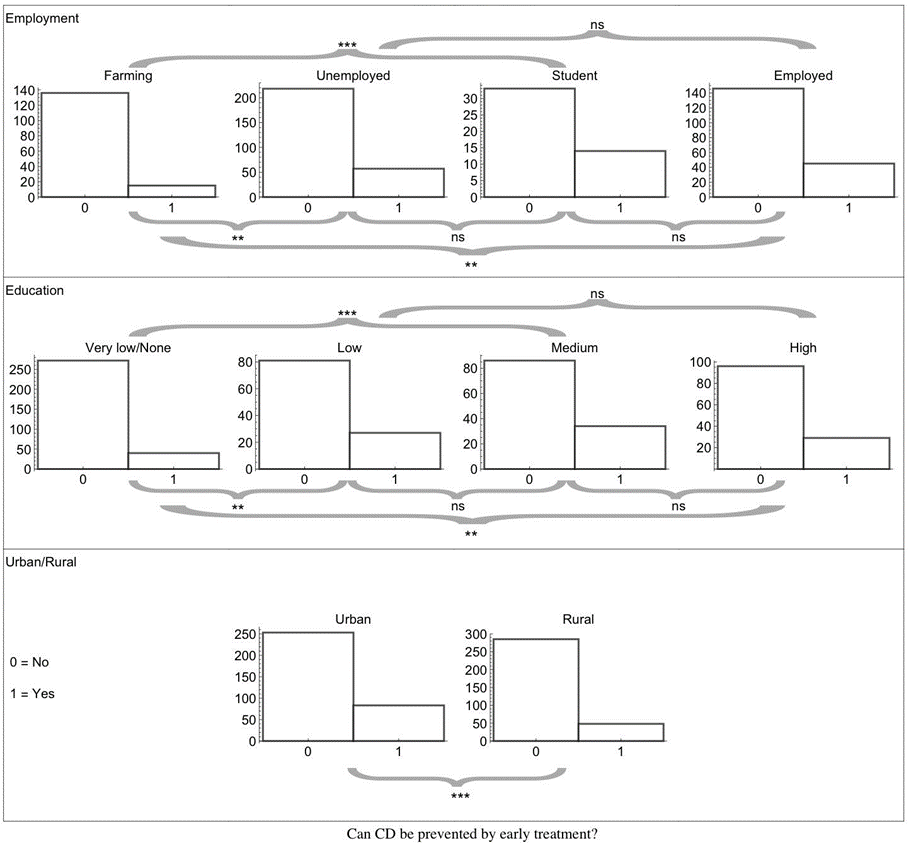


S1B17 Fig: Is prolonged fever a sympton during the initial stages of CD?


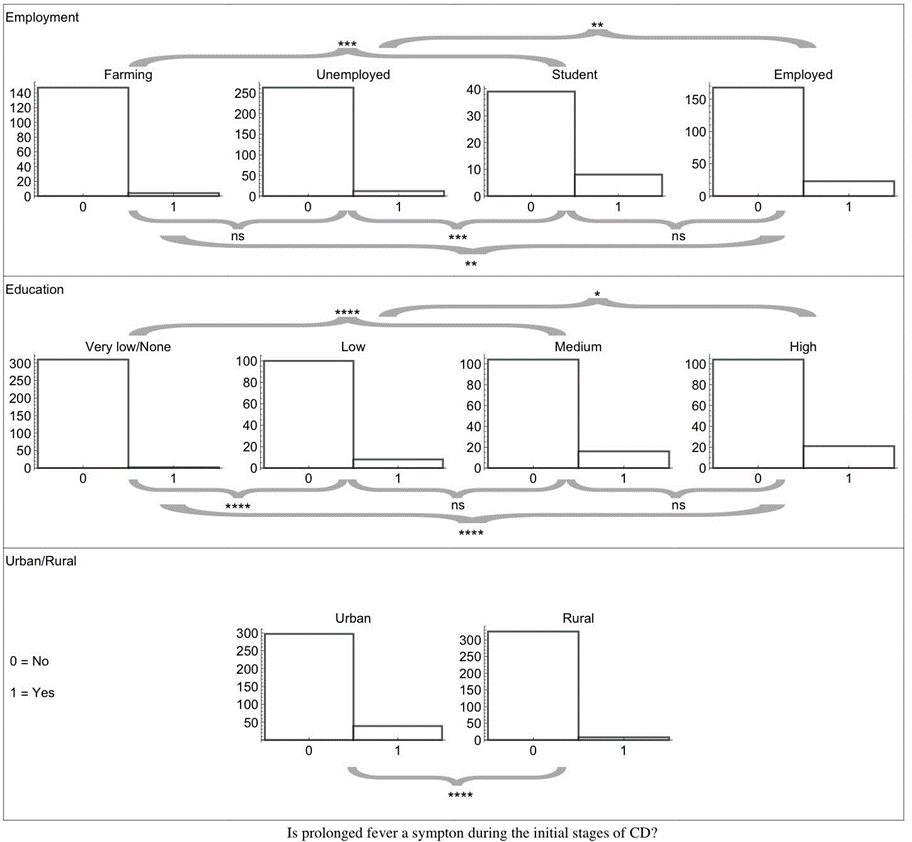


S1B18 Fig: Is swelling of the lymph nodes close to the biting spot a symptom of early CD?


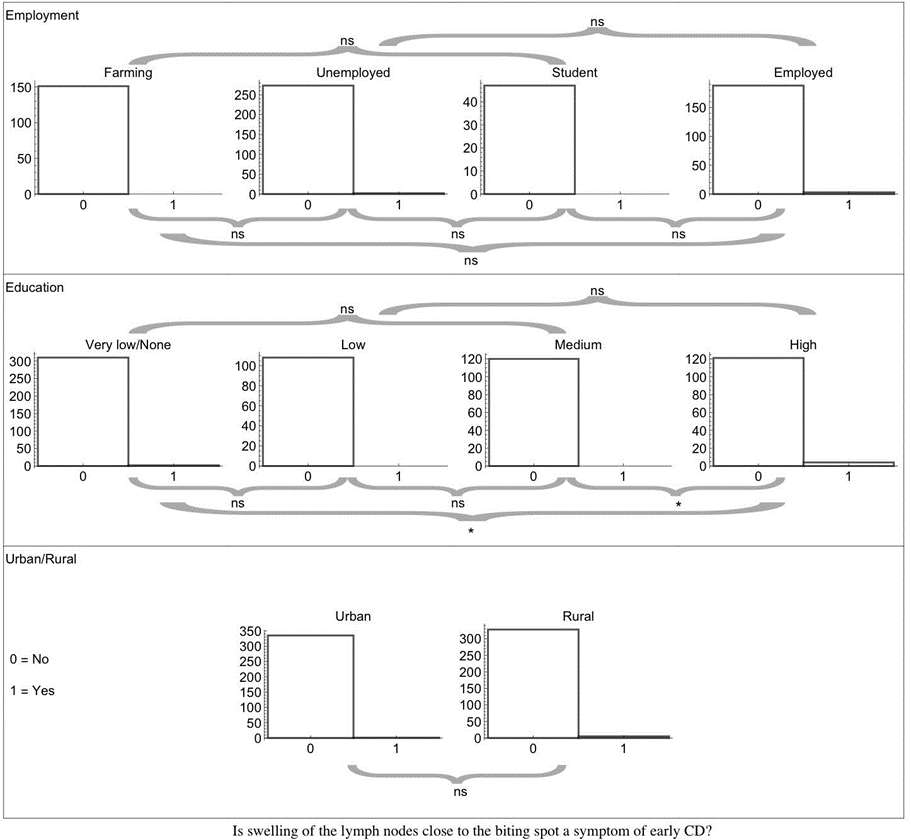


S1B19 Fig: Is persistent swelling of the eye a symptom of the initial stages of CD?


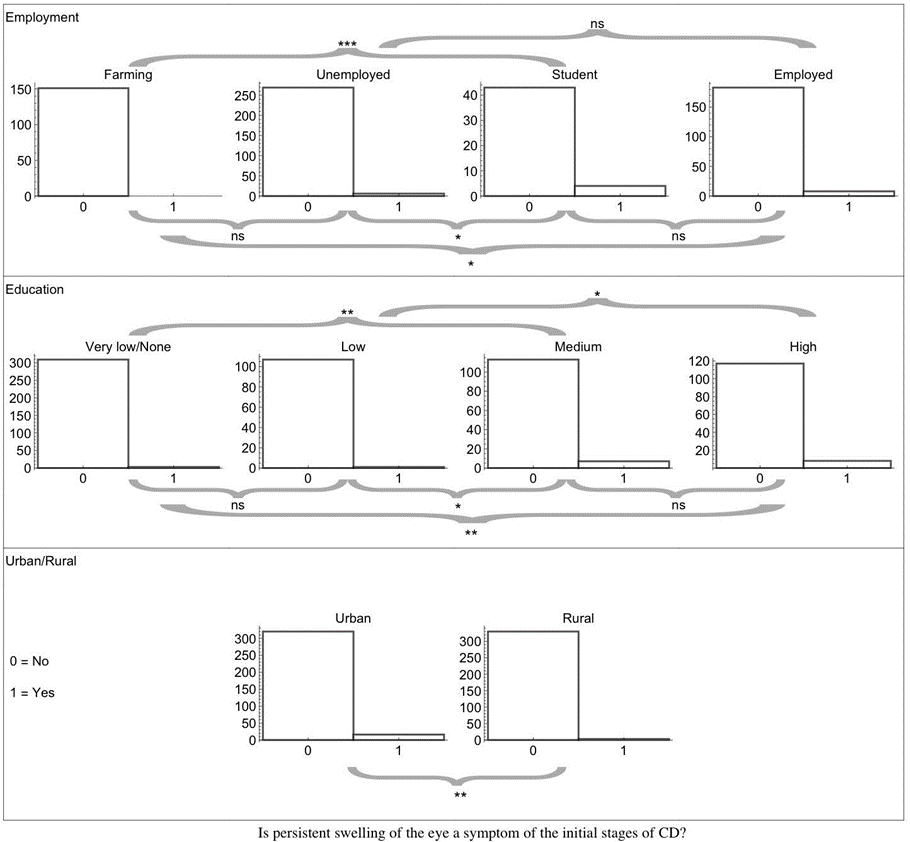


S1B20 Fig: Can the initial stages of CD be asymptomatic?


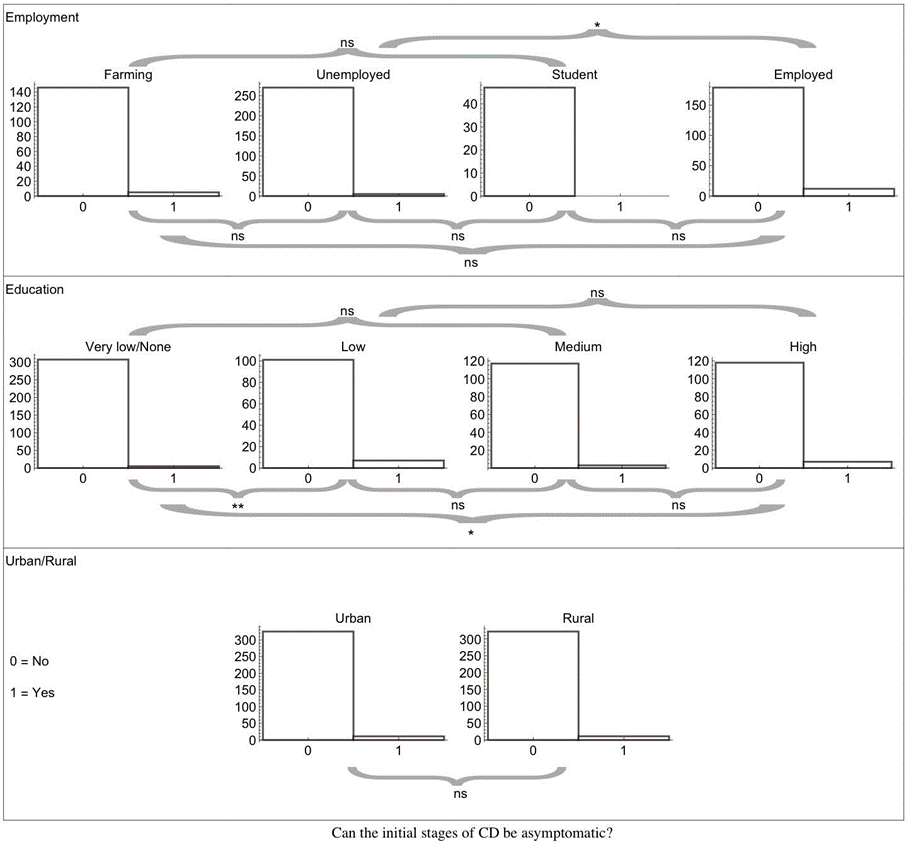


S1B21 Fig: CD is severe?


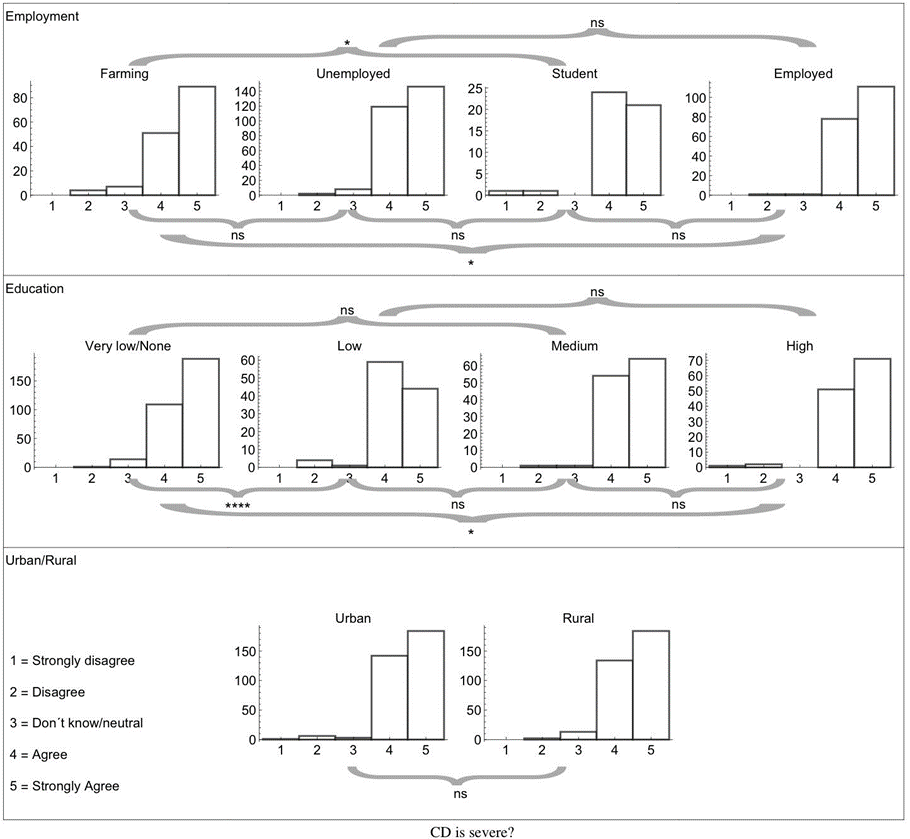


S1B22 Fig: People with CD have a high risk of developing heart complications?


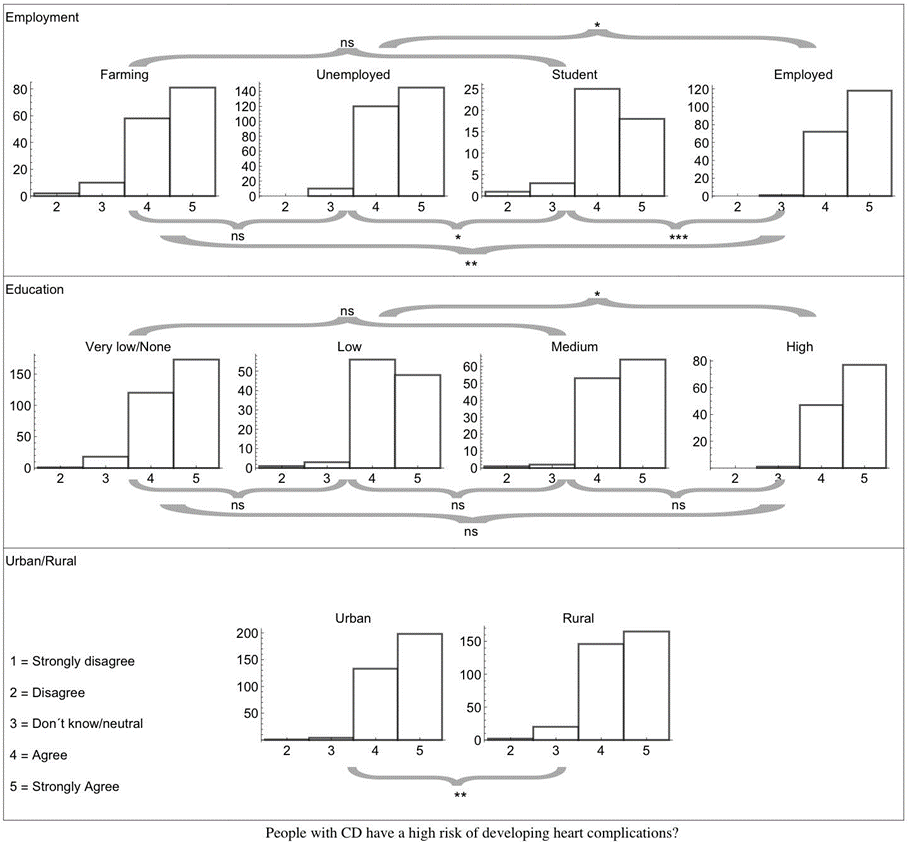


S1B23 Fig: People with CD have a high risk of dying?


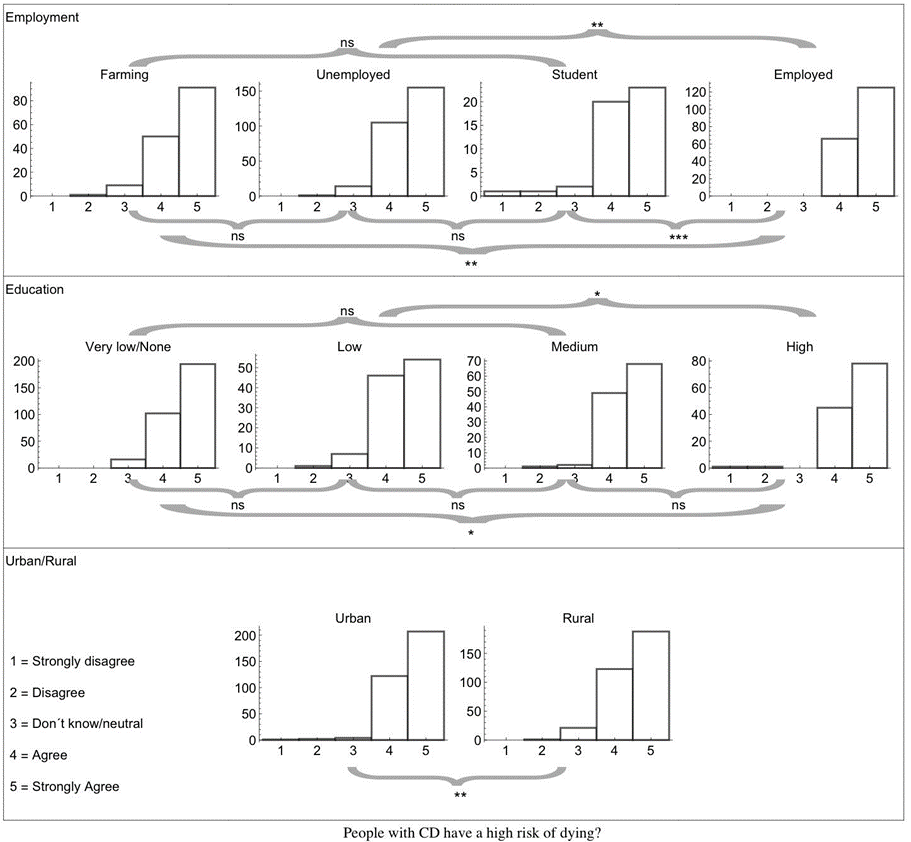


S1B24 Fig: CD leads to sudden, unprecedented severe complications?


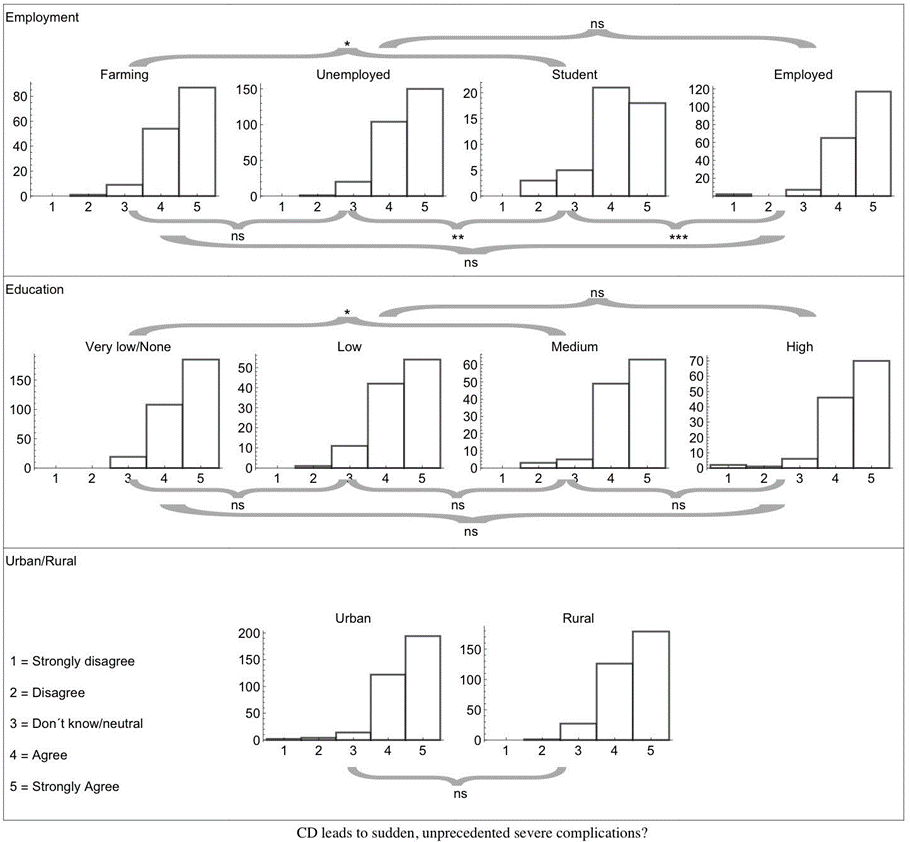


S1B25 Fig: A person with CD will have problems to care for his/her children?


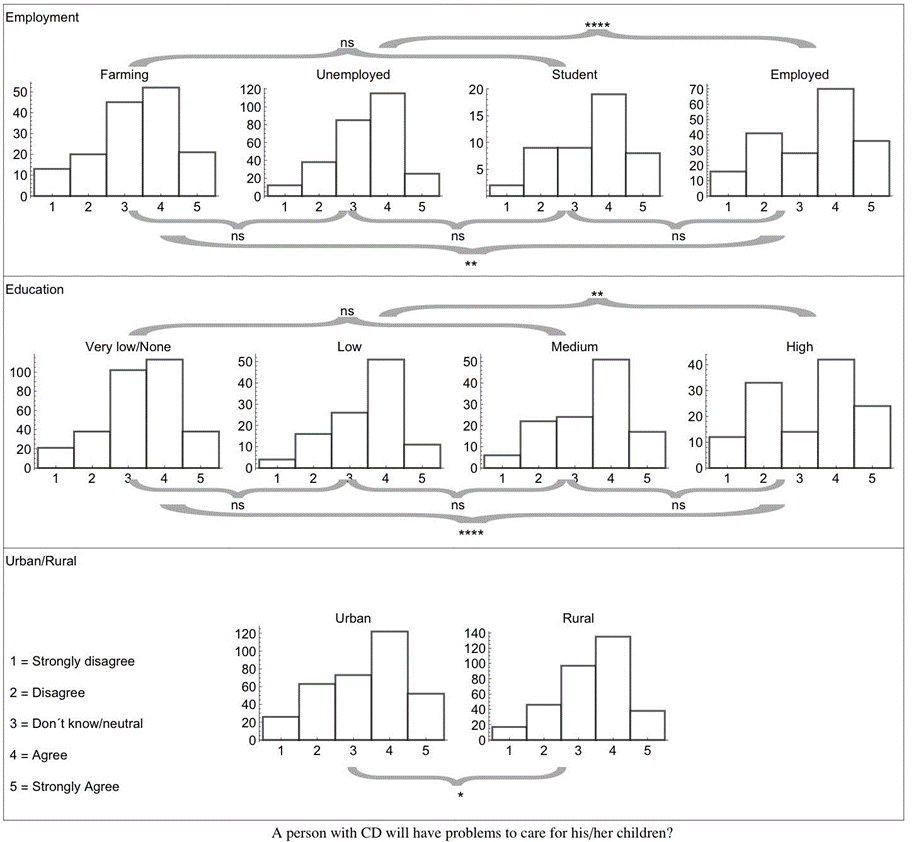


S1B26 Fig: A person with CD will have problems to find/keep a job?


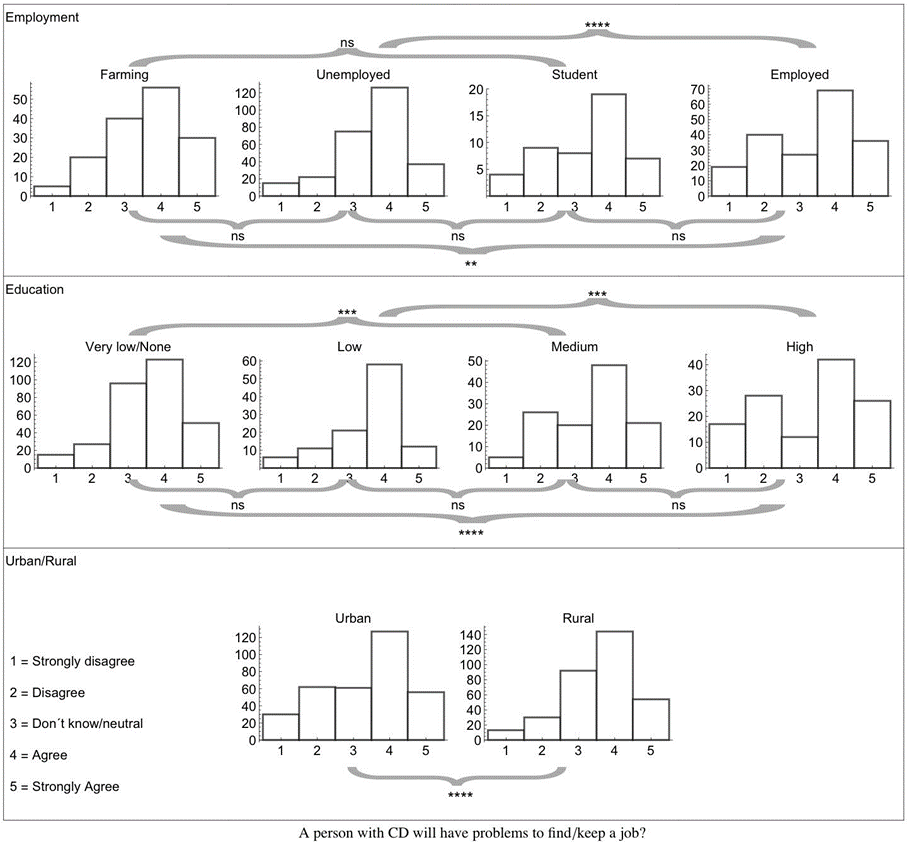


S1C1 Fig: Do you have children?


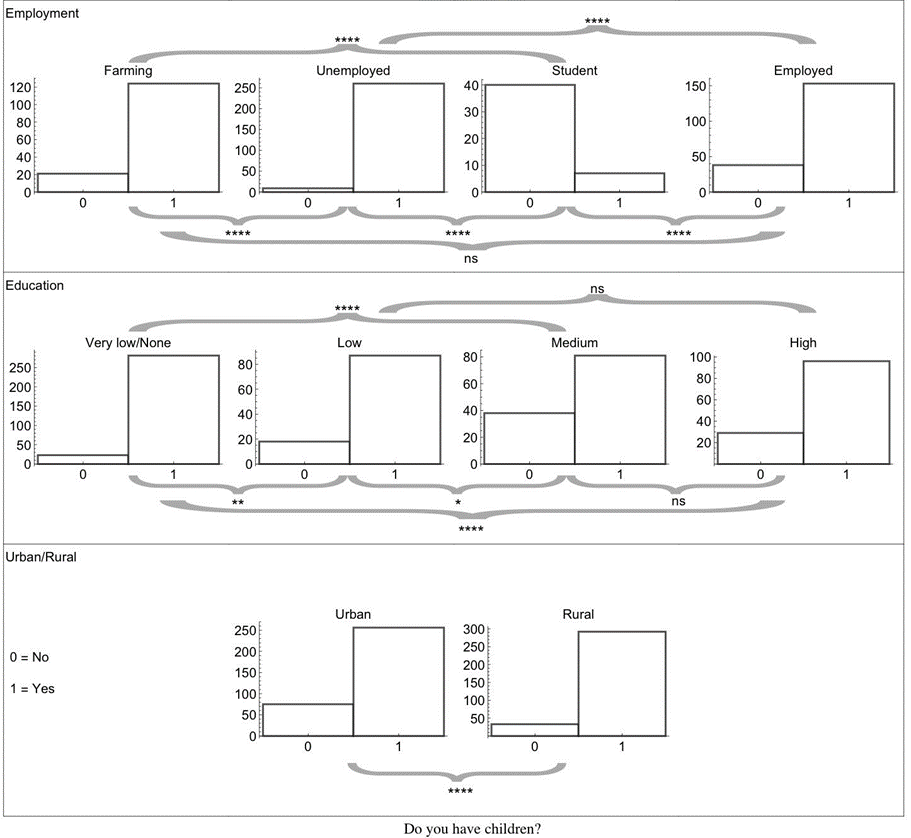


S1C2 Fig: Do you currently have vinchucas in your house?


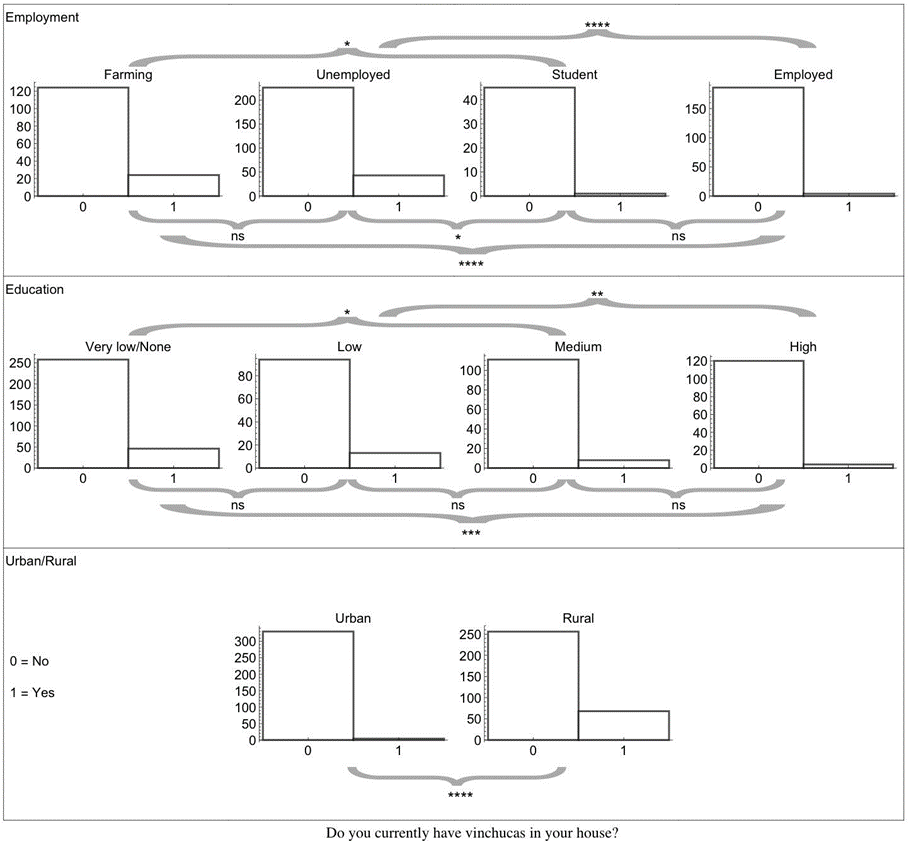


S1C3 Fig: Did you find a vinchuca in your house during the last year?


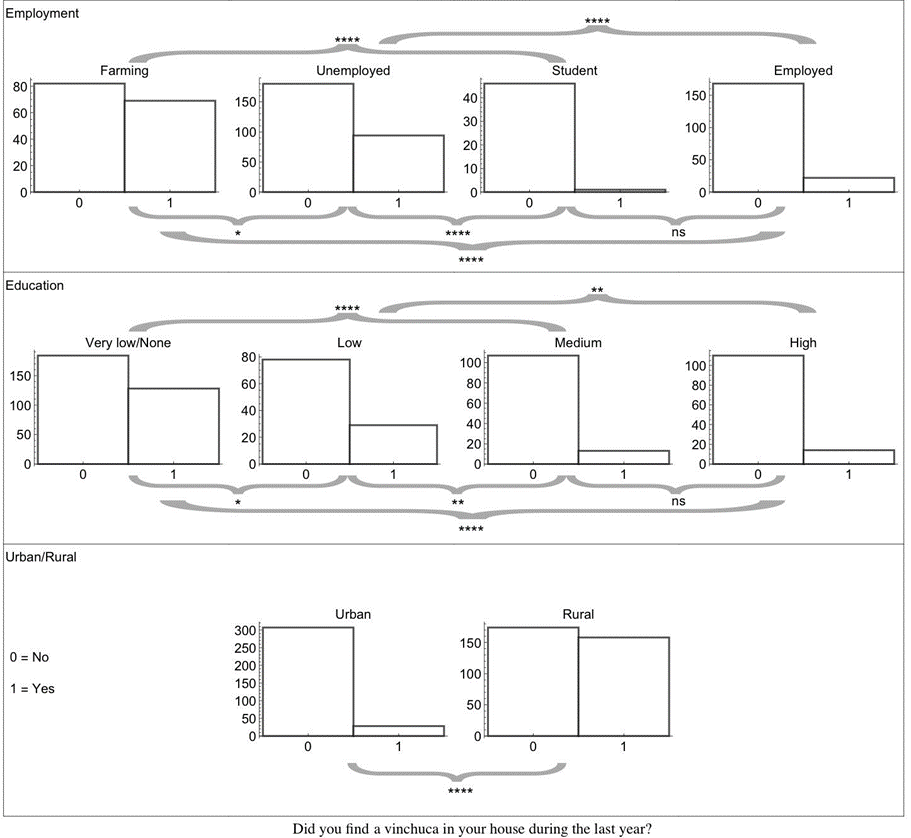


S1C4 Fig: Do you have animals in/near your house?


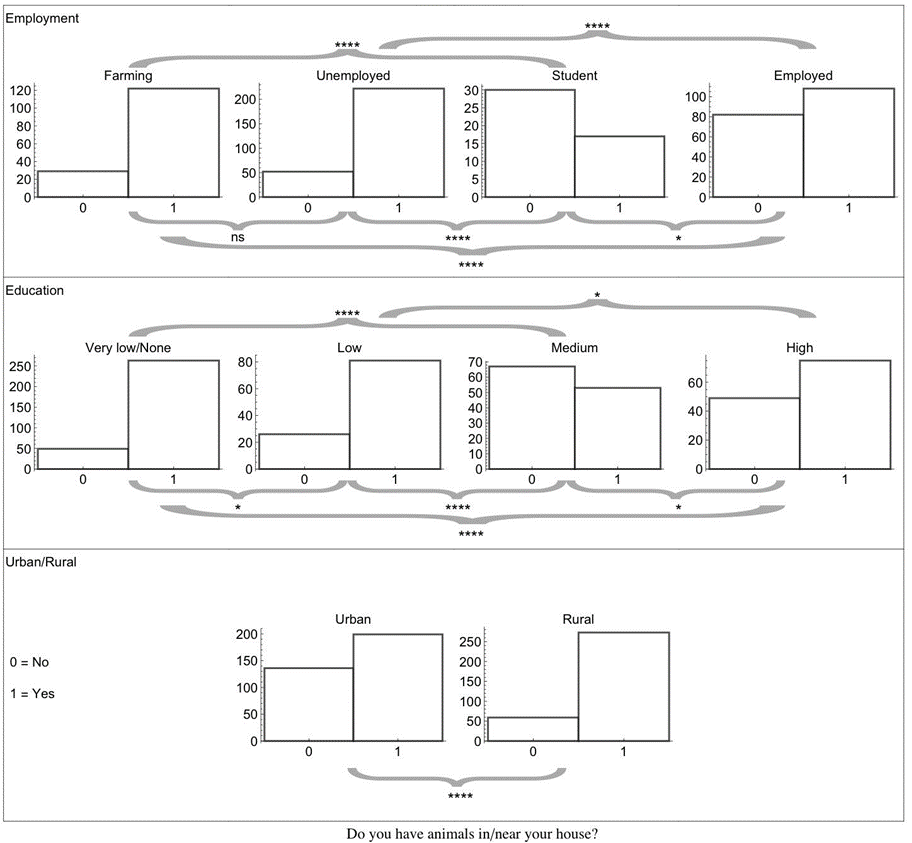


S1C5 Fig: Do you do any prevention against vinchucas?


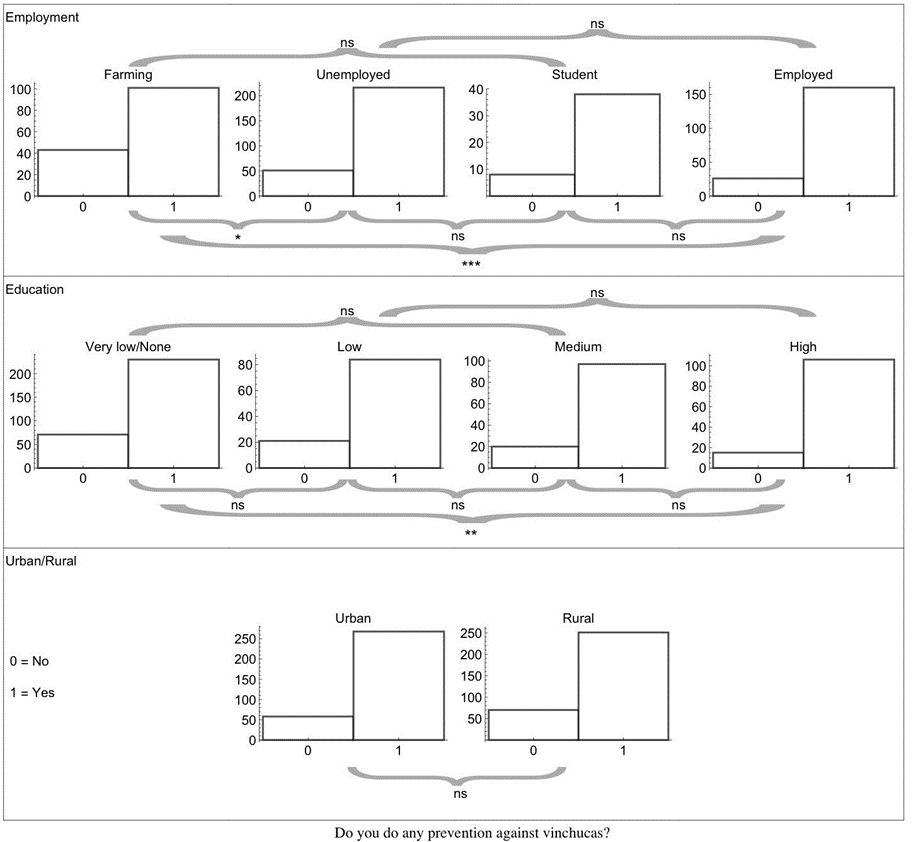

Supplement: S1 Fig — (DOCX) [file pntd.0008752.s007.docx]
